# Detection and characterization of simvastatin and its metabolites in rat tissues and biological fluids using MALDI high resolution mass spectrometry approach

**DOI:** 10.1038/s41598-022-08804-x

**Published:** 2022-03-19

**Authors:** Wencui Yin, Reem I. Al-Wabli, Mohamed W. Attwa, A. F. M. Motiur Rahman, Adnan A. Kadi

**Affiliations:** Department of Pharmaceutical Chemistry, College of Pharmacy, King Saud University, Riyadh, 11451 Kingdom of Saudi Arabia

**Keywords:** Drug discovery, Medicinal chemistry, Drug discovery and development, Bioanalytical chemistry, Mass spectrometry

## Abstract

Simvastatin (SV) is a hypolipidemic agent, and it is the 2nd most widely prescribed lipid-lowering drug. Here, the detection and characterization of SV and its metabolites was studied in selected organs/tissues (lung, liver, brain, heart and kidney) and biological samples (blood, urine and feces) of rats. MALDI Orbitrap MS was used as a high-resolution mass analyzer. 2,5-Dihydroxybenzoic acid (DHB) and 1,5-diaminonaphthalene (DAN) were used as matrices. Several sample loading methods onto the MALDI plate were attempted and dried droplet method was found to be superior. Two different cell disruption methods, pulverization and homogenization, were also evaluated for the optimum sensitivity in MALDI. Pulverization allowed the detection of more metabolites in all organs except the liver, where homogenization led to the detection of more metabolites. Altogether, 13 metabolites were detected, and one metabolite tentatively identified as a reduced product is being reported for the first time. SV and its metabolites were distributed to all the tissues studied except the brain. Overall, the results implied that the pulverized samples were more uniform and larger in surface area, resulting in their more efficient and complete extraction during sample preparation. As shown in the present study, MALDI Orbitrap MS is a useful tool to study drug and metabolite detection and characterization.

## Introduction

Drug distribution is a very important aspect to consider in the drug discovery and development process^1^. Knowledge of drug distribution to the tissues is linked to many other areas of drug development^2,3^. Distribution, which is one of the basic processes in the ADME (absorption, distribution, metabolism, and excretion) sequence of drugs, plays a crucial role in shaping the pharmacological and toxicological responses and pharmacokinetic behavior^4^. For a drug or metabolite to elicit its pharmacological effects after absorption, it must reach the targeted biological site of action^5–7^. Unexpected secondary pharmacology and toxicity can occur either from the accumulation or localization of the parent drug or its metabolites in tissues expressing nontargeted biological receptors^8^. Thus, the knowledge gained about the mechanisms and factors involved in distribution must be constantly applied to design new drugs and carrier systems to ensure the specific delivery of the drug to a particular organ or tissue, optimize the response, increase efficiency and reduce potential toxicity^4^.

**Figure 1:**
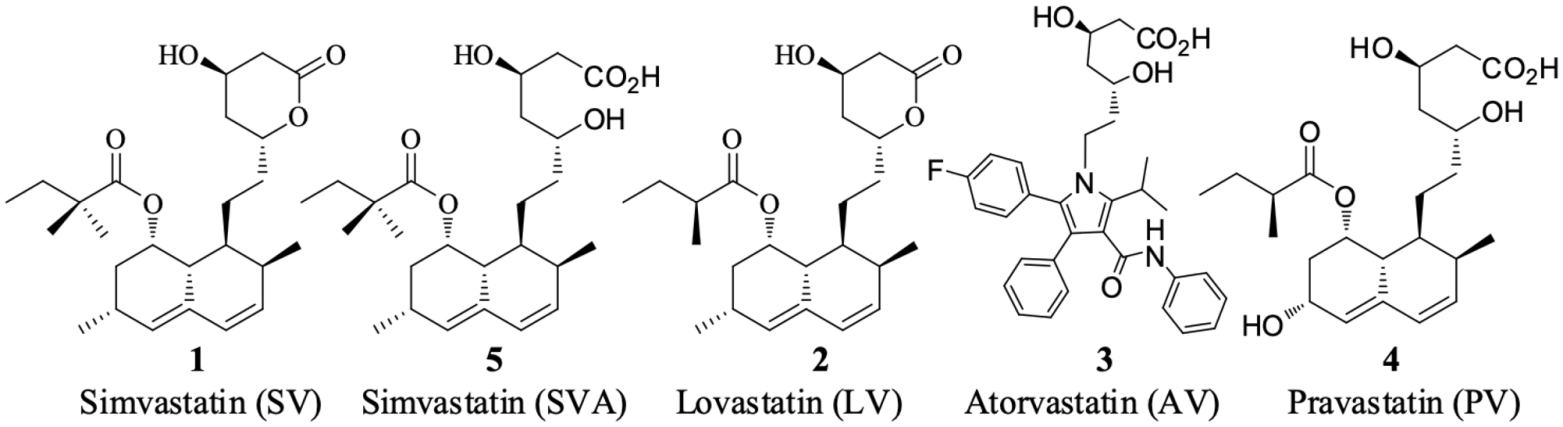
Chemical structures of some known statins.

Simvastatin (**SV**, **1**) [1S-[1R,3R,7S,8aR(2S*, 4S*),8aR]]-1,2,3,7,8,8a-hexahydro-3,7-dimethyl-8-[2-(tetrahydro-4-hydroxyl-6-oxo-2H-pyran-2-yl)ethyl]-1-naphthalenyl-2,2-dimethyl butanoate] (Fig. 1) is a semisynthetic derivative of lovastatin (**LV**, **2**)^9^, which is biosynthetically produced from the fungus *Aspergillus terreus*^10,11^ and has a high log *P* value (log *P* = 4.39), resulting in high hepatic extraction and high efficacy in controlling cholesterol synthesis^12^. Together with atorvastatin (**AV**, **3**), these compounds are the two most commonly sold drugs worldwide for the clinical treatment of hypercholesterolemia^11,13^. Similar to other statins, SV has two efficient moieties, a dihydroxyheptanoic acid unit (pharmacophore) and a ring system with lipophilic substituents. It contains a modified hydroxyglutaric acid component, which structurally resembles the 3-hydroxyglutaryl unit of both the substrate (HMG CoA) and the mevaldyl CoA transition state intermediate^14^, making SV a potent competitive inhibitor of HMG-CoA reductase^15^.

Despite the potency of SV in lowering cholesterol levels, it was reported to have some rare serious side effects, including muscle breakdown, liver problems, and increased blood sugar levels^16–21^. Common side effects include constipation, headaches, and nausea. A lower dose may be needed in people with kidney problems^22^. SV was shown to induce harmful effects in unborn babies when taken during pregnancy^22,23^, and it should not be used by nursing mothers. Therefore, a thorough study of its metabolism and distribution is necessary to understand the mechanism underlying its pharmacological effect and its potential side effects. After a literature survey, we found a study of the distribution of SV and its primary metabolite simvastatin acid (SVA) in various rat tissues following the administration of a single oral dose of SV using an enzyme assay protocol published in 1989^24^, In that study, the active drug (SVA) presented in each tissue was directly determined by measuring the inhibitory activity against the target enzyme (HMGR) relative to an extract of a whole tissue homogenate. However, the inhibitory activity against HMGR might also be derived from metabolites other than SV or SVA, which retain the portion that resembled HMG-CoA, therefore lacking specificity in detection. In the following year, Vickers et al*.* studied the distribution of SV and SVA in selected rat tissues with dosing of radioisotope-labeled [^14^C] SV using HPLC-UV^25^. Notably, the authors detected only SV and SVA. Since the reported studies are limited in scope, more investigations of the distribution of SV and/or its metabolites in a broader manner might be valuable. Recently, matrix-assisted laser desorption ionization (MALDI) using a TOF mass spectrometer^26,27^, ion trap mass spectrometer^28–30^, hybrid ion trap-ToF mass spectrometer^31–33^, and MALDI Orbitrap MS have been used to assess drug distribution^34^, besides conventional LC–MS/GC–MS for SV analysis^35–37^.

To the best of our knowledge, no distribution studies have been conducted in various rat tissues using MALDI Orbitrap MS based on the detection and characterization of SV and its metabolites in selected biological samples. In this study, SV was administered to a group of rats, and the detection of SV and its metabolites in selected organs/tissues (lung, liver, brain, heart and kidney) and biological samples (blood, urine and feces) was studied in vivo. Two cell disruption methods, namely, pulverization and homogenization, were applied to extract SV and its metabolites from the organs/tissues, and MALDI Orbitrap MS was used to identify the distribution of SV and metabolites in organs. In addition to identifying SV and its metabolites using MALDI Orbitrap MS, MS scans on unit resolution ion trap and high resolution Orbitrap MS platforms were carried out on all rat organ samples to compare the detection sensitivity and accuracy of the two platforms.

## Experimental section

### Animal, materials and reagents

Sprague–Dawley rats were obtained from the animal facility of the College of Pharmacy. All the animal experiments were performed following the standards set forth in the experimental animal use and care guidelines of the National Institute of Health and the supervision of animal experiments committee. The study was validated and approved by the committee for animal ethics of the King Saud University (No. KSU-SE-19-73). The study was carried out in accordance with ARRIVE guidelines. SV was obtained from Sigma-Aldrich, Kenilworth, NJ, USA. Methylcellulose (viscosity: 400 cP) was obtained from Sigma-Aldrich, Saint Louis, MO, USA, distilled/deionized water (Milli-Q Advantage A10) from Merck Millipore Frankfurter Strasse 250 Darmstadt Germany, rotor stator homogenizer (Sirial Number: SNTH21832; 5000–35,000 RPM) from Omni International, Kennesaw, GA, USA, liquid nitrogen (Air Liquid, Riyadh, SA), methanol (purity HPLC-99.9%) from Sigma-Aldrich, Saint Louis, MO, USA, metabolic cages, oral gavage tubes, syringes, surgical tools, mortal-pestle, beakers/glass tubes/glass vials and other equipment (i.e., − 80 °C fridge, balance, centrifuge, nitrogen stream evaporator, etc.) were obtained from the lab facilities at the College of Pharmacy.

### In vivo experimental procedure

Six rats were randomized into two groups, with 3 in each group (1 for the control). Simvastatin was administered orally via gastric gavage at a dose of 100 mg/kg of body weight (diluted in 0.5% methylcellulose). The physiological saline solution was orally administered to the control group (average weight 205 ± 15 g). Animals were placed in metabolic cages for the collection of urine and feces. Rats were sacrificed through cervical dislocation 1 h after drug administration, and blood and tissue samples (brain, heart, kidney, lung and liver) were collected. Tissues collected from one group of rats were disrupted via homogenization by applying shearing force, while tissues from the other group were disrupted through pulverization (grinding). The disrupted cells were then extracted, centrifuged and reconstituted before being directed for mass data analysis using MALDI Orbitrap MS.

### Rat models

#### Preparation of the SV vehicle for oral administration in rats

The vehicle for SV was water + 0.9% NaCl + 0.5% methylcellulose. The SV vehicle (viscous solution) was prepared by boiling 100 mL of distilled water in a 500 mL conical flask. Next, 1 g of methylcellulose was added slowly over approximately 2–3 min with swirling. The mixture was cooled to 30–40 °C with stirring, and then 100 mL of a freshly prepared 1.8% NaCl solution were added and stirred overnight at 4 °C.

#### Oral gavage and biological sample collection (group I)

Label, weigh and dose the rats (in duplicate): The tails of rats were labeled with different colors of dyes. The rats were weighed separately, and the readings were recorded (Table S-1). According to the weight of each rat, the amount of simvastatin (100 mg/kg) to be dissolved in recommended volumes of the vehicle (water + 0.9% NaCl + 0.5% methylcellulose; 5 mL/kg was used) was calculated based on “Oral Dosing (Gavage) in Adult Mice and Rats SOP”, “Recommended Dose Volumes for Common Laboratory Animals” & “Administration of Substances to Laboratory Animals: Routes of Administration and Factors to Consider”^38^. The rats were dosed separately and placed in metabolic cages at 30 min intervals to ensure sufficient time for sacrifice and the collection of biological samples from individual rats 1 h after drug administration (Table S-2). Blood, urine, tissue samples (brain, heart, kidney, lung and liver) and the content of intestine (feces) were collected and placed at − 80 °C before further sample preparation, except blood, which was placed at 4 °C.

#### Oral gavage and biological sample collection (group II)

Label, weigh and dose the rats (in duplicate): The same processes were performed for Group II as for Group I (Tables S-3, S-4).

### Biological sample preparation using different cell disruption methods

#### Homogenization of collected biological samples from rats in group I

The collected blood was allowed to clot by incubating it undisturbed at room temperature for 30 min. The clot was removed by centrifugation at 1000–2000 × g for 10 min in a refrigerated centrifuge. The supernatant (serum) was transferred to a clean Eppendorf tube using a Pasteur pipette. Tissue samples (brain, heart, kidney, lung and liver) were removed from the − 80 °C freezer and defrosted. The organs were cut into small pieces and divided into different glass tubes (to ensure better homogenization). The extraction of SV and its metabolites from each tissue was performed by adding 1–2 mL of a mixture of methanol–water (9:1, v/v), depending on the amount of the tissue in each glass tube (plastic tubes were avoided due to the use of an extraction solvent). Further extraction was performed by homogenizing all samples using a rotor–stator homogenizer at 25,000 rpm for 20 s. Supernatants were combined (combined extracts are from different portions of the same organ from the same organism) and collected after centrifugation at 3,000 × g for 5 min and stored at 4 °C. Four milliliters of the abovementioned mixture of methanol–water (9:1, v/v) were added to 202 mg of rat feces, and 4 mL of pure methanol were added to 1 mL of serum and 0.5 mL of urine to extract SV and its metabolites (for each rat). All samples were further homogenized separately and centrifuged at 3000 × g for 5 min. Supernatants were evaporated to dryness using a nitrogen stream and dissolved in 300 μl of a mixture of acetonitrile–water (50:50, v/v). Samples of rat feces were diluted 1000 × before analysis.

#### Cryogenic grinding/pulverization of collected organs from group II rats

Tissue samples (brain, heart, kidney, lung and liver) were removed from the − 80 °C freezer prior to the pulverization of each sample. Liquid nitrogen was poured into a mortar to precool the mortar-pestle set. The sample previously frozen at − 80 °C was rendered brittle with liquid nitrogen before or during grinding. Frozen tissues were ground to a homogenous powder. Each pulverized tissue sample was transferred to a 15 mL Falcon tube containing 4–5 mL of the extraction solvent composed of methanol–water (9:1, v/v), depending on the amount of the powder in each tube, and mixed well. Samples were then centrifuged at 16,000 × g for 10 min. Supernatants were collected and stored at 4 °C. The sample preparation method for biological fluids and feces was the same as the procedure used for Group I.

### Mass spectrometry (MS) analysis

#### MALDI-LTQ Orbitrap MS

Mass spectra were measured using an ultrahigh-resolution MALDI-LTQ Orbitrap MS platform (Thermo Fisher Scientific, Waltham, MA, USA) equipped with a nitrogen UV laser (337 nm, 60 Hz) with a beam diameter of approximately 80 μm × 100 μm. The LTQ Orbitrap instrument was operated in both positive-ion and negative-ion modes over a normal mass range (*m/z* 100–1000). Tuning parameters were optimized individually for the matrices used in the present study. The number of laser shots and power were determined based on tests performed with automatic gain control on a small area of the tested tissue slide (the target value was set to 5.105). The parent compound SV was used to determine the parent spectrum that serves as a control spectrum for tuning the MS conditions. Optimized MS parameters for SV were as follows: Analysis was performed in FTMS with positive/negative mode, ASF/AGC was kept on; plate motion was set to survey CPS mode; laser energy was applied 5–25 µJ; and the scan range was set 100–1000 m*/z*. Data were analyzed using Thermo Xcalibar Qual Browser software, Version 3.1.

#### Ion trap LC/MS

LC–MS/MS measurements were performed using a model 6320 Ion Trap (Agilent Technologies, USA) equipped with an electrospray ionization source (ESI). Electrospray ionization was performed at room temperature in positive/negative mode. The voltage was maintained at 4.5 kV, the nebulizer pressure was 60 psi, the dry gas was 12 l/m, the dry temperature was 350 °C, the trap drive level was 100% and the capillary temperature was 325 °C. The scan range was set from 100 to 1000 Daltons. The column used was an Eclipse plus C18 (150 × 4.6 mm, 5 micron). LC separation was carried out using a mobile phase composed of ammonium acetate buffer (pH = 4) in water (solvent A) and acetonitrile (v/v) (solvent B). Gradient chromatography (run of 30 min) was performed with ammonium acetate buffer (pH = 4) in a water/acetonitrile mixture as the mobile phase at a flow rate of 0.3 mL/min. The program started with 70% mobile phase A, and then the amount of mobile phase B was increased from 30 to 70% within 30 min. Vials containing samples to be analyzed using HPLC were placed in the autosampler integrated in the HPLC machine, and 1 µl of a particular sample was injected into the HPLC system connected to the Agilent Ion Trap.

#### MALDI matrix selection and optimization

The optimization of the various matrices, 2,5-DHB, 1,5-DAN, 9-AA and CHCA, selected for MALDI was performed with both SV and the extract of rat liver tissues (where most SV metabolites were expected to be detected). All four matrix (2,5-DHB, 1,5-DAN, 9-AA and CHCA) solutions were prepared by dissolving each in H_2_O/ACN (v/v, 50:50). 2,5-DHB (10 mg), 1,5-DAN (1 mg), 9-AA (1 mg), CHCA (10 mg) were separately dissolved in 500 µL (H_2_O/CAN), respectively, for obtaining saturated supernatant. The solutions were centrifuged to collect the supernatant and obtain saturated matrices. The selection of suitable matrices was performed based on the most intense MS signal obtained from the analysis of SV and liver extracts.

#### Methods for depositing samples onto the MALDI plate

The suitability of different sample loading methods onto MALDI plate, namely, the dried droplet method (mixing matrices and the sample together in an Eppendorf tube, pouring the sample into a 96-well plated MALDI plate, and then co-crystallization of the sample and matrix during evaporation of the solvent), on plate mixing method (matrices were poured into a 96-well plated MALDI plate and the sample was then added before the matrix dried and was mixed using pipet; the mixture was then co-crystalized during the evaporation of the solvent) and sandwich method (matrices were poured into a 96-well MALDI plate, dried, the sample was then added on top of the dry matrix, and another layer of each matrix was added), was compared for the standard SV and extracts of tissue samples (i.e., liver) to determine the method that achieved the most intense signal for SV in both positive- and negative-ion modes. Micromolar solution of SV and in vivo reconstituted (in 1 mL) sample was used for analysis. Sample *vs* matrix were used at the ratios of 1:1, 1:5, 1:10, 1:25 and 1:50 with three analytical replicates.

## Results and discussion

The distribution of SV and its metabolites was studied in two groups of three Sprague–Dawley rats (weighing 400 g). SV (100 mg/kg) was administered orally, and urine and feces were collected. Rats were sacrificed through cervical dislocation 1 h after dosing, and serum and selected tissues (liver, heart, brain, lung and kidney) were collected. Tissues collected from the two groups were either subjected to homogenization by applying shearing force or pulverization/grinding before extraction, centrifugation and reconstitution. Samples obtained from biological samples (urine, serum and feces) and selected tissue homogenates/pulverized samples were filtered, evaporated and reconstituted with ACN-water (50:50, v/v) before being subjected to mass data analysis with a MALDI-LTQ Orbitrap MS in positive/negative (+/−) mode. The prepared samples were analyzed using MALDI Orbitrap MS. Optimization of the four tested matrices (DHB, DAN, 9-AA and CHCA) resulted in the selection of DHB and DAN for positive and negative modes, respectively (Table 1).

**Table 1:** MALDI matrix selection and laser energy (µJ) optimization.

	Matrices		Best ionization	Laser energy 5–25 (µJ)
Positive mode			DHB	10
Negative mode			DAN	6

**Figure 2:**
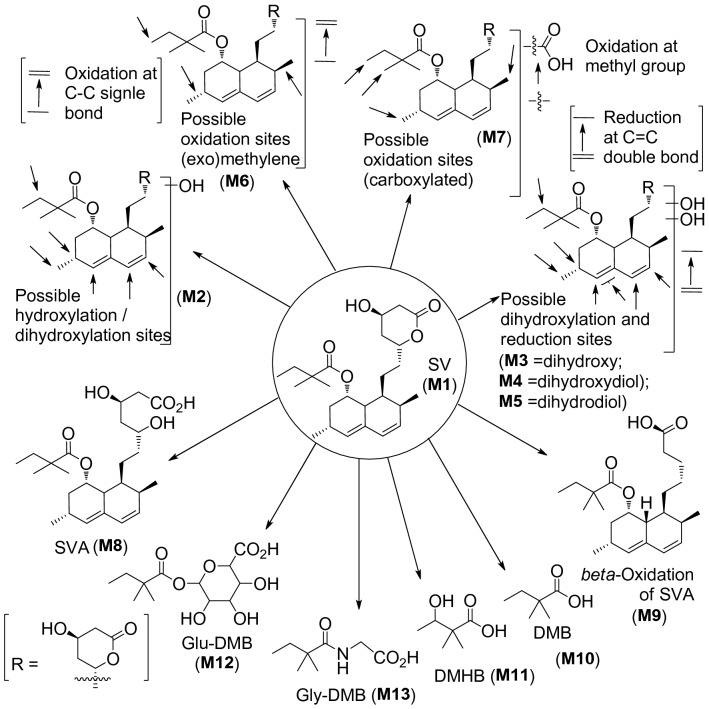
The in vivo metabolism of SV.

Among the three sample deposition methods, namely, dried droplet, plate mixing and sandwich, the best results were obtained using the dried droplet sample deposition method; therefore, this method was chosen for the analysis of all samples. The settings of a mass resolution of 100,000 and mass accuracy of 5 ppm enabled the SV-related ions to be distinguished from ions related to other components of the isobaric matrix and endogenous compounds. The in vivo metabolism of SV is shown in Fig. 2.

Table 2 shows metabolites detected in MALDI Orbitrap MS from the extracts of biological fluids in positive/negative modes. Seven different metabolites, SV (**M1**), hydroxy-SV (SV-OH) (**M2**), dihydroxy-SV (**M3**), dihydrodiol-SV (**M4**), reduced-SV (**M5**), exomethylene-SV (**M6**) and carboxyl-SV (**M7**), were detected in positive mode. On the other hand, six different metabolites, SVA (**M8**), *β*-oxidation of SVA (**M9**), 2,2-dimethybutyric acid (DMB) (**M10**), 2,2-dimethyl-3-hydroxybutyric acid (DMHB) (**M11**), 6-(2,2-dimethylbutanoyl)oxy)-3,4,5-trihydroxytetrahydro-2H-pyran-2-carboxylic acid (glu-DMB) (**M12**) and 2-(2,2-dimethylbutanamido)acetic acid (gly-DMB) (**M13**), were identified in negative mode (Table 2).

**Table 2:** Metabolites identified in feces and biological fluids (positive/negative mode).

Samples	Detected metabolites (mode: +/−)		Molecular formula [M + H]^+^/[M − H]^−^	Exact mass (monoisotopic) [M + H]^+^/[M − H]^−^	Accurate mass [M + H]^+^/[M − H]^−^	Mass accuracy Δ (< 5 ppm)
Feces	**M1**	(Positive ‘+’)	C_25_H_39_O_5_	419.27920	419.27902	− 0.4
	**M2**		C_25_H_39_O_6_	435.27412	435.27414	0.1
	**M3**		C_25_H_39_O_7_	451.26903	451.26825	− 1.7
	**M4**		C_25_H_41_O_7_	453.28468	453.28452	− 0.4
	**M5**		C_25_H_41_O_5_	421.29485	421.29367	− 2.8
	**M6**		C_25_H_37_O_5_	417.26355	417.26349	− 0.1
	**M7**		C_25_H_37_O_7_	449.25338	449.25330	− 0.2
	**M8**	(Negative ‘−’)	C_25_H_39_O_6_	435.27412	435.27393	− 0.4
	**M9**		C_23_H_35_O_4_	375.25299	375.25360	1.6
Urine	**M1**	(Positive ‘+’)	C_25_H_39_O_5_	419.27920	419.27887	− 0.8
	**M2**		C_25_H_39_O_6_	435.27412	435.27371	− 0.9
	**M3**		C_25_H_39_O_7_	451.26903	451.26740	− 3.6
	**M4**		C_25_H_41_O_7_	453.28468	453.28564	2.1
	**M5**		C_25_H_41_O_5_	421.29485	421.29459	− 0.6
	**M6**		C_25_H_37_O_5_	417.26355	417.26334	− 0.5
	**M7**		C_25_H_37_O_7_	449.25338	449.25351	0.3
	**M11**	(Negative ‘−’)	C_6_H_11_O_3_	131.07027	131.07019	− 0.6
	**M12**		C_12_H_19_O_8_	291.10744	291.10806	2.1
	**M13**		C_8_H_14_O_3_N	172.09682	172.09688	0.3
Serum	**M1**	(Positive ‘+’)	C_25_H_39_O_5_	419.27920	419.27921	0.0
	**M2**		C_25_H_39_O_6_	435.27412	435.27356	− 1.3
	**M3**		C_25_H_39_O_7_	451.26903	451.26889	− 0.3
	**M4**		C_25_H_41_O_7_	453.28468	453.28476	0.2
	**M5**		C_25_H_41_O_5_	421.29485	421.29514	0.7
	**M6**		C_25_H_37_O_5_	417.26355	417.26370	0.4
	**M7**		C_25_H_37_O_7_	449.25338	449.25220	− 2.6
	**M8**	(Negative ‘−’)	C_25_H_49_O_6_	435.27412	435.27438	0.6
	**M10**		C_6_H_11_O_2_	115.07536	115.07547	1.0
	**M11**		C_6_H_11_O_3_	131.07027	131.07022	− 0.4

Metabolites identified in selected organ extracts from group I (homogenized samples) and group II (pulverized samples) using MALDI Orbitrap MS in positive/negative mode are summarized in Table 3 as well as in Table S-1. Notably, all twelve metabolites, except the *β*-oxidation product of SVA (-) (**M9**), were identified in both homogenized and pulverized samples. The comparison of the two different cell disruption methods mentioned above indicated that sample preparation through pulverization generally produced better identification using MALDI-LTQ Orbitrap MS, except for the liver sample detected in positive mode, whereas homogenization seemingly resulted in a larger number of metabolites identified. This result was not surprising, as the pulverized samples have a larger surface area, which provides better contact during extraction; thus, more metabolites were extracted, leading to the identification of a greater number of metabolites overall. However, the *β*-oxidized SVA (**M9**) metabolite was not observed in any organs or fluids except feces. Among the metabolites detected, reduced-SV (**M5**) is being reported for the first time. No SV or metabolites were detected in rat brain tissue samples, potentially because metabolites tend to be too hydrophilic to cross the blood–brain barrier. Additionally, the rat brain was collected 1 h after the oral administration of a single dose, and trace amounts of SV and its metabolites potentially resulting from extensive first metabolism might have been cleared before they even reached the brain tissue. The mass accuracy for identification of all the metabolites was set to < 5 ppm. Most of the detected metabolites have a mass accuracy of < 3 ppm, the majority of which have a sub ppm (< 1 ppm) range. As shown in sample ‘G2-Bio-positive’ in the supporting data (Figs. S-1, S-2, S-3, S-4 and S-5) for different metabolites **M1**, **M3**, **M4**, **M5** and **M6** (Table 2; entry feces), even in different runs, the maximum mass error was − 2.6 ppm. In brief, the mass accuracy of SV shows − 0.4 (Table 2; entry ‘feces’ metabolites **M1**); reduced-SV shows − 2.8 (Table 2; entry ‘feces’ metabolites **M5**), dihydroxy-SV shows − 1.7 (Table 2; entry ‘feces’ metabolites **M3**), dihydrodiol-SV shows − 0.4 (Table 2; entry ‘feces’ metabolites **M4**) and exomethylene-SV shows − 0.1 (Table 2; entry ‘feces’ metabolites **M6**) ppm, respectively, therefore, the maximum mass error was found − 2.7 (− 2.8 − {− 0.1}) ppm, which all fall within the 5 ppm range we set as cutoff for all the data.

**Table 3:** Metabolites identified in selected homogenized/pulverized organs in positive/negative mode.

Selected organs	Detected metabolites (mode: +/−)		Molecular formula [M + H]^+^/[M − H]^−^	Exact mass (monoisotopic) [M + H]^+^/[M − H]^−^	Accurate mass [M + H]^+^/[M − H]^−^	Mass accuracy Δ (< 5 ppm)	Homogenated	Pulverized
Liver	**M1**	(Positive ‘+’)	C_25_H_39_O_5_	419.27920	419.27832	− 2.1	√	
	**M2**		C_25_H_39_O_6_	435.27412	435.27374	− 0.9	√	√
	**M5**		C_25_H_41_O_5_	421.29485	421.29620	3.3	√	√
	**M6**		C_25_H_37_O_5_	417.26355	417.26309	− 1.1	√	
	**M7**		C_25_H_37_O_7_	449.25338	449.25165	− 3.9	√	
	**M8**	(Negative ‘−’)	C_25_H_39_O_6_	435.27412	435.27399	− 0.3	√	√
Heart	**M1**	(Positive ‘+’)	C_25_H_39_O_5_	419.27920	419.27914	− 0.1	√	√
	**M2**		C_25_H_39_O_6_	435.27412	435.27490	1.8	–	√
	**M3**		C_25_H_39_O_7_	451.26903	451.26822	− 1.8	√	√
	**M4**		C_25_H_41_O_7_	453.28468	453.28357	− 2.4	√	√
	**M5**		C_25_H_41_O_5_	421.29485	421.29620	3.2	√	√
	**M6**		C_25_H_37_O_5_	417.26355	417.26303	− 1.2	√	√
	**M7**		C_25_H_37_O_7_	449.25338	449.25409	1.6	–	√
	**M8**	(Negative ‘−’)	C_25_H_49_O_6_	435.27412	435.27542	3.0	√	√
	**M10**		C_6_H_11_O_2_	115.07536	115.07554	1.6	√	√
	**M11**		C_6_H_11_O_3_	131.07027	131.06995	− 2.5	–	√
Kidney	**M2**	(Positive ‘+’)	C_25_H_39_O_6_	435.27412	435.27509	2.2	–	√
	**M3**		C_25_H_39_O_7_	451.26903	451.26883	− 0.4	–	√
	**M4**		C_25_H_41_O_7_	453.28468	453.28448	− 0.4	–	√
	**M5**		C_25_H_41_O_5_	421.29485	421.29633	3.5	–	√
	**M6**		C_25_H_37_O_5_	417.26355	417.26422	1.6	√	–
	**M8**	(Negative ‘−’)	C_25_H_49_O_6_	435.27412	435.27487	1.7	√	√
	**M11**		C_6_H_11_O_3_	131.07027	131.06987	− 3.0	√	√
	**M12**		C_12_H_19_O_8_	291.10744	291.10712	− 1.1	√	√
	**M13**		C_8_H_14_O_3_N	172.09682	172.09677	− 0.3	√	√
Lung	**M1**	(Positive ‘+’)	C_25_H_39_O_5_	419.27920	419.27899	− 0.5	√	√
	**M2**		C_25_H_39_O_6_	435.27412	435.27206	− 4.7	–	√
	**M3**		C_25_H_39_O_7_	451.26903	451.26965	1.4	–	√
	**M4**		C_25_H_41_O_7_	453.28468	453.28470	0.0	–	√
	**M5**		C_25_H_41_O_5_	421.29485	421.29565	1.9	–	√
	**M6**		C_25_H_37_O_5_	417.26355	417.26239	− 2.8	√	–
	**M7**		C_25_H_37_O_7_	449.25338	449.25153	− 4.1	–	√
	**M8**	(Negative ‘−’)	C_25_H_49_O_6_	435.27412	435.27328	− 1.9	√	√
	**M10**		C_6_H_11_O_2_	115.07536	115.07519	− 1.5	√	√
	**M11**		C_6_H_11_O_3_	131.07027	131.07002	− 1.9	√	√

The distribution of SV and its metabolites in various tissues, serum, urine and feces is shown in Table 4. In this study, two groups of rats each with two test animals and one control animal, making the total number of animals six, were used. In consequence, 5 organ samples (Liver, heart, lung, kidney, brain; both homogenized and pulverized) and 3 biological fluid samples (serum, urine, and feces) were obtained from each animal making the total 78 samples that were ultimately analyzed both in the positive and negative modes, giving 156 runs with triplicates around, 468 runs. All thirteen metabolites were identified using MALDI in almost all samples evaluated. It should be noted that, each organ was having 8 samples (homogenized and pulverized) and each biological fluid was having 4 samples, excluding controls. In case of identified metabolites, we could detect in every samples either in homogenized and/or pulverized, in positive or negative modes and in triplicates runs, and therefore, the metabolites are reproducible. MALDI mass spectra are provided in the supporting information file (Figs. S-1 to S-13). In addition, all samples were analyzed using ion trap LC–MS for the comparison study, and their mass spectra are also shown in the supporting information (Figs. S-14 to S-40). Notably, 8 of the 13 metabolites were detected using conventional ion trap LC–MS. Briefly, **M8** was detected in feces; SV (**M1)**, **M2**, **M3** and **M7** were detected in urine; SV (**M1)**, **M4** and **M5** were detected in serum; SV (**M1)**, **M2**, **M3**, **M4**, **M7** and **M8** were detected in lung; SV (**M1)**, **M2**, **M8** and *β*-oxidized SVA (**M9**) were detected in liver; SV (**M1)**, **M2**, **M3**, **M4** and **M8** were detected in heart; and **M2** and **M4** in were detected kidney using ion trap LC/MS. The structures of those identified metabolite were elucidated using LC–MS/MS fragmentation pattern and have compared with the fragmentation pattern of SV. Proposed fragmentation pattern was inserted in the supporting information file (Figs. S-41 to S-46). However, the other 5 metabolites, **M6**, **M10**, **M11**, **M12** and **M13,** were not detected using ion trap LC/MS (please see the supporting information Table S-6 for the results of the comparison study). In conclusion, an in vivo distribution study revealed that MALDI orbitrap MS is a useful tool to study the distribution of a drug and its metabolites.

**Table 4:** List of metabolites of SV that were bio-transformed in vivo and identified using MALDI-LTQ Orbitrap MS and ion trap LC–MS with their possible metabolic reactions and the list of rat tissues of fluids where they are specifically distributed.

Identified metabolites	Possible metabolic reaction	Metabolites in rat tissues/fluids	
		Identified using Ion trap MS	Identified using MALDI
	Initial drug SV (**M1**)	Liver, heart, lung, serum, urine, and feces	Liver, heart, lung, serum, urine, and feces
	SV hydroxylation **M2**	Heart, lung, and urine	Liver, heart, lung, kidney, serum, urine, and feces
	Dihydroxylation of SV **M3**	Heart, lung, and urine	Heart, lung, kidney, serum, urine, and feces
	Reduction and dihydroxylation of SV **M4**	Heart, lung, and serum	Heart, lung, kidney, serum, urine, and feces
	SV reduction **M5**	None	Liver, heart, lung, kidney, serum, urine, and feces
	SV oxidation **M6**	None	Liver, heart, lung, kidney, serum, urine, and feces
	SV oxidation **M7**	Kidney, lung, and urine	Liver, heart, lung, serum, urine, and feces
	SV hydrolysis **M8**	Liver, lung, and feces	Liver, heart, lung, kidney, serum, and feces
	SVA *β*-oxidation **M9**	Liver	Feces
	Hydrolysis of SV at the 2,2-dimethylbutyryl moiety **M10**	None	Heart, lung, and serum
	Hydrolysis of SV-OH at 2,2-dimethylbutyryl moiety **M11**	None	Heart, lung, kidney, serum, and urine
	Glycine conjugate of DMB **M12**	None	Kidney and urine
	Glucuronidation of DMB **M13**	None	Kidney and urine

Mass spectra for all thirteen metabolites, including SV and blank bio-samples, are presented in the supporting information files (Figs. S-1 to S-13). For an example, spectra of the metabolites hydroxy-SV (**M2**), SVA (**M8**) and newly reported reduced-SV (**M5**) are presented below in Figs. 3, 4 and 5. The possible structures of the metabolites were confirmed using ion trap LC–MS/MS fragmentations, compared with parent simvastatin fragmentation pattern and are presented in the supporting information files.

**Figure 3:**
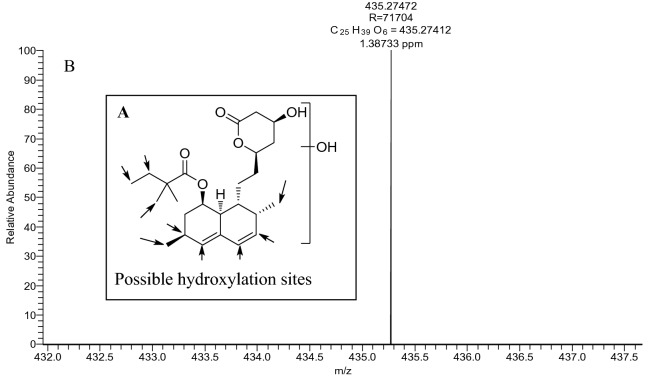
High-resolution mass spectrum of hydroxy-SV (**M2**) obtained using an Orbitrap MS showing possible hydroxylation sites of SV (**A**) and exact and accurate mass values (**B**).

**Figure 4:**
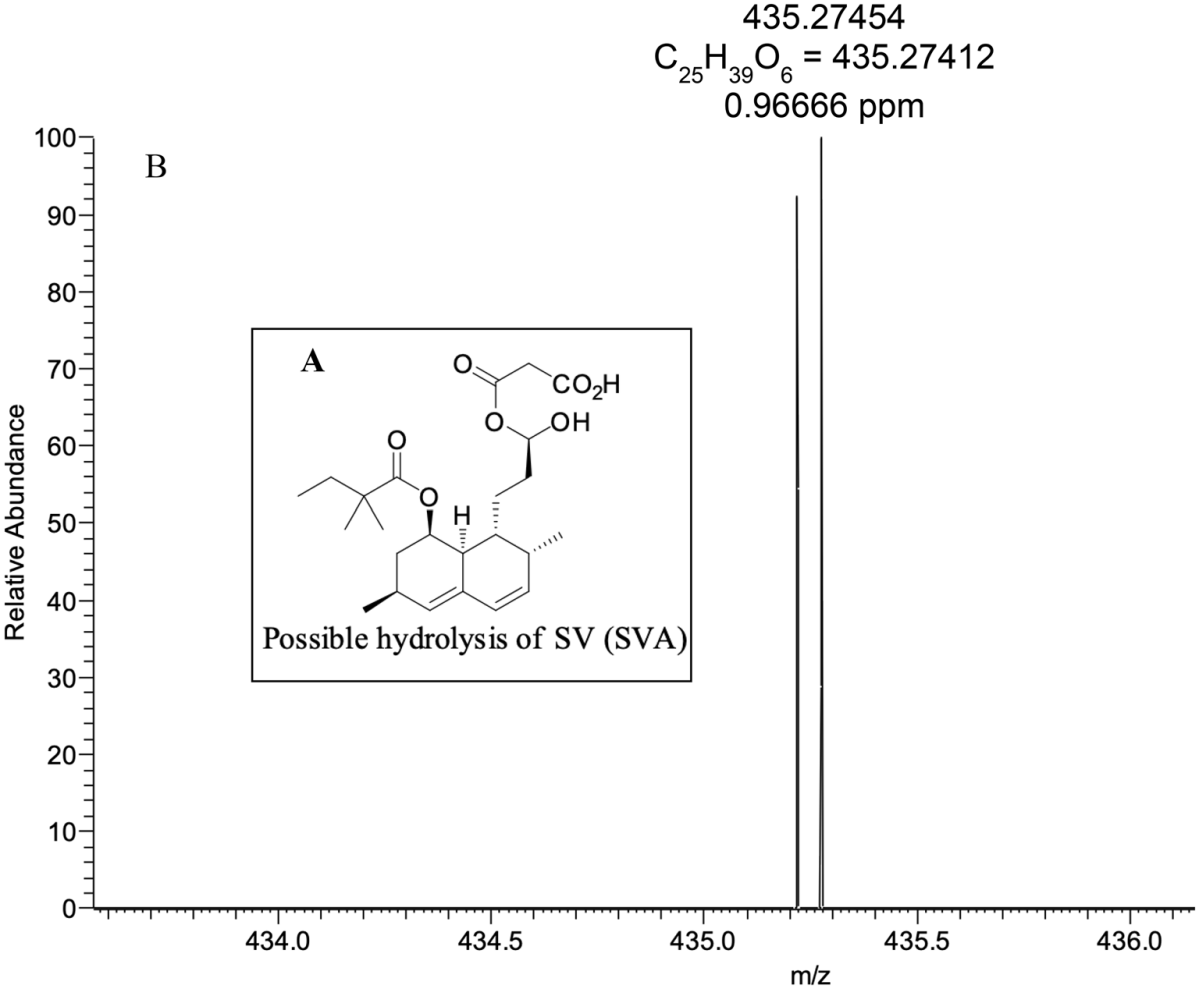
High-resolution mass spectrum of SVA (**M8**) obtained using an Orbitrap MS showing the possible hydrolysis of SV (**A**) and exact and accurate mass values (**B**).

**Figure 5:**
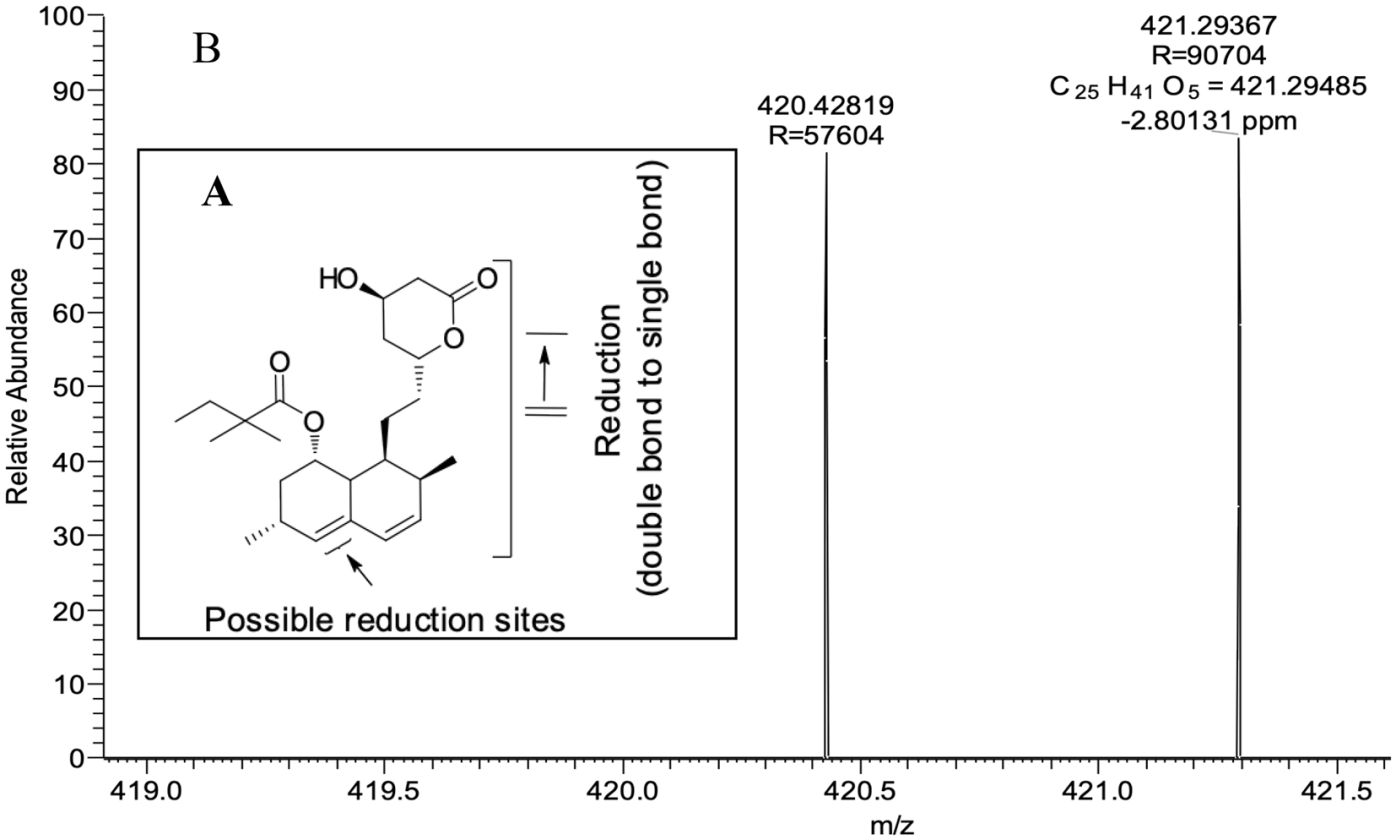
High-resolution mass spectrum of reduced-SV (**M5**) obtained using an Orbitrap MS showing the possible reduction of SV (**A**) and exact and accurate mass values (**B**).

Six possible hydroxylation sites of SV (Fig. 3A) where hydroxylation may occur have been identified. The exact mass of hydroxyl-SV (**M2**) detected in positive mode at *m/z* = 435.27412 [M + H]^+^ and the mass of the possible hydroxylated product are shown in the spectra at *m/z* = 435.27472 [M + H]^+^ with a mass accuracy of 1.4 ppm (Fig. 3B).

SVA (**M8**), a possible hydrolyzed reaction product, is observed in negative mode (Fig. 4) with an exact mass of *m/z* = 435.27412 [M − H]^−^, accurate mass of *m/z* = 435.27454 [M − H]^−^, and a mass accuracy of 1.0 ppm.

Reduced-SV (**M5**), a possible reductive product, is observed in positive mode (Fig. 5) with an exact mass of *m/z* = 421.29485 [M + H]^+^, and an accurate mass of *m/z* = 421.29367 [M + H]^+^, and a mass accuracy of − 2.8 ppm.

## Conclusions

The in vivo detection and characterization of SV and its metabolites was investigated in rat tissues (brain, liver, lung, heart and kidney) and collected biological samples (blood, urine and feces) and was studied using high-resolution MALDI Orbitrap MS. An evaluation of various matrices (DHB, DAN, 9-AA and CHCA) based on the most intense signal obtained from the HRMS analysis of SV and the liver extract indicated that DHB and DAN were the most suitable matrices for positive and negative modes, respectively. Several methods for depositing samples on MALDI plates, including dried droplets, on plate mixing and sandwich methods, were attempted, and the dried droplet method was the optimal method in our study. The distributions of SV and its metabolites based on the data analysis are summarized in a table. Among them, identified reduced-SV (**M5**) metabolite is reported for the first time. However, no metabolites were detected in the rat brain, which might be attributed to the collection of samples at only 1 h following the oral administration of a single dose of SV. Detection in the brain, however, can be achieved either by orally administering multiple doses of SV continuously for a few days or switching the oral delivery route to intravenous injection. The comparison of two different cell disruption methods for sample preparation revealed that pulverization generally generates better identification results using MALDI-LTQ Orbitrap MS, except for the liver sample detected in positive mode, where homogenization appeared to result in more detected metabolites. Overall, the pulverized samples were more uniform and larger in surface area, rendering their more efficient and complete extraction during sample preparation. As shown in the present study, MALDI Orbitrap MS is a useful tool to study drug metabolism and distribution.

## Supplementary Information

Supplementary Information.

## References

[1] Paul A, Raj GM, Raveendran R (2019). Drug distribution. Introduction to Basics of Pharmacology and Toxicology: Volume 1: General and Molecular Pharmacology: Principles of Drug Action.

[2] Marko-Varga G (2011). Drug localization in different lung cancer phenotypes by MALDI mass spectrometry imaging. J. Proteomics.

[3] Gillette JR (1973). The importance of tissue distribution in pharmacokinetics. J. Pharmacokinet. Biopharm..

[4] Lanao JM, Fraile MA (2005). Drug tissue distribution: Study methods and therapeutic implications. Curr. Pharm. Des..

[5] Baca QJ, Golan DE, Golan DE, Armstrong EJ, Armstrong AW (2017). Pharmaconinetics. Principles of Pharmacology: The pathophysiologic basis of drug therapy.

[6] Ersoy BA, Hoffmaster KA, Golan DE, Armstrong EJ, Armstrong AW (2017). Drug transporters. Principles of Pharmacology: The pathophysiologic basis of drug therapy.

[7] Guengerich FP, Golan DE, Armstrong EJ, Armstrong AW (2017). Drug transporters. Principles of Pharmacology: The pathophysiologic basis of drug therapy.

[8] Castellino S, Groseclose MR, Wagner D (2011). MALDI imaging mass spectrometry: Bridging biology and chemistry in drug development. Bioanalysis.

[9] Talreja O, Kerndt CC, Cassagnol M (2020). Simvastatin. StatPearls.

[10] Pawan Kumar B, Deepti J (2012). Simvastatin: Review of updates on recent trends in pharmacokinetics, pharmacodynamics, drug–drug interaction, impurities and analytical methods. Curr. Pharm. Anal..

[11] *Simvastatin*, https://web.archive.org/web/20150110101755/http://www.drugs.com/monograph/simvastatin.html (2015).

[12] Alberts AW (1990). Lovastatin and simvastatin–inhibitors of HMG CoA reductase and cholesterol biosynthesis. Cardiology.

[13] Novakova L (2009). Ultra high performance liquid chromatography tandem mass spectrometric detection in clinical analysis of simvastatin and atorvastatin. J. Chromatogr. B Anal. Technol. Biomed. Life Sci..

[14] Roche VF (2005). Antihyperlipidemic statins: a self-contained, clinically relevant medicinal chemistry lesson. Am. J. Pharm. Educ..

[15] Lennernäs H, Fager G (1997). Pharmacodynamics and pharmacokinetics of the HMG-CoA reductase inhibitors Similarities and differences. Clin. Pharmacokinet..

[16] Naci H, Brugts J, Ades T (2013). Comparative tolerability and harms of individual statins: a study-level network meta-analysis of 246 955 participants from 135 randomized, controlled trials. Circ. Cardiovasc. Qual. Outcomes.

[17] Di Stasi SL, MacLeod TD, Winters JD, Binder-Macleod SA (2010). Effects of statins on skeletal muscle: A perspective for physical therapists. Phys. Ther..

[18] Alkhatatbeh MJ, Abdul-Razzak KK, Khasawneh LQ, Saadeh NA (2018). Prevalence of musculoskeletal pain in association with serum 25-hydroxyvitamin D concentrations in patients with type 2 diabetes mellitus. Biomed. Rep..

[19] Weise WJ, Possidente CJ (2000). Fatal rhabdomyolysis associated with simvastatin in a renal transplant patient. Am. J. Med..

[20] Phillips PS, Haas RH, Bannykh S, Hathaway S, Gray NL, Kimura BJ, Vladutiu GD, England JDF (2002). Statin-associated myopathy with normal creatine kinase levels. Ann. Intern. Med..

[21] Meier CR, Schlienger RG, Kraenzlin ME, Schlegel B, Jick H (2000). HMG-CoA reductase inhibitors and the risk of fractures. JAMA.

[22] Pommier Y (2009). DNA topoisomerase I inhibitors: Chemistry, biology, and interfacial inhibition. Chem Rev.

[23] *Prescribing Medicines in Pregnancy Database*, https://www.huidziekten.nl/formularium/documenten/medicines-pregnancy.pdf (2014).

[24] Germershausen JI (1989). Tissue selectivity of the cholesterol-lowering agents lovastatin, simvastatin and pravastatin in rats in vivo. Biochem. Biophys. Res. Commun..

[25] Vickers S, Duncan CA, Chen IW, Rosegay A, Duggan DE (1990). Metabolic disposition studies on simvastatin, a cholesterol-lowering prodrug. Drug Metab. Dispos..

[26] Bonnel D (2011). MALDI imaging techniques dedicated to drug-distribution studies. Bioanalysis.

[27] Swales JG (2014). Mass spectrometry imaging of cassette-dosed drugs for higher throughput pharmacokinetic and biodistribution analysis. Anal. Chem..

[28] Drexler DM (2007). Utility of imaging mass spectrometry (IMS) by matrix-assisted laser desorption ionization (MALDI) on an ion trap mass spectrometer in the analysis of drugs and metabolites in biological tissues. J. Pharmacol. Toxicol. Methods.

[29] Drexler DM, Tannehill-Gregg SH, Wang L, Brock BJ (2011). Utility of quantitative whole-body autoradiography (QWBA) and imaging mass spectrometry (IMS) by matrix-assisted laser desorption/ionization (MALDI) in the assessment of ocular distribution of drugs. J. Pharmacol. Toxicol. Methods.

[30] Troendle FJ, Reddick CD, Yost RA (1999). Detection of pharmaceutical compounds in tissue by matrix-assisted laser desorption/ionization and laser desorption/chemical ionization tandem mass spectrometry with a quadrupole ion trap. J. Am. Soc. Mass Spectrom..

[31] Végvári Á (2015). Drug localizations in tissue by mass spectrometry imaging. Biomark. Med..

[32] Schwartz SA, Reyzer ML, Caprioli RM (2003). Direct tissue analysis using matrix-assisted laser desorption/ionization mass spectrometry: practical aspects of sample preparation. J. Mass Spectrom. JMS.

[33] Hsieh Y, Chen J, Korfmacher WA (2007). Mapping pharmaceuticals in tissues using MALDI imaging mass spectrometry. J. Pharmacol. Toxicol. Methods.

[34] Jirásko R, Holčapek M, Kuneš M, Svatoš A (2014). Distribution study of atorvastatin and its metabolites in rat tissues using combined information from UHPLC/MS and MALDI-Orbitrap-MS imaging. Anal. Bioanal. Chem..

[35] Alakhali KM (2013). Method validation for analysis of simvastatin in human plasma using liquid chromatography tandem mass spectrometry (LC-MS-MS). J. Clin. Diagn. Res..

[36] Uchiyama N (1991). Metabolic fate of 2,2-dimethylbutyryl moiety of simvastatin in rats: Identification of metabolites by gas chromatography/ mass spectrometry. Eur. J. Drug Metab. Pharmacokinet..

[37] Prueksaritanont T (1997). In vitro metabolism of simvastatin in humans [SBT]identification of metabolizing enzymes and effect of the drug on hepatic P450s. Drug Metab. Dispos..

[38] Turner PV, Brabb T, Pekow C, Vasbinder MA (2011). Administration of substances to laboratory animals: Routes of administration and factors to consider. J. Am. Assoc. Lab. Anim. Sci..

