# Supplementary material for: Detection and characterization of simvastatin and its metabolites in rat tissues and biological fluids using MALDI high resolution mass spectrometry approach
Source: Sci Rep. 2022 Mar 19;12:4757. doi: 10.1038/s41598-022-08804-x (PMC8934354; doi:10.1038/s41598-022-08804-x)
Supplement: Supplementary file 1 — Supplementary Information. [file 41598_2022_8804_MOESM1_ESM.pptx]

## Slide 1
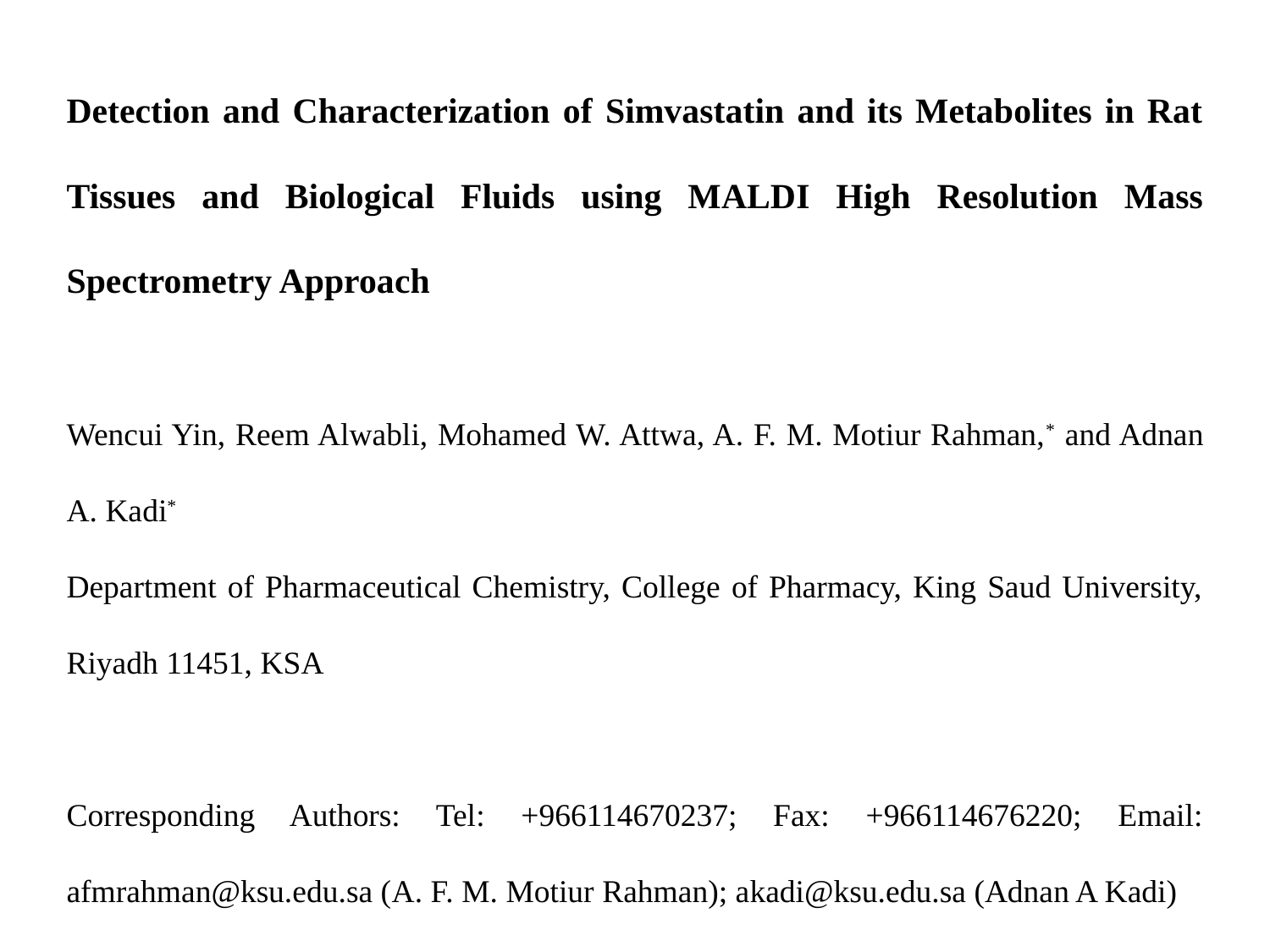

Detection and Characterization of Simvastatin and its Metabolites in Rat Tissues and Biological Fluids using MALDI High Resolution Mass Spectrometry Approach
Wencui Yin, Reem Alwabli, Mohamed W. Attwa, A. F. M. Motiur Rahman,* and Adnan A. Kadi*
Department of Pharmaceutical Chemistry, College of Pharmacy, King Saud University, Riyadh 11451, KSA
Corresponding Authors: Tel: +966114670237; Fax: +966114676220; Email: afmrahman@ksu.edu.sa (A. F. M. Motiur Rahman); akadi@ksu.edu.sa (Adnan A Kadi)

## Slide 2
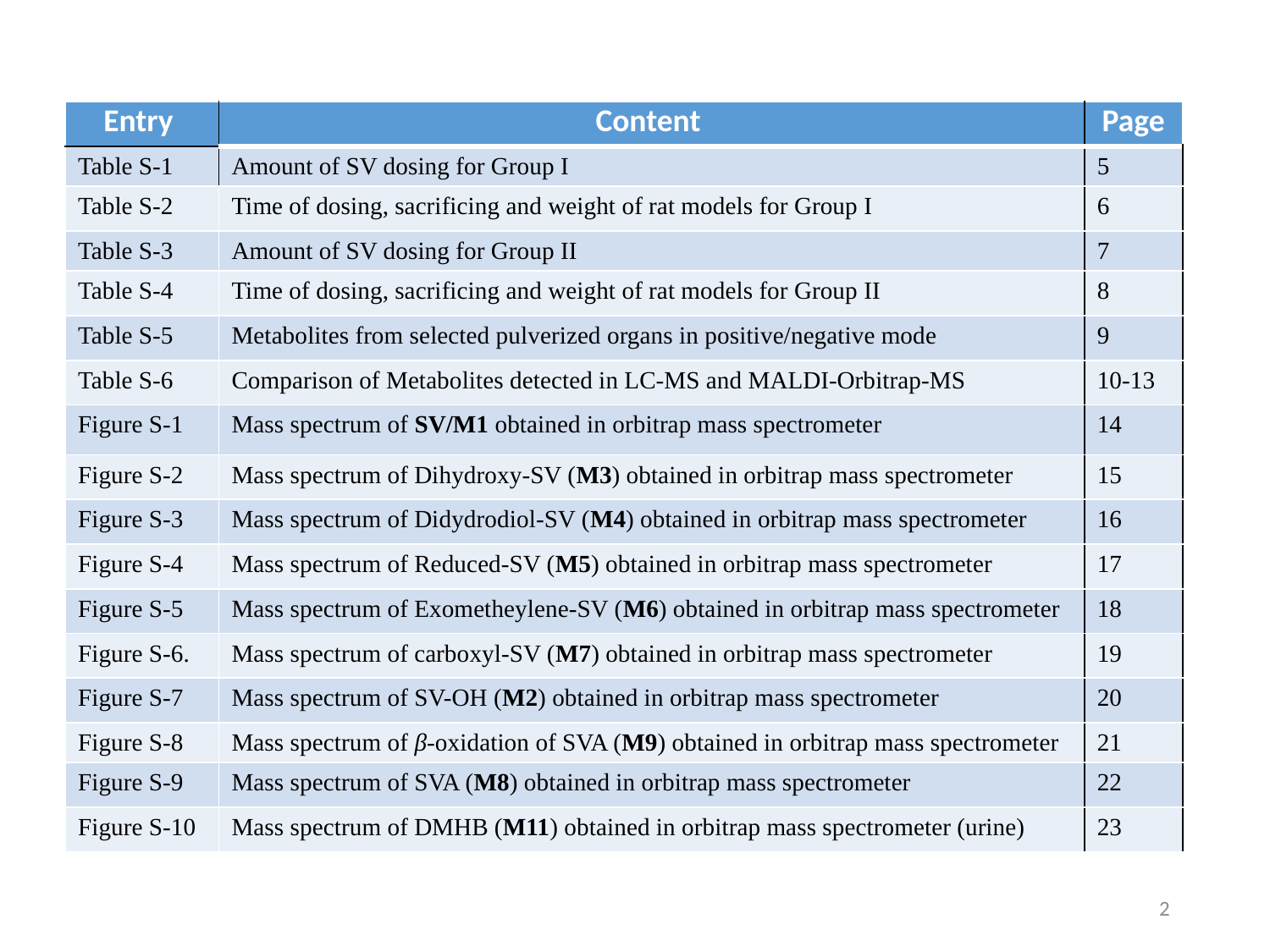

| Entry | Content | Page |
| --- | --- | --- |
| Table S-1 | Amount of SV dosing for Group I | 5 |
| Table S-2 | Time of dosing, sacrificing and weight of rat models for Group I | 6 |
| Table S-3 | Amount of SV dosing for Group II | 7 |
| Table S-4 | Time of dosing, sacrificing and weight of rat models for Group II | 8 |
| Table S-5 | Metabolites from selected pulverized organs in positive/negative mode | 9 |
| Table S-6 | Comparison of Metabolites detected in LC-MS and MALDI-Orbitrap-MS | 10-13 |
| Figure S-1 | Mass spectrum of SV/M1 obtained in orbitrap mass spectrometer | 14 |
| Figure S-2 | Mass spectrum of Dihydroxy-SV (M3) obtained in orbitrap mass spectrometer | 15 |
| Figure S-3 | Mass spectrum of Didydrodiol-SV (M4) obtained in orbitrap mass spectrometer | 16 |
| Figure S-4 | Mass spectrum of Reduced-SV (M5) obtained in orbitrap mass spectrometer | 17 |
| Figure S-5 | Mass spectrum of Exometheylene-SV (M6) obtained in orbitrap mass spectrometer | 18 |
| Figure S-6. | Mass spectrum of carboxyl-SV (M7) obtained in orbitrap mass spectrometer | 19 |
| Figure S-7 | Mass spectrum of SV-OH (M2) obtained in orbitrap mass spectrometer | 20 |
| Figure S-8 | Mass spectrum of β-oxidation of SVA (M9) obtained in orbitrap mass spectrometer | 21 |
| Figure S-9 | Mass spectrum of SVA (M8) obtained in orbitrap mass spectrometer | 22 |
| Figure S-10 | Mass spectrum of DMHB (M11) obtained in orbitrap mass spectrometer (urine) | 23 |
2

## Slide 3
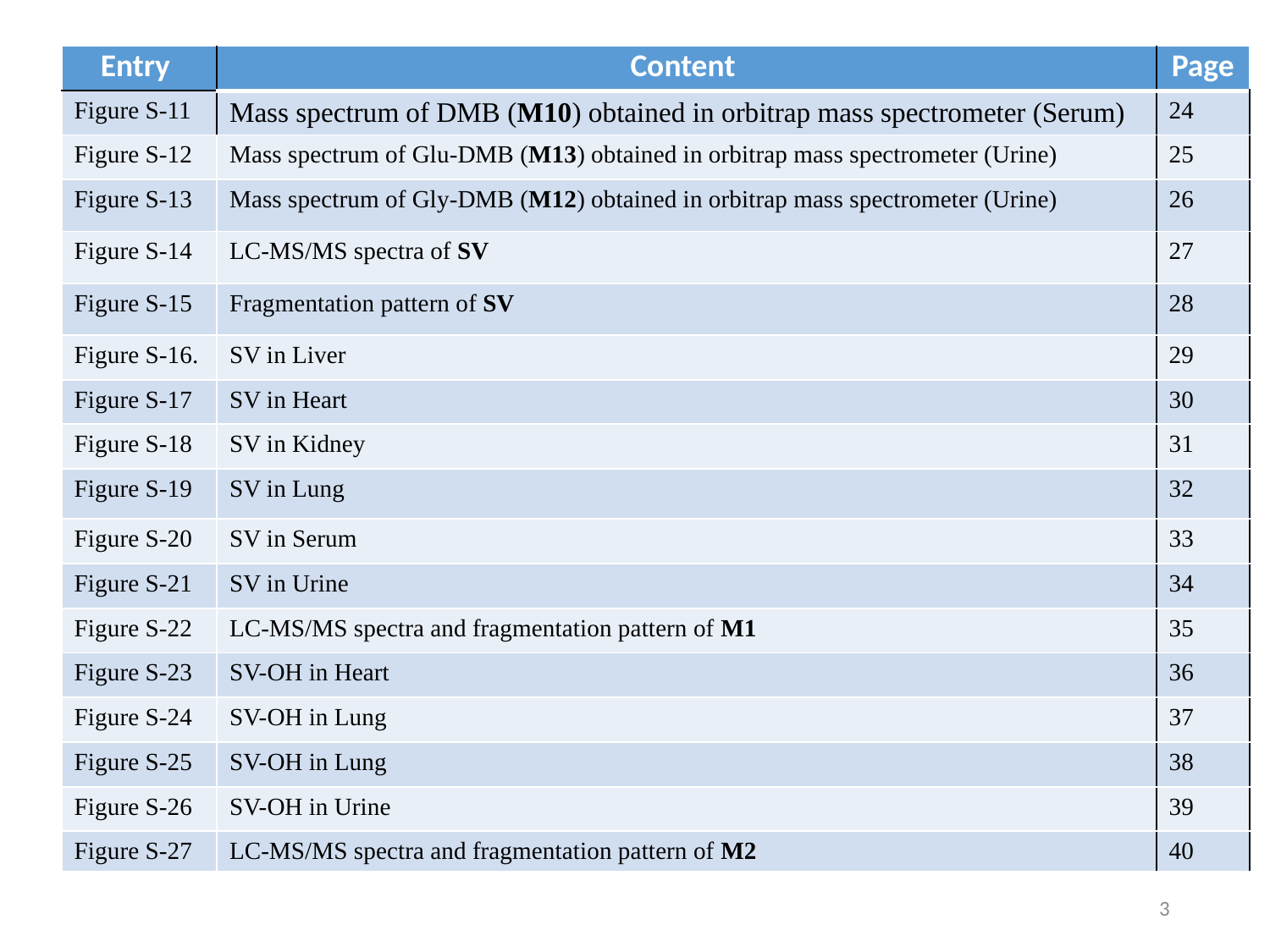

| Entry | Content | Page |
| --- | --- | --- |
| Figure S-11 | Mass spectrum of DMB (M10) obtained in orbitrap mass spectrometer (Serum) | 24 |
| Figure S-12 | Mass spectrum of Glu-DMB (M13) obtained in orbitrap mass spectrometer (Urine) | 25 |
| Figure S-13 | Mass spectrum of Gly-DMB (M12) obtained in orbitrap mass spectrometer (Urine) | 26 |
| Figure S-14 | LC-MS/MS spectra of SV | 27 |
| Figure S-15 | Fragmentation pattern of SV | 28 |
| Figure S-16. | SV in Liver | 29 |
| Figure S-17 | SV in Heart | 30 |
| Figure S-18 | SV in Kidney | 31 |
| Figure S-19 | SV in Lung | 32 |
| Figure S-20 | SV in Serum | 33 |
| Figure S-21 | SV in Urine | 34 |
| Figure S-22 | LC-MS/MS spectra and fragmentation pattern of M1 | 35 |
| Figure S-23 | SV-OH in Heart | 36 |
| Figure S-24 | SV-OH in Lung | 37 |
| Figure S-25 | SV-OH in Lung | 38 |
| Figure S-26 | SV-OH in Urine | 39 |
| Figure S-27 | LC-MS/MS spectra and fragmentation pattern of M2 | 40 |
3

## Slide 4
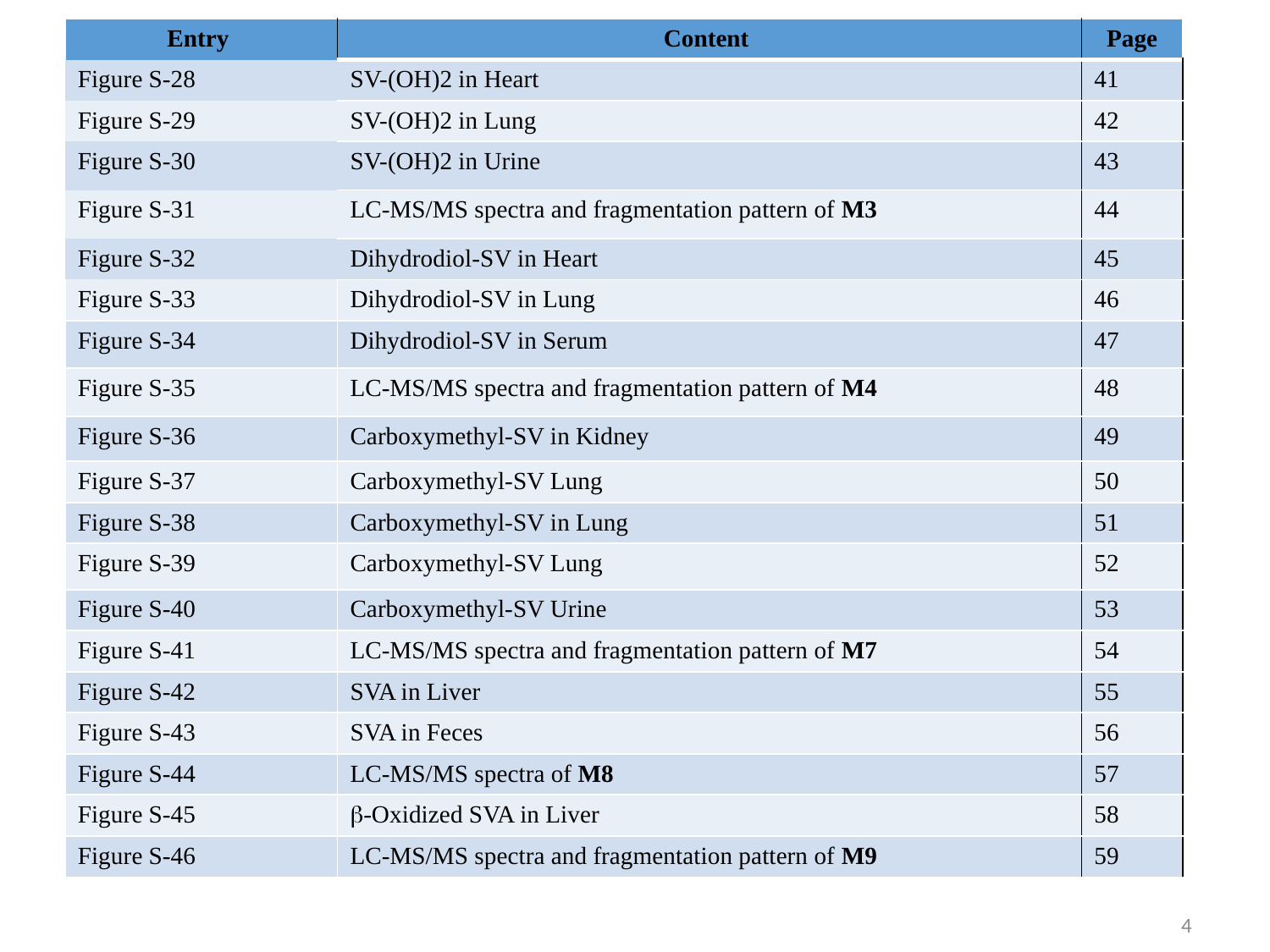

| Entry | Content | Page |
| --- | --- | --- |
| Figure S-28 | SV-(OH)2 in Heart | 41 |
| Figure S-29 | SV-(OH)2 in Lung | 42 |
| Figure S-30 | SV-(OH)2 in Urine | 43 |
| Figure S-31 | LC-MS/MS spectra and fragmentation pattern of M3 | 44 |
| Figure S-32 | Dihydrodiol-SV in Heart | 45 |
| Figure S-33 | Dihydrodiol-SV in Lung | 46 |
| Figure S-34 | Dihydrodiol-SV in Serum | 47 |
| Figure S-35 | LC-MS/MS spectra and fragmentation pattern of M4 | 48 |
| Figure S-36 | Carboxymethyl-SV in Kidney | 49 |
| Figure S-37 | Carboxymethyl-SV Lung | 50 |
| Figure S-38 | Carboxymethyl-SV in Lung | 51 |
| Figure S-39 | Carboxymethyl-SV Lung | 52 |
| Figure S-40 | Carboxymethyl-SV Urine | 53 |
| Figure S-41 | LC-MS/MS spectra and fragmentation pattern of M7 | 54 |
| Figure S-42 | SVA in Liver | 55 |
| Figure S-43 | SVA in Feces | 56 |
| Figure S-44 | LC-MS/MS spectra of M8 | 57 |
| Figure S-45 | β-Oxidized SVA in Liver | 58 |
| Figure S-46 | LC-MS/MS spectra and fragmentation pattern of M9 | 59 |
4

## Slide 5
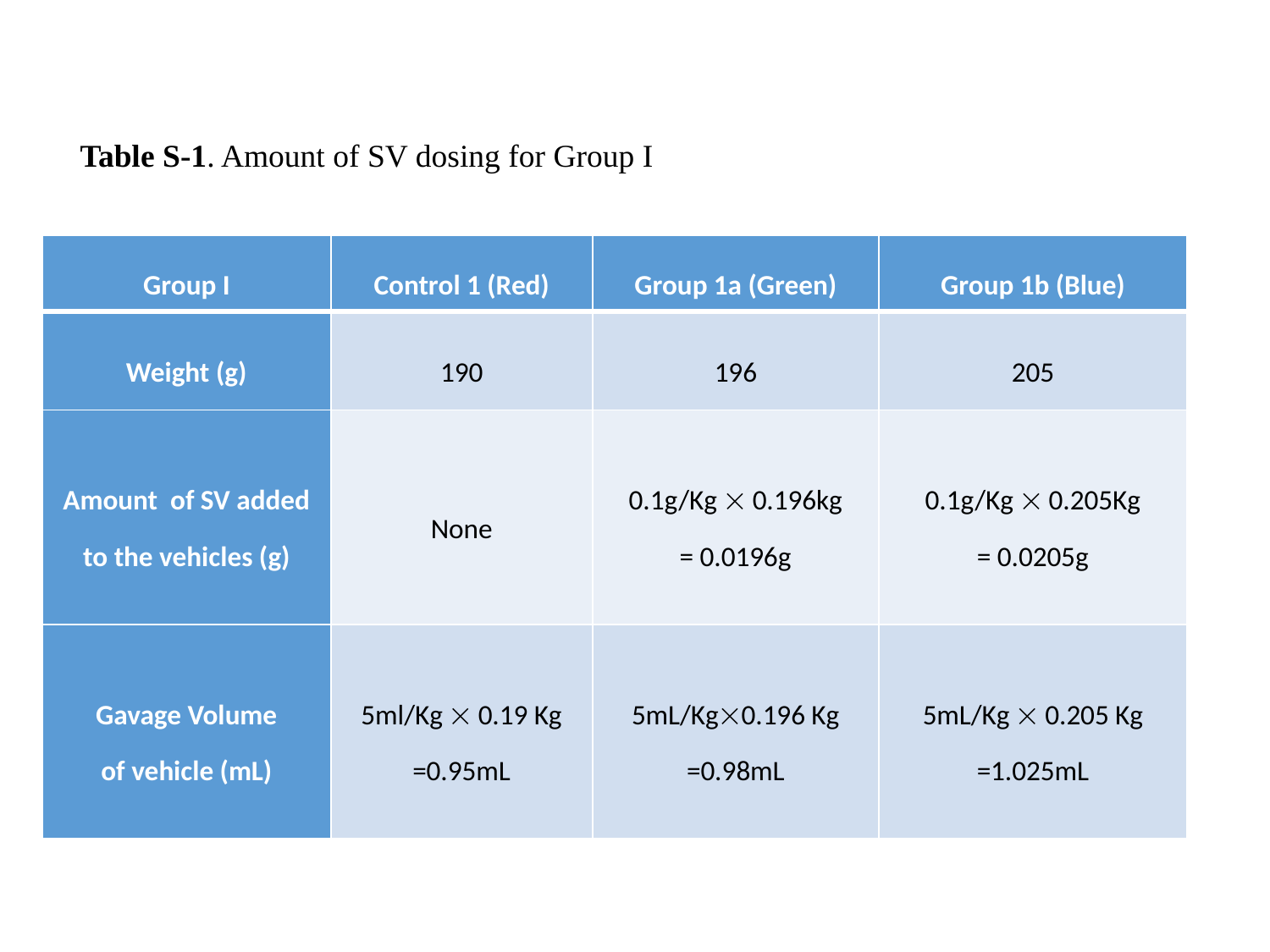

Table S-1. Amount of SV dosing for Group I
| Group I | Control 1 (Red) | Group 1a (Green) | Group 1b (Blue) |
| --- | --- | --- | --- |
| Weight (g) | 190 | 196 | 205 |
| Amount of SV added to the vehicles (g) | None | 0.1g/Kg × 0.196kg = 0.0196g | 0.1g/Kg × 0.205Kg = 0.0205g |
| Gavage Volume of vehicle (mL) | 5ml/Kg × 0.19 Kg =0.95mL | 5mL/Kg×0.196 Kg =0.98mL | 5mL/Kg × 0.205 Kg =1.025mL |

## Slide 6
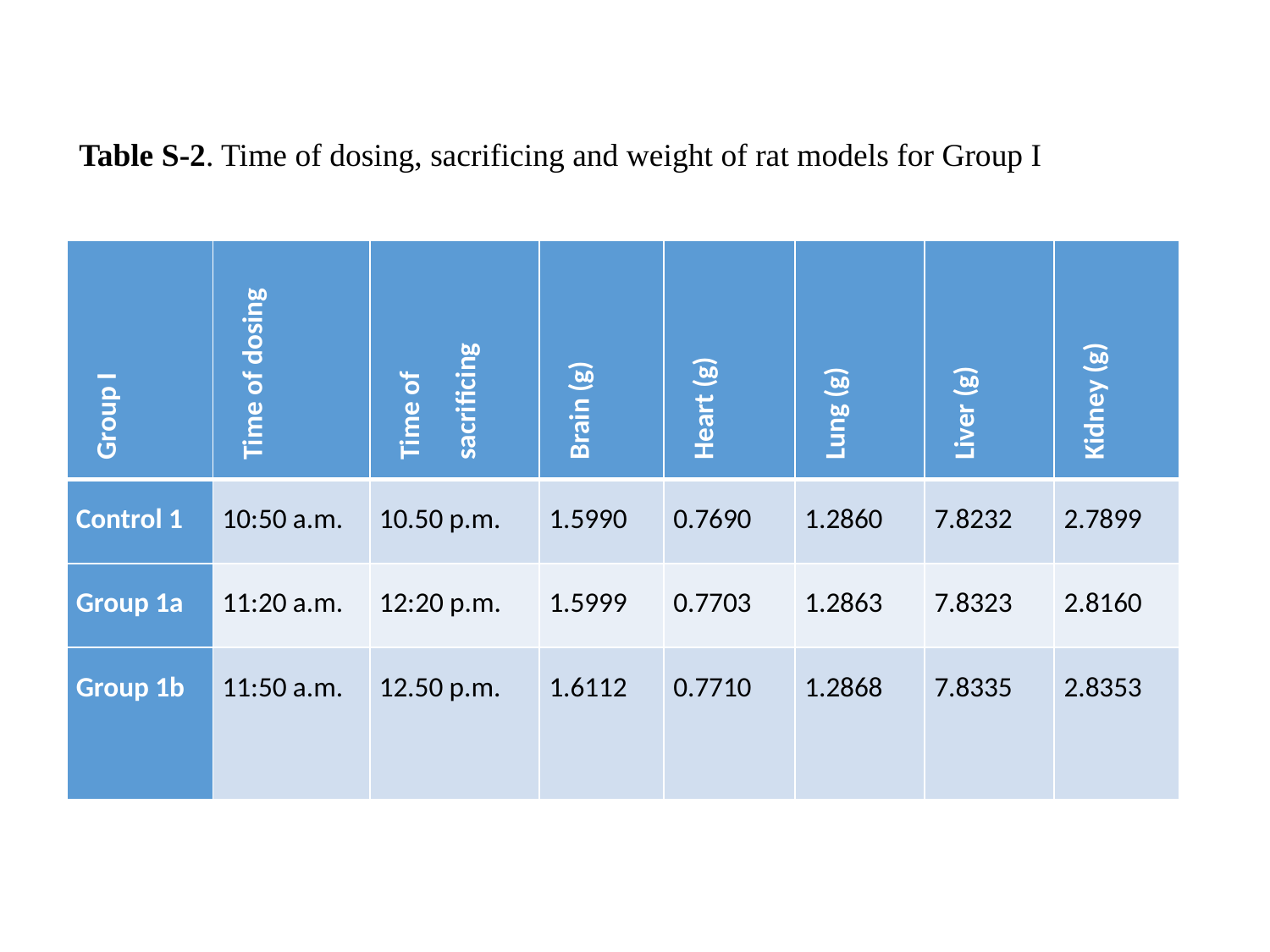

Table S-2. Time of dosing, sacrificing and weight of rat models for Group I
| Group I | Time of dosing | Time of sacrificing | Brain (g) | Heart (g) | Lung (g) | Liver (g) | Kidney (g) |
| --- | --- | --- | --- | --- | --- | --- | --- |
| Control 1 | 10:50 a.m. | 10.50 p.m. | 1.5990 | 0.7690 | 1.2860 | 7.8232 | 2.7899 |
| Group 1a | 11:20 a.m. | 12:20 p.m. | 1.5999 | 0.7703 | 1.2863 | 7.8323 | 2.8160 |
| Group 1b | 11:50 a.m. | 12.50 p.m. | 1.6112 | 0.7710 | 1.2868 | 7.8335 | 2.8353 |

## Slide 7
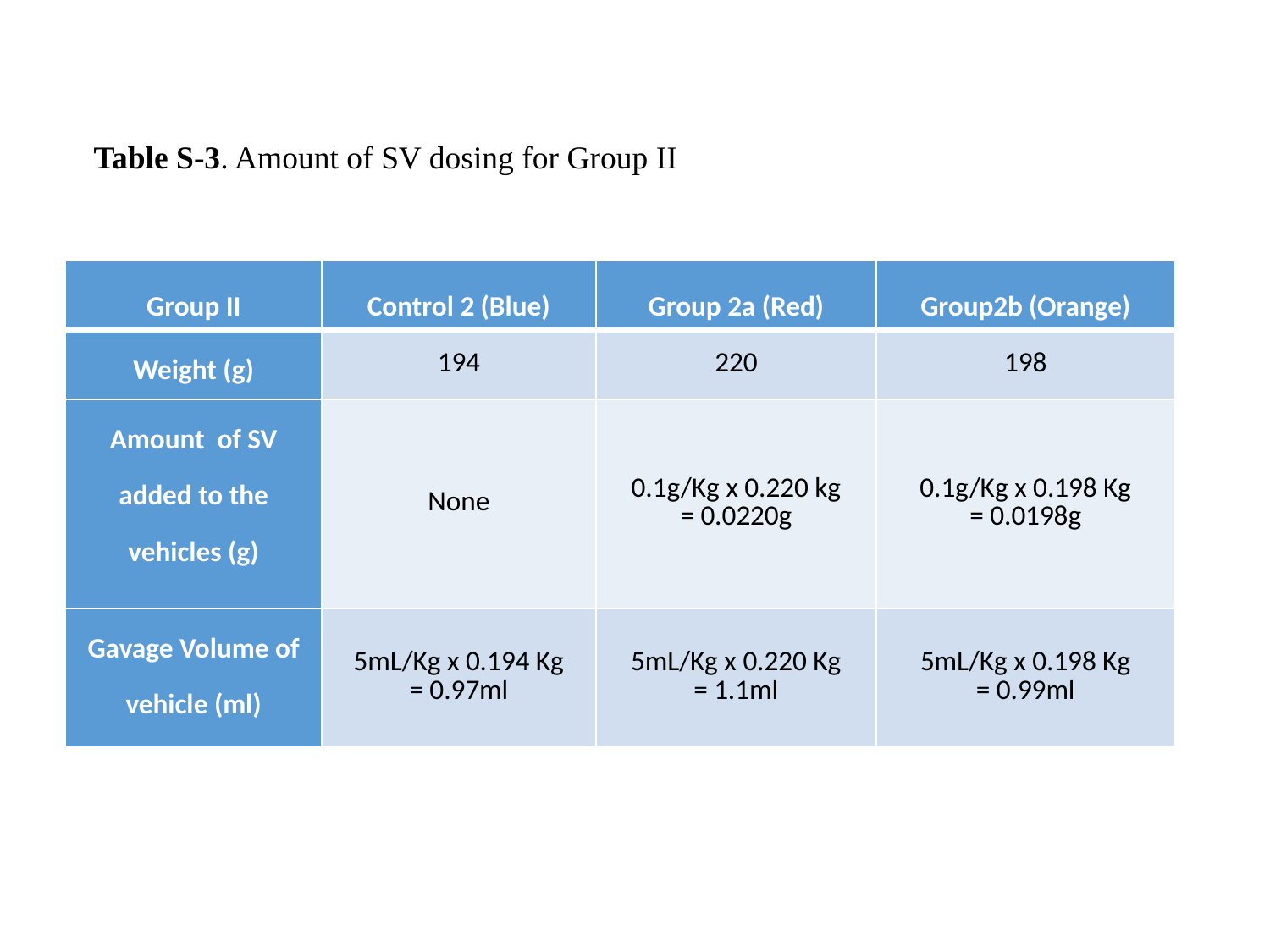

Table S-3. Amount of SV dosing for Group II
| Group II | Control 2 (Blue) | Group 2a (Red) | Group2b (Orange) |
| --- | --- | --- | --- |
| Weight (g) | 194 | 220 | 198 |
| Amount of SV added to the vehicles (g) | None | 0.1g/Kg x 0.220 kg = 0.0220g | 0.1g/Kg x 0.198 Kg = 0.0198g |
| Gavage Volume of vehicle (ml) | 5mL/Kg x 0.194 Kg = 0.97ml | 5mL/Kg x 0.220 Kg = 1.1ml | 5mL/Kg x 0.198 Kg = 0.99ml |

## Slide 8
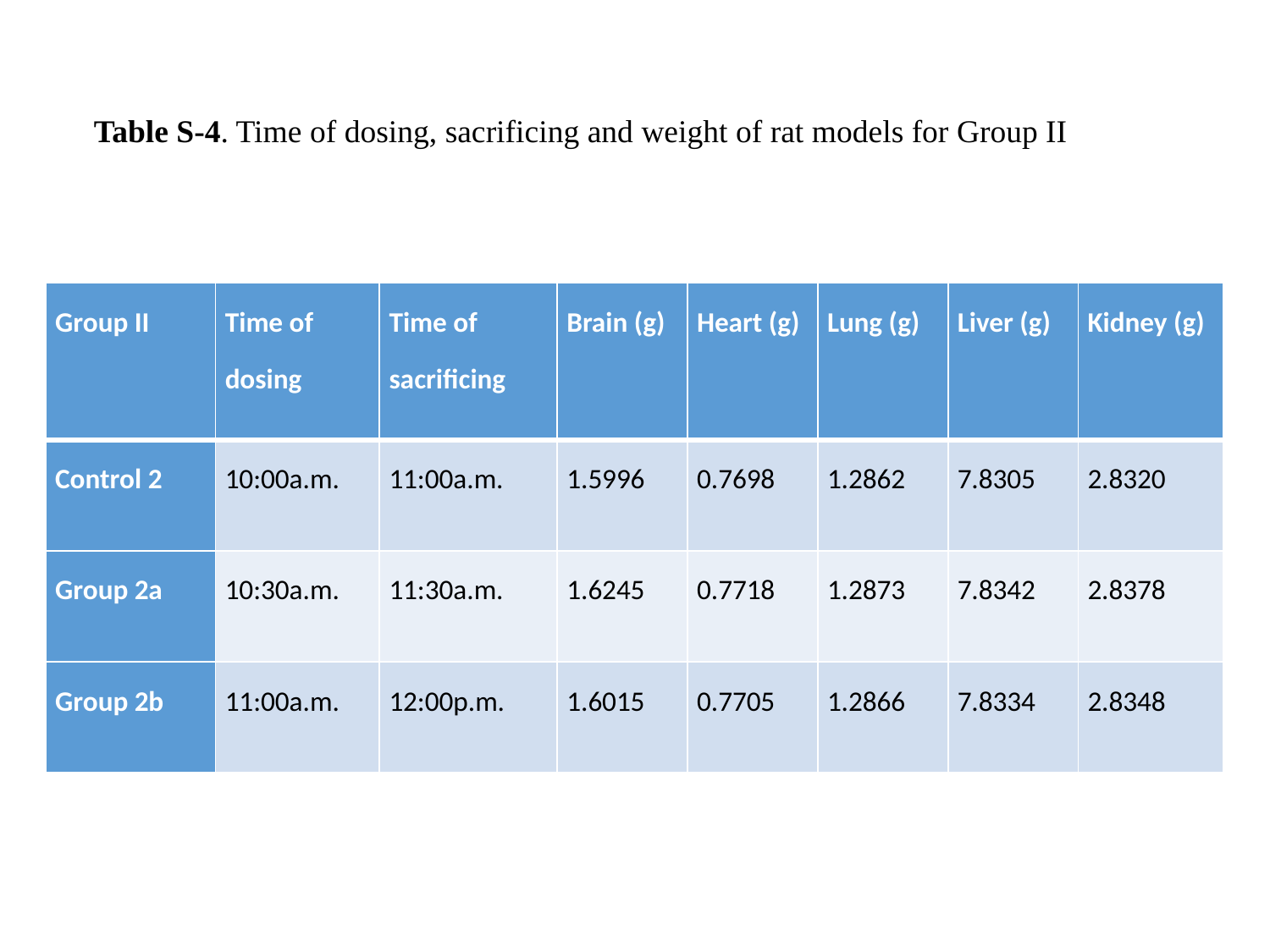

Table S-4. Time of dosing, sacrificing and weight of rat models for Group II
| Group II | Time of dosing | Time of sacrificing | Brain (g) | Heart (g) | Lung (g) | Liver (g) | Kidney (g) |
| --- | --- | --- | --- | --- | --- | --- | --- |
| Control 2 | 10:00a.m. | 11:00a.m. | 1.5996 | 0.7698 | 1.2862 | 7.8305 | 2.8320 |
| Group 2a | 10:30a.m. | 11:30a.m. | 1.6245 | 0.7718 | 1.2873 | 7.8342 | 2.8378 |
| Group 2b | 11:00a.m. | 12:00p.m. | 1.6015 | 0.7705 | 1.2866 | 7.8334 | 2.8348 |

## Slide 9
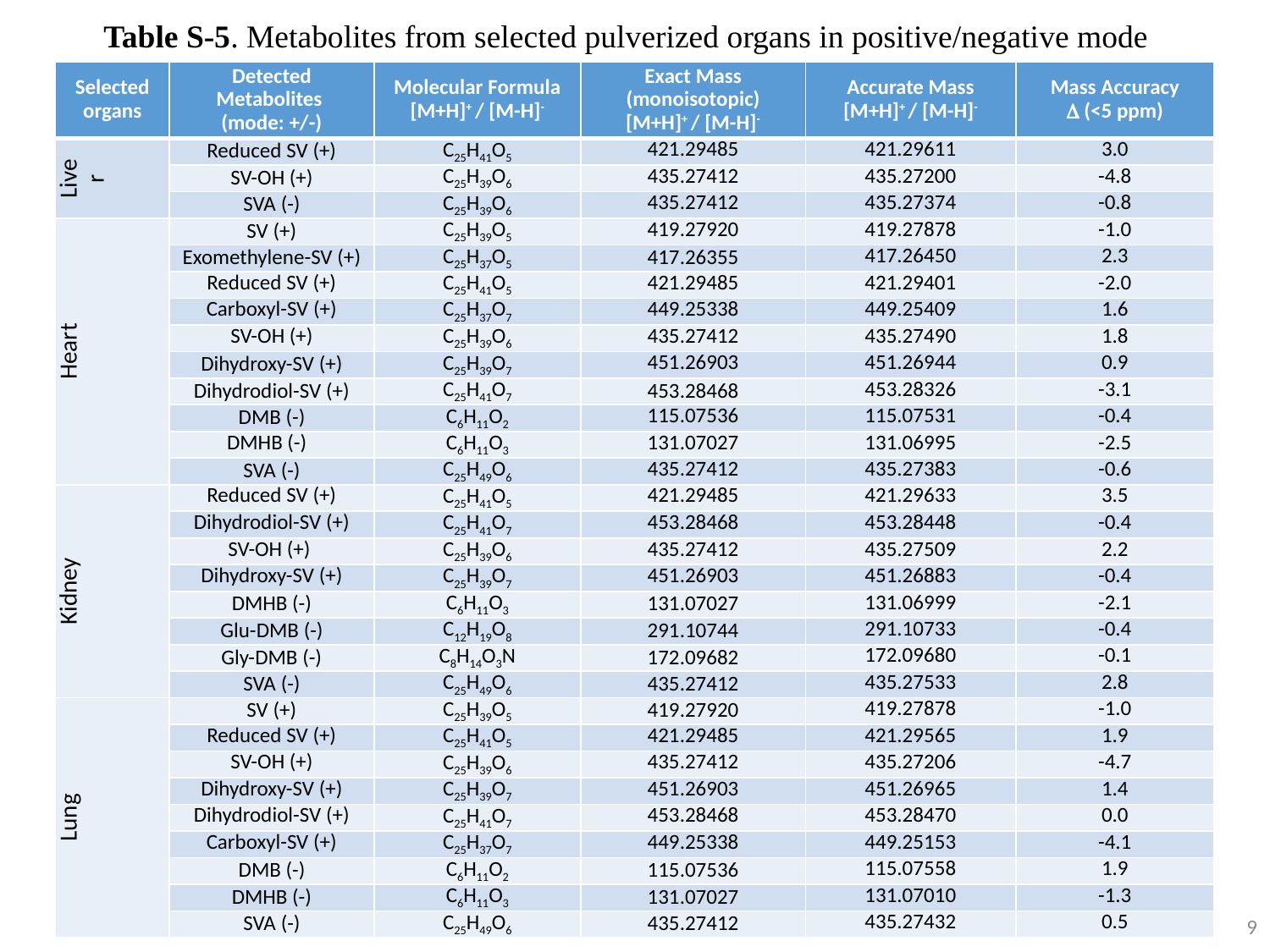

Table S-5. Metabolites from selected pulverized organs in positive/negative mode
| Selected organs | Detected Metabolites (mode: +/-) | Molecular Formula [M+H]+ / [M-H]- | Exact Mass (monoisotopic) [M+H]+ / [M-H]- | Accurate Mass [M+H]+ / [M-H]- | Mass Accuracy Δ (<5 ppm) |
| --- | --- | --- | --- | --- | --- |
| Liver | Reduced SV (+) | C25H41O5 | 421.29485 | 421.29611 | 3.0 |
| | SV-OH (+) | C25H39O6 | 435.27412 | 435.27200 | -4.8 |
| | SVA (-) | C25H39O6 | 435.27412 | 435.27374 | -0.8 |
| Heart | SV (+) | C25H39O5 | 419.27920 | 419.27878 | -1.0 |
| | Exomethylene-SV (+) | C25H37O5 | 417.26355 | 417.26450 | 2.3 |
| | Reduced SV (+) | C25H41O5 | 421.29485 | 421.29401 | -2.0 |
| | Carboxyl-SV (+) | C25H37O7 | 449.25338 | 449.25409 | 1.6 |
| | SV-OH (+) | C25H39O6 | 435.27412 | 435.27490 | 1.8 |
| | Dihydroxy-SV (+) | C25H39O7 | 451.26903 | 451.26944 | 0.9 |
| | Dihydrodiol-SV (+) | C25H41O7 | 453.28468 | 453.28326 | -3.1 |
| | DMB (-) | C6H11O2 | 115.07536 | 115.07531 | -0.4 |
| | DMHB (-) | C6H11O3 | 131.07027 | 131.06995 | -2.5 |
| | SVA (-) | C25H49O6 | 435.27412 | 435.27383 | -0.6 |
| Kidney | Reduced SV (+) | C25H41O5 | 421.29485 | 421.29633 | 3.5 |
| | Dihydrodiol-SV (+) | C25H41O7 | 453.28468 | 453.28448 | -0.4 |
| | SV-OH (+) | C25H39O6 | 435.27412 | 435.27509 | 2.2 |
| | Dihydroxy-SV (+) | C25H39O7 | 451.26903 | 451.26883 | -0.4 |
| | DMHB (-) | C6H11O3 | 131.07027 | 131.06999 | -2.1 |
| | Glu-DMB (-) | C12H19O8 | 291.10744 | 291.10733 | -0.4 |
| | Gly-DMB (-) | C8H14O3N | 172.09682 | 172.09680 | -0.1 |
| | SVA (-) | C25H49O6 | 435.27412 | 435.27533 | 2.8 |
| Lung | SV (+) | C25H39O5 | 419.27920 | 419.27878 | -1.0 |
| | Reduced SV (+) | C25H41O5 | 421.29485 | 421.29565 | 1.9 |
| | SV-OH (+) | C25H39O6 | 435.27412 | 435.27206 | -4.7 |
| | Dihydroxy-SV (+) | C25H39O7 | 451.26903 | 451.26965 | 1.4 |
| | Dihydrodiol-SV (+) | C25H41O7 | 453.28468 | 453.28470 | 0.0 |
| | Carboxyl-SV (+) | C25H37O7 | 449.25338 | 449.25153 | -4.1 |
| | DMB (-) | C6H11O2 | 115.07536 | 115.07558 | 1.9 |
| | DMHB (-) | C6H11O3 | 131.07027 | 131.07010 | -1.3 |
| | SVA (-) | C25H49O6 | 435.27412 | 435.27432 | 0.5 |
9

## Slide 10
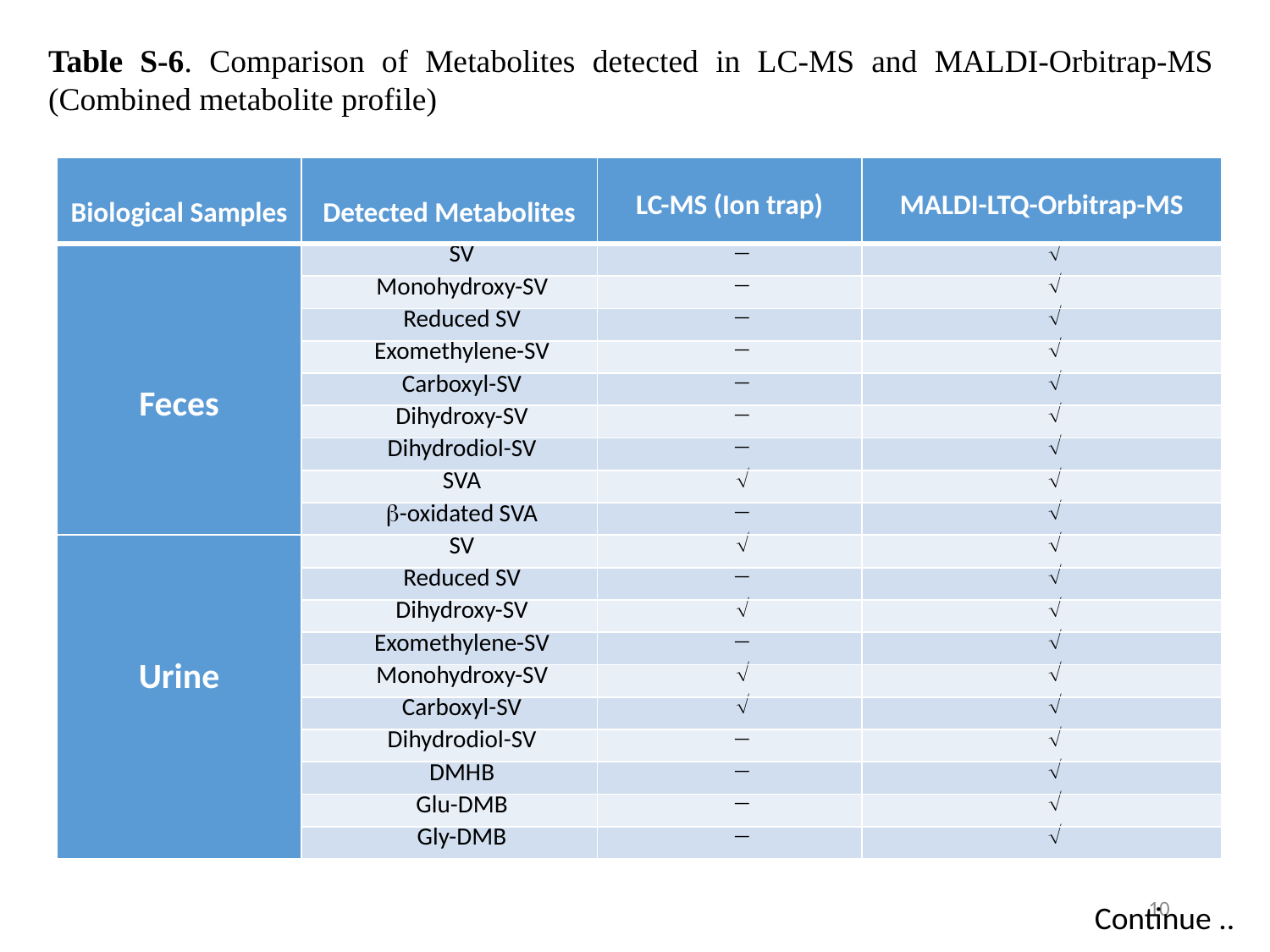

Table S-6. Comparison of Metabolites detected in LC-MS and MALDI-Orbitrap-MS (Combined metabolite profile)
| Biological Samples | Detected Metabolites | LC-MS (Ion trap) | MALDI-LTQ-Orbitrap-MS |
| --- | --- | --- | --- |
| Feces | SV |  |  |
| | Monohydroxy-SV |  |  |
| | Reduced SV |  |  |
| | Exomethylene-SV |  |  |
| | Carboxyl-SV |  |  |
| | Dihydroxy-SV |  |  |
| | Dihydrodiol-SV |  |  |
| | SVA |  |  |
| | β-oxidated SVA |  |  |
| Urine | SV |  |  |
| | Reduced SV |  |  |
| | Dihydroxy-SV |  |  |
| | Exomethylene-SV |  |  |
| | Monohydroxy-SV |  |  |
| | Carboxyl-SV |  |  |
| | Dihydrodiol-SV |  |  |
| | DMHB |  |  |
| | Glu-DMB |  |  |
| | Gly-DMB |  |  |
10
Continue ..

## Slide 11
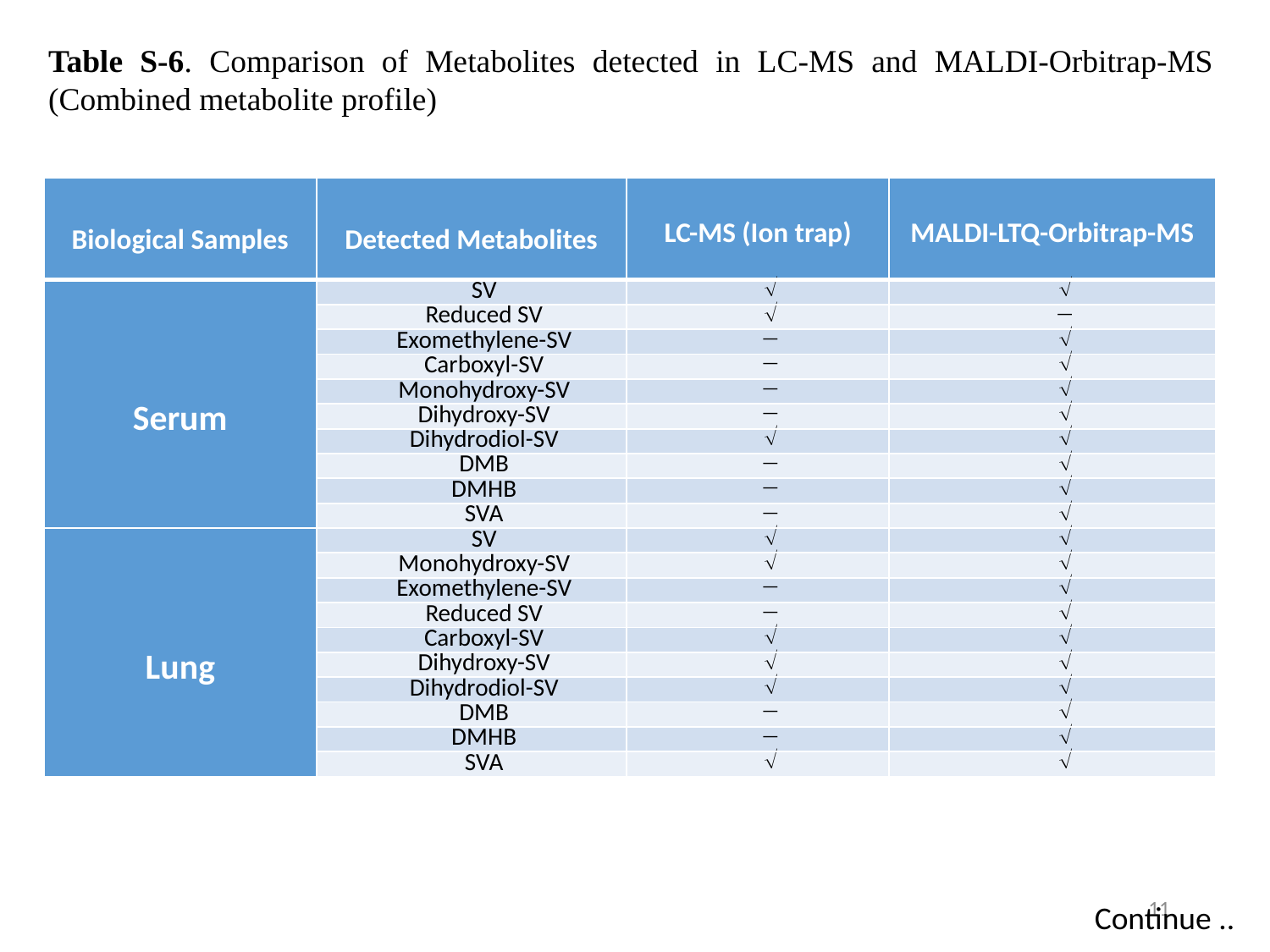

Table S-6. Comparison of Metabolites detected in LC-MS and MALDI-Orbitrap-MS (Combined metabolite profile)
| Biological Samples | Detected Metabolites | LC-MS (Ion trap) | MALDI-LTQ-Orbitrap-MS |
| --- | --- | --- | --- |
| Serum | SV |  |  |
| | Reduced SV |  |  |
| | Exomethylene-SV |  |  |
| | Carboxyl-SV |  |  |
| | Monohydroxy-SV |  |  |
| | Dihydroxy-SV |  |  |
| | Dihydrodiol-SV |  |  |
| | DMB |  |  |
| | DMHB |  |  |
| | SVA |  |  |
| Lung | SV |  |  |
| | Monohydroxy-SV |  |  |
| | Exomethylene-SV |  |  |
| | Reduced SV |  |  |
| | Carboxyl-SV |  |  |
| | Dihydroxy-SV |  |  |
| | Dihydrodiol-SV |  |  |
| | DMB |  |  |
| | DMHB |  |  |
| | SVA |  |  |
11
Continue ..

## Slide 12
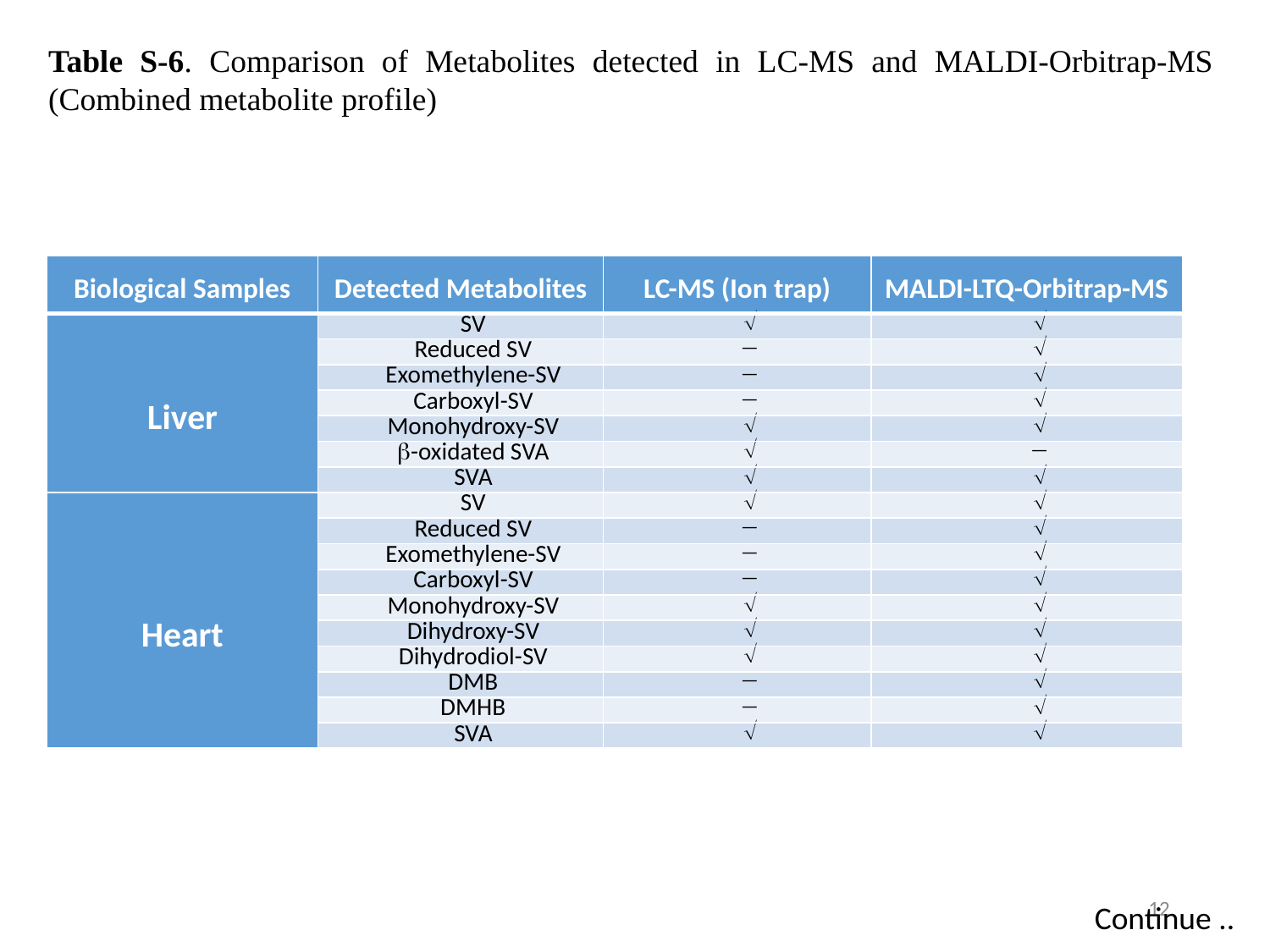

Table S-6. Comparison of Metabolites detected in LC-MS and MALDI-Orbitrap-MS (Combined metabolite profile)
| Biological Samples | Detected Metabolites | LC-MS (Ion trap) | MALDI-LTQ-Orbitrap-MS |
| --- | --- | --- | --- |
| Liver | SV |  |  |
| | Reduced SV |  |  |
| | Exomethylene-SV |  |  |
| | Carboxyl-SV |  |  |
| | Monohydroxy-SV |  |  |
| | β-oxidated SVA |  |  |
| | SVA |  |  |
| Heart | SV |  |  |
| | Reduced SV |  |  |
| | Exomethylene-SV |  |  |
| | Carboxyl-SV |  |  |
| | Monohydroxy-SV |  |  |
| | Dihydroxy-SV |  |  |
| | Dihydrodiol-SV |  |  |
| | DMB |  |  |
| | DMHB |  |  |
| | SVA |  |  |
12
Continue ..

## Slide 13
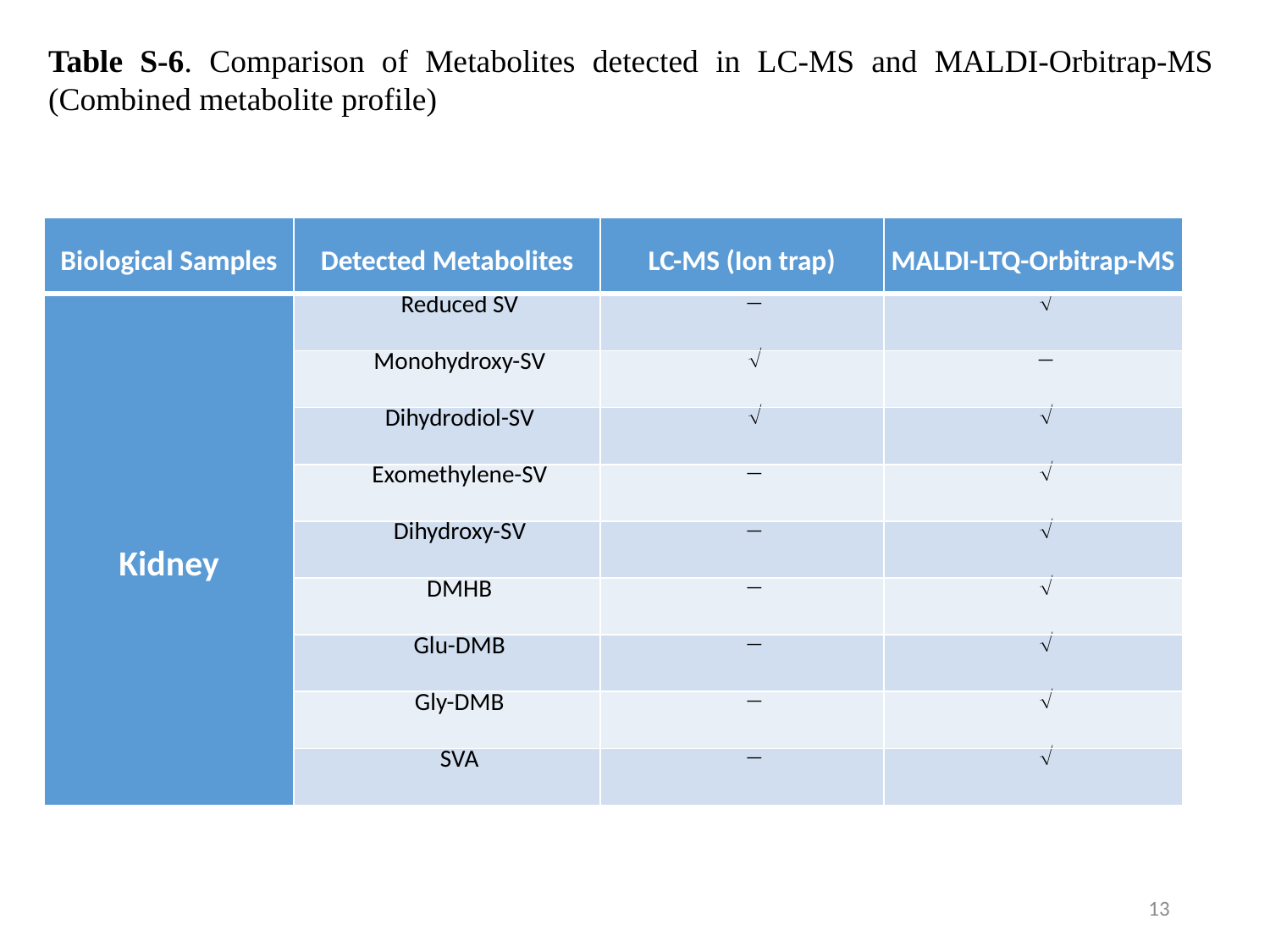

Table S-6. Comparison of Metabolites detected in LC-MS and MALDI-Orbitrap-MS (Combined metabolite profile)
| Biological Samples | Detected Metabolites | LC-MS (Ion trap) | MALDI-LTQ-Orbitrap-MS |
| --- | --- | --- | --- |
| Kidney | Reduced SV |  |  |
| | Monohydroxy-SV |  |  |
| | Dihydrodiol-SV |  |  |
| | Exomethylene-SV |  |  |
| | Dihydroxy-SV |  |  |
| | DMHB |  |  |
| | Glu-DMB |  |  |
| | Gly-DMB |  |  |
| | SVA |  |  |
13

## Slide 14
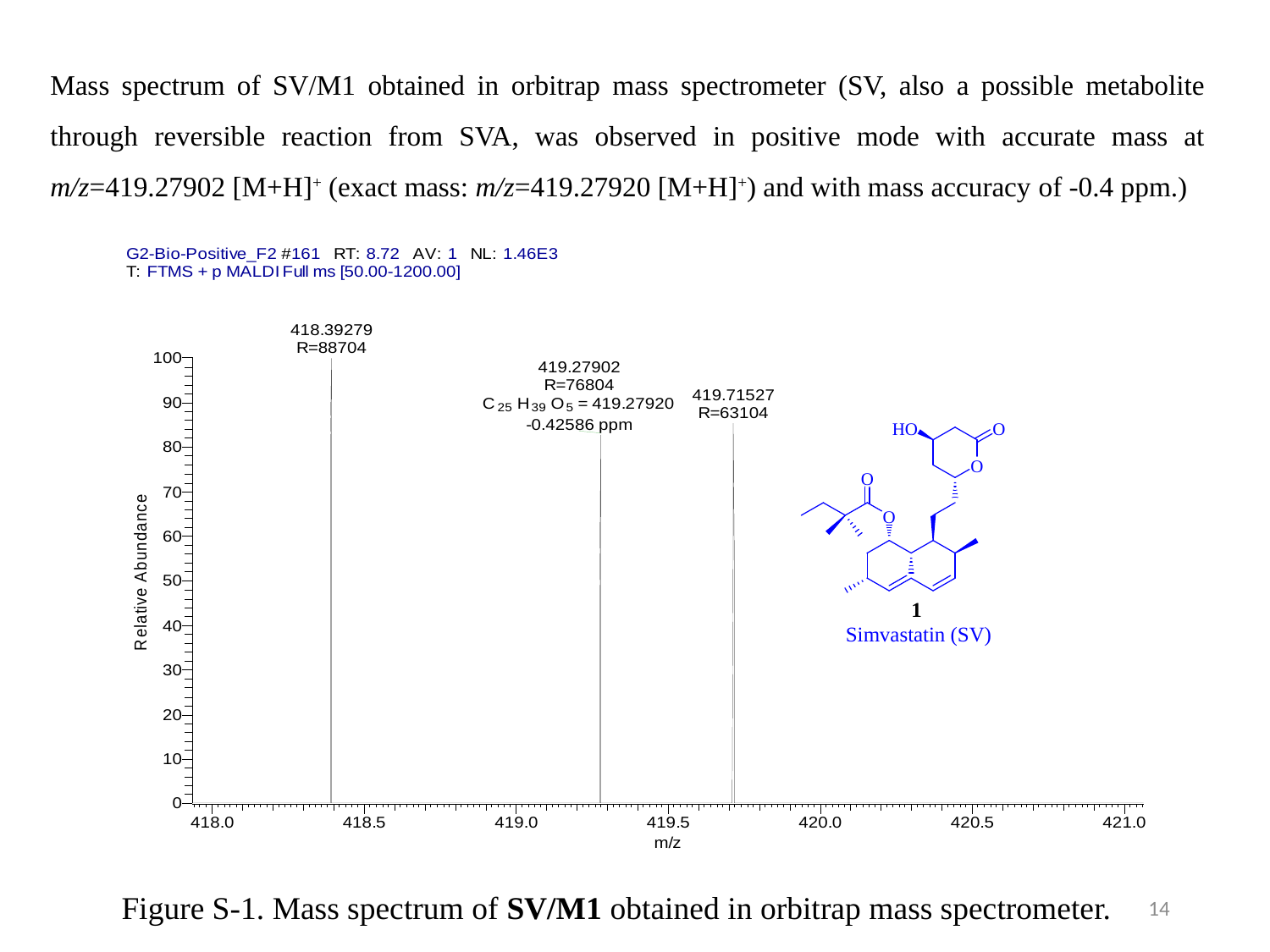

Mass spectrum of SV/M1 obtained in orbitrap mass spectrometer (SV, also a possible metabolite through reversible reaction from SVA, was observed in positive mode with accurate mass at m/z=419.27902 [M+H]+ (exact mass: m/z=419.27920 [M+H]+) and with mass accuracy of -0.4 ppm.)
Figure S-1. Mass spectrum of SV/M1 obtained in orbitrap mass spectrometer.
14

## Slide 15
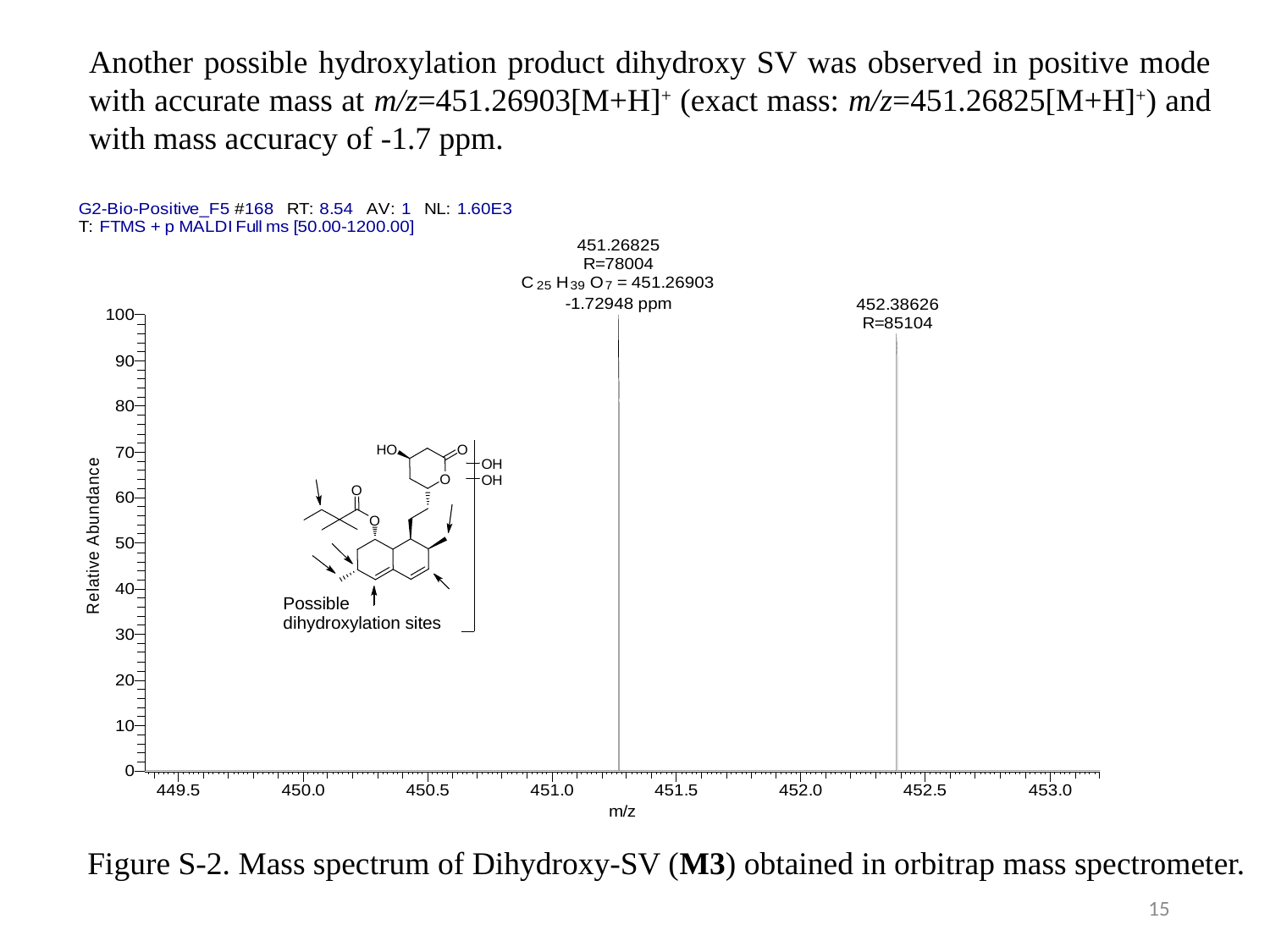

Another possible hydroxylation product dihydroxy SV was observed in positive mode with accurate mass at m/z=451.26903[M+H]+ (exact mass: m/z=451.26825[M+H]+) and with mass accuracy of -1.7 ppm.
Figure S-2. Mass spectrum of Dihydroxy-SV (M3) obtained in orbitrap mass spectrometer.
15

## Slide 16
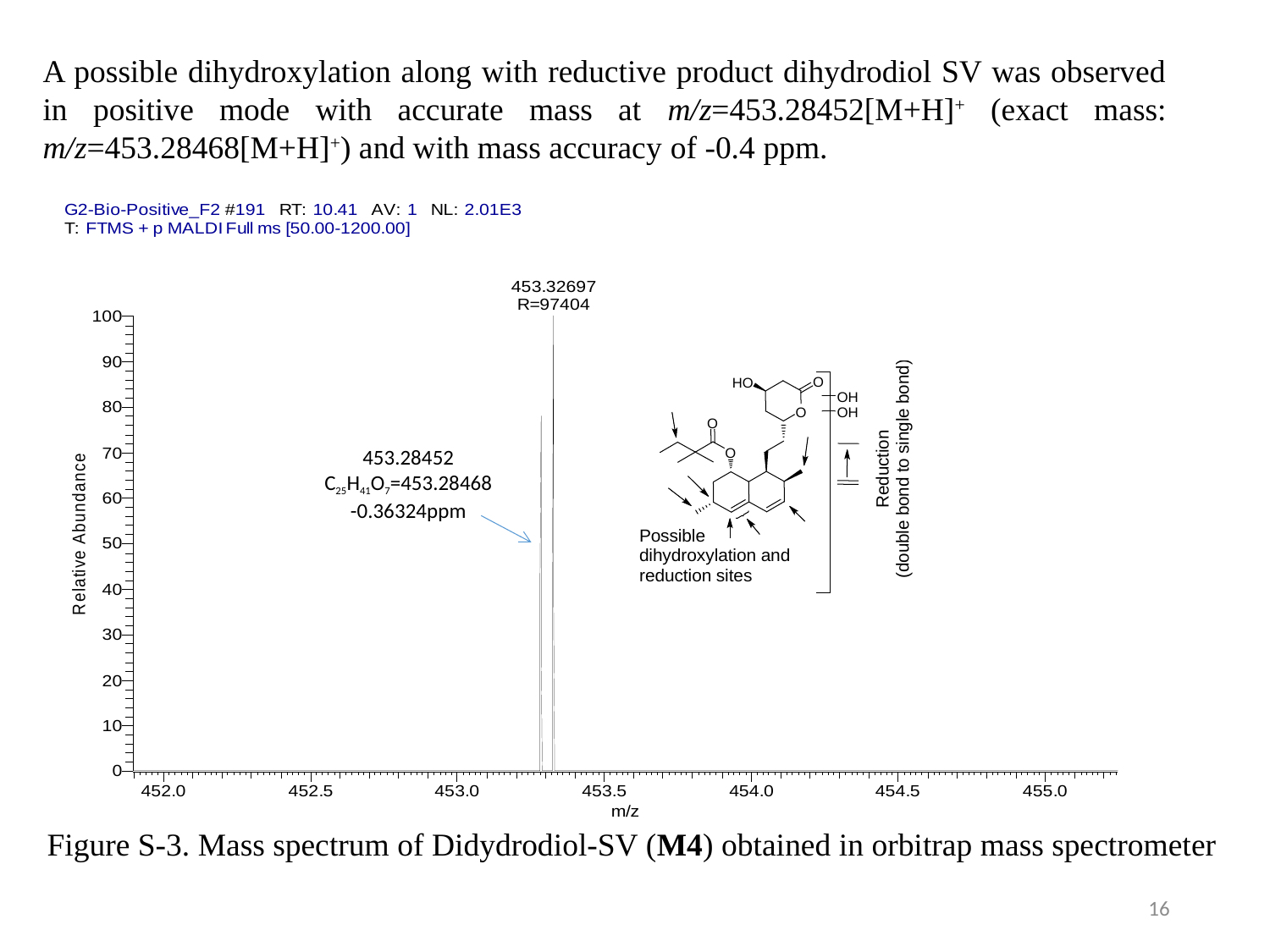

A possible dihydroxylation along with reductive product dihydrodiol SV was observed in positive mode with accurate mass at m/z=453.28452[M+H]+ (exact mass: m/z=453.28468[M+H]+) and with mass accuracy of -0.4 ppm.
453.28452
C25H41O7=453.28468
-0.36324ppm
Figure S-3. Mass spectrum of Didydrodiol-SV (M4) obtained in orbitrap mass spectrometer
16

## Slide 17
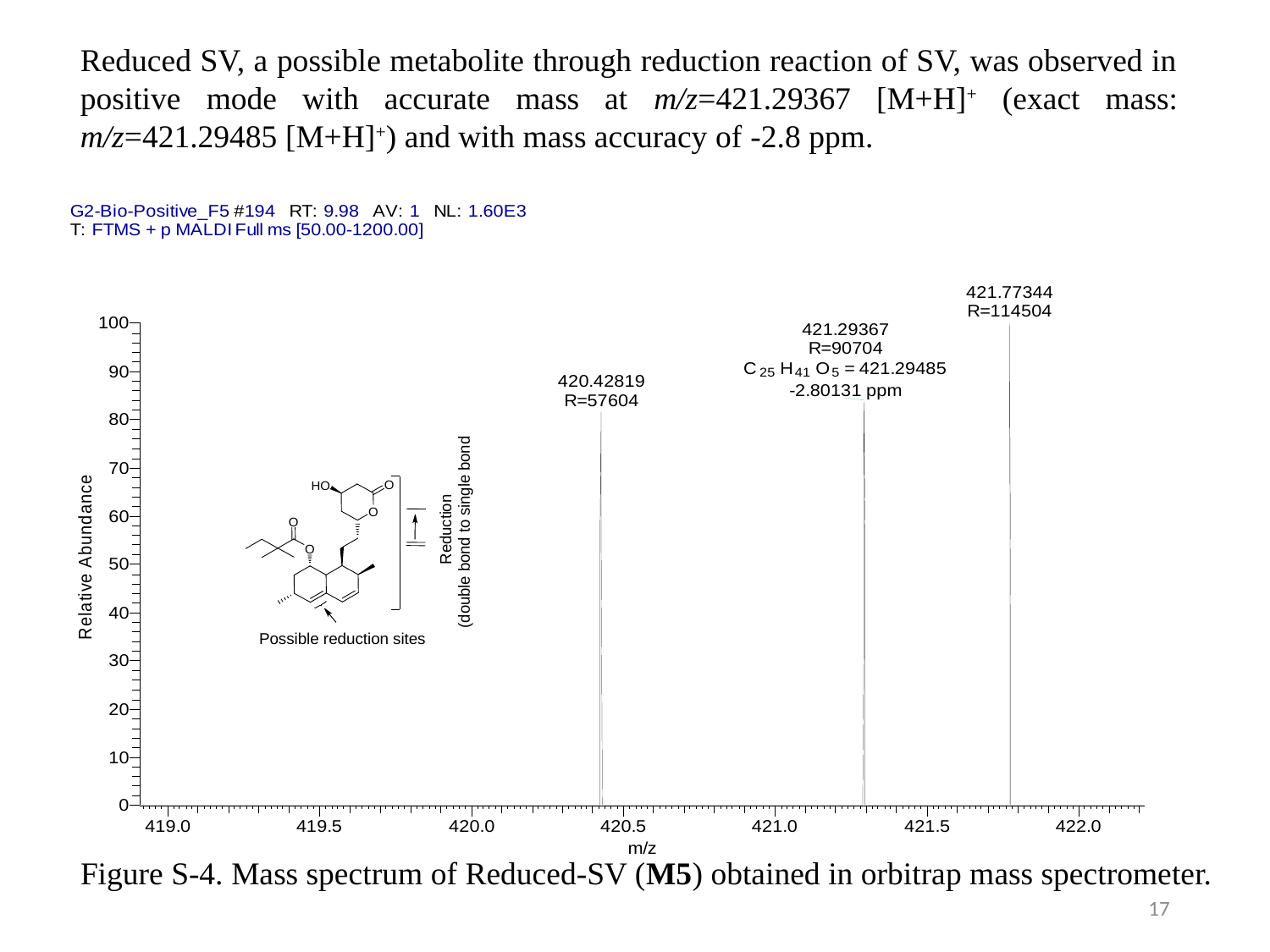

Reduced SV, a possible metabolite through reduction reaction of SV, was observed in positive mode with accurate mass at m/z=421.29367 [M+H]+ (exact mass: m/z=421.29485 [M+H]+) and with mass accuracy of -2.8 ppm.
Figure S-4. Mass spectrum of Reduced-SV (M5) obtained in orbitrap mass spectrometer.
17

## Slide 18
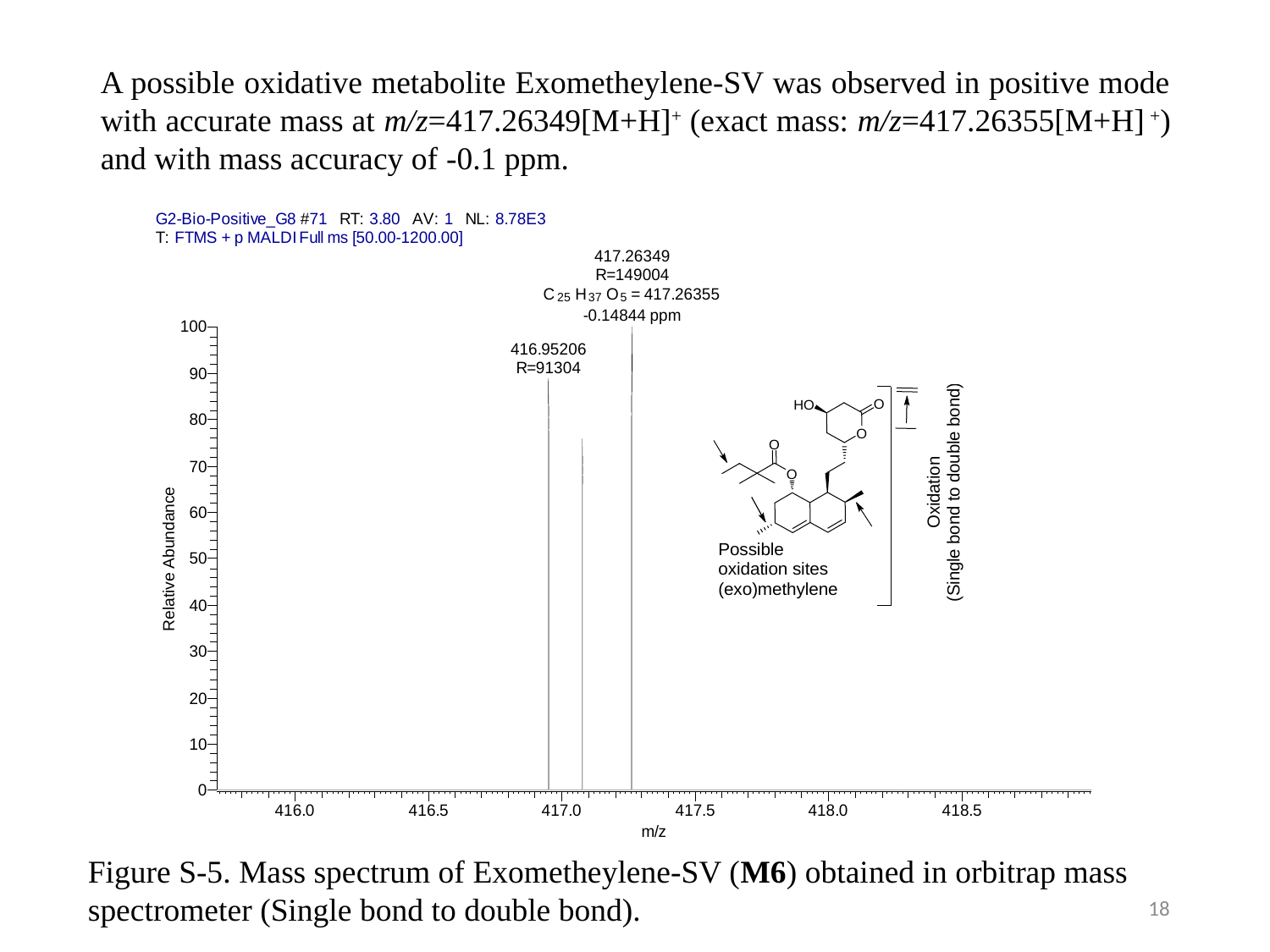

A possible oxidative metabolite Exometheylene-SV was observed in positive mode with accurate mass at m/z=417.26349[M+H]+ (exact mass: m/z=417.26355[M+H] +) and with mass accuracy of -0.1 ppm.
Figure S-5. Mass spectrum of Exometheylene-SV (M6) obtained in orbitrap mass spectrometer (Single bond to double bond).
18

## Slide 19
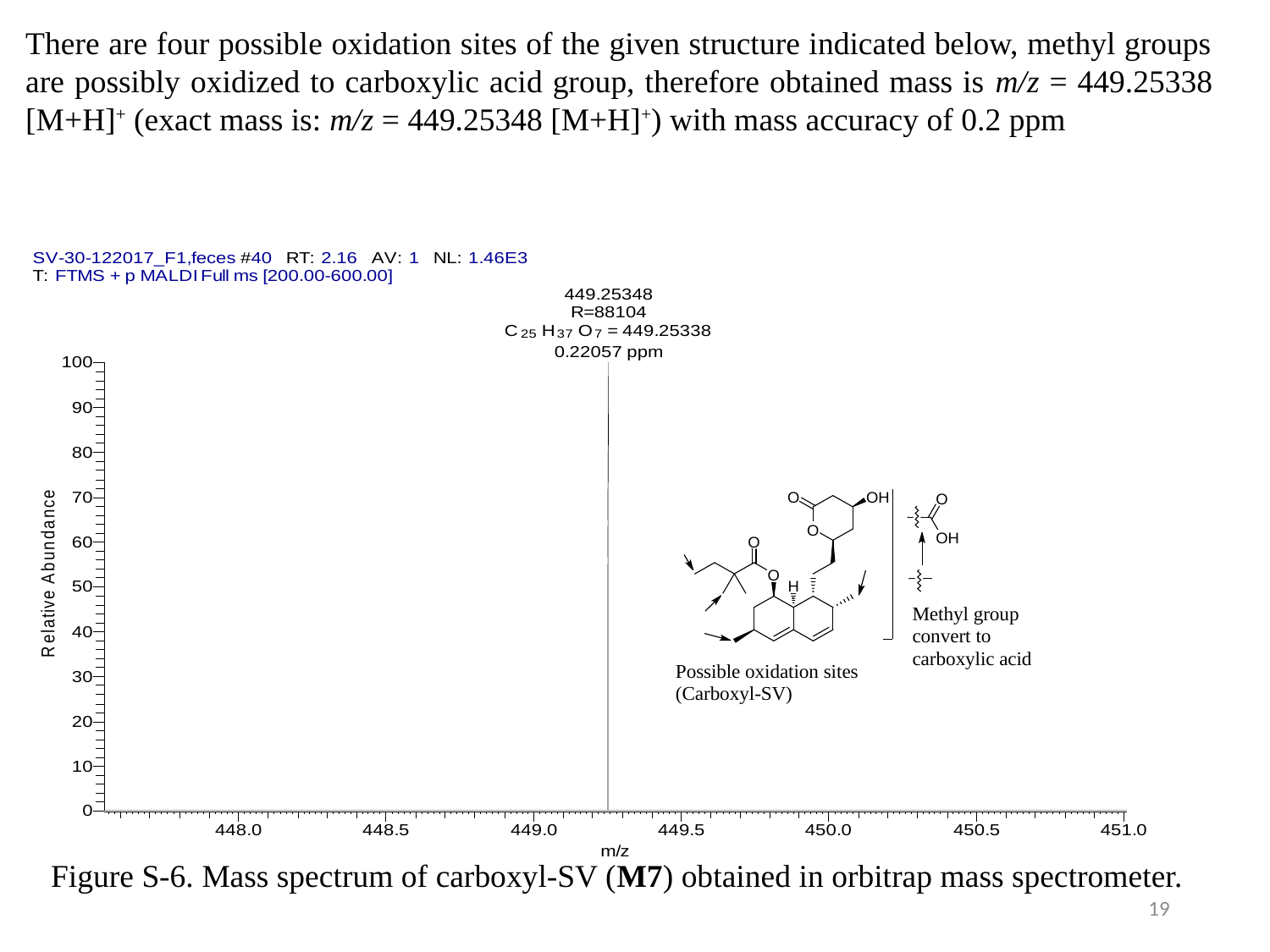

There are four possible oxidation sites of the given structure indicated below, methyl groups are possibly oxidized to carboxylic acid group, therefore obtained mass is m/z = 449.25338 [M+H]+ (exact mass is: m/z = 449.25348 [M+H]+) with mass accuracy of 0.2 ppm
Figure S-6. Mass spectrum of carboxyl-SV (M7) obtained in orbitrap mass spectrometer.
19

## Slide 20
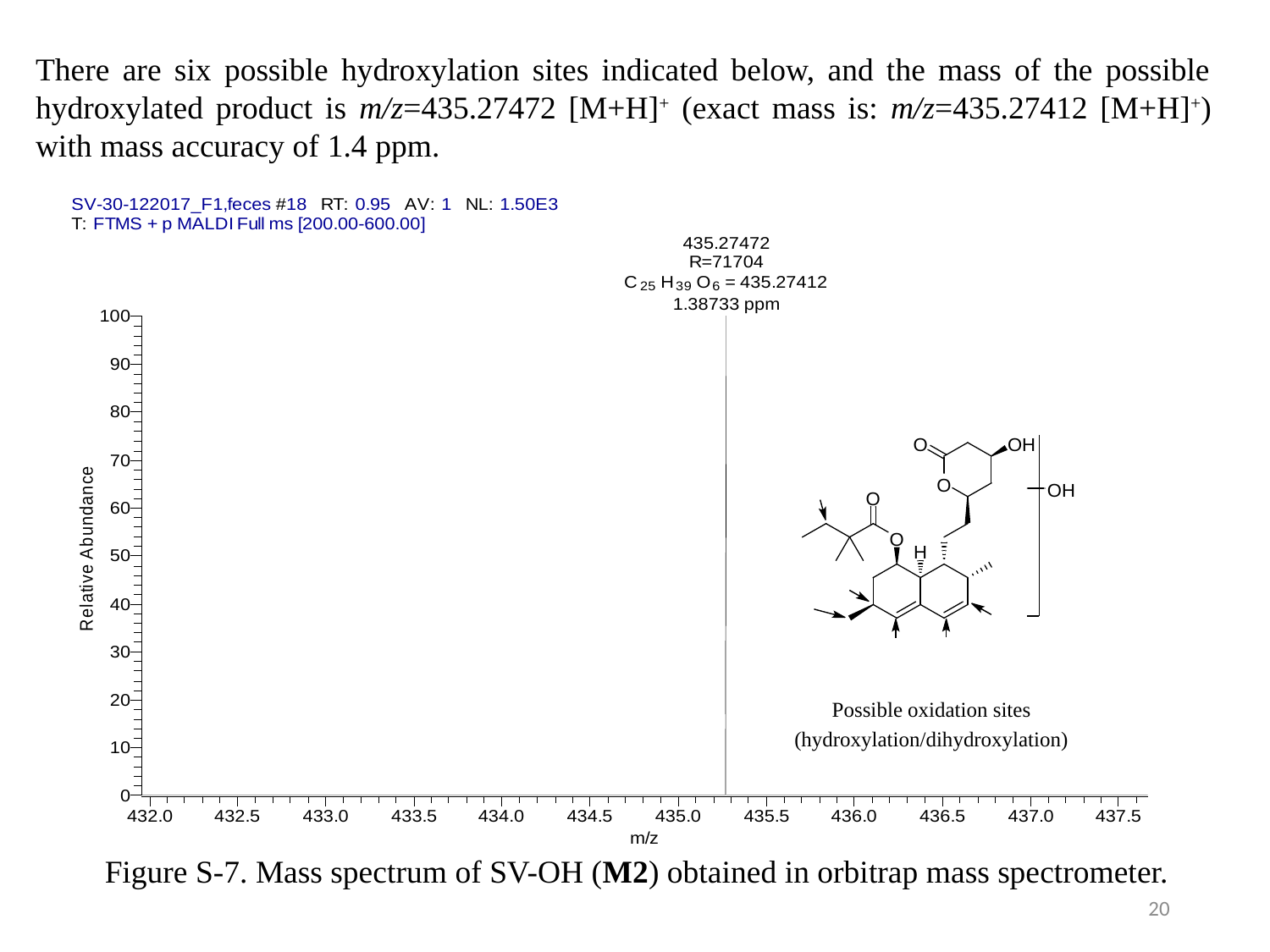

There are six possible hydroxylation sites indicated below, and the mass of the possible hydroxylated product is m/z=435.27472 [M+H]+ (exact mass is: m/z=435.27412 [M+H]+) with mass accuracy of 1.4 ppm.
Possible oxidation sites
(hydroxylation/dihydroxylation)
Figure S-7. Mass spectrum of SV-OH (M2) obtained in orbitrap mass spectrometer.
20

## Slide 21
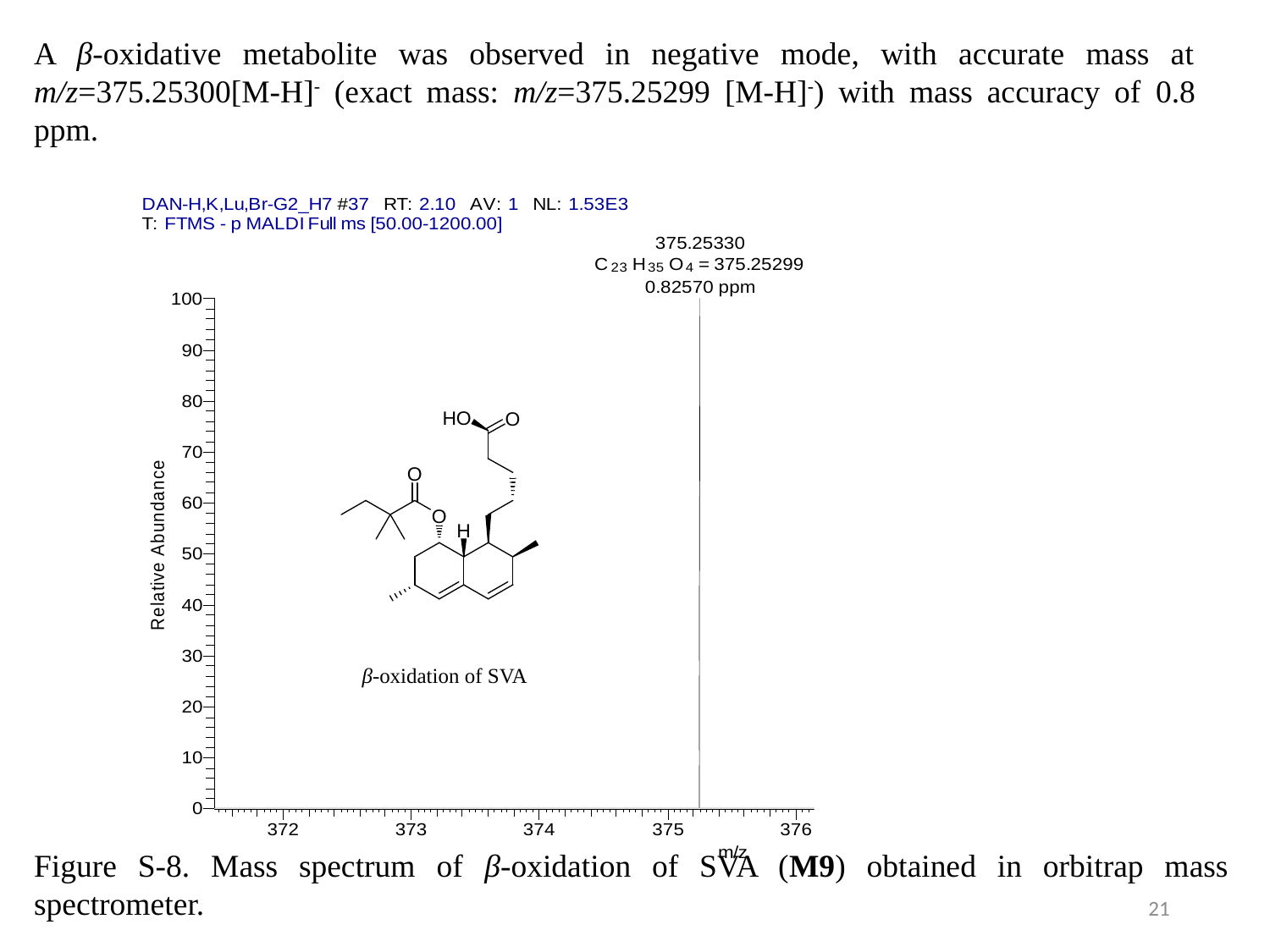

A β-oxidative metabolite was observed in negative mode, with accurate mass at m/z=375.25300[M-H]- (exact mass: m/z=375.25299 [M-H]-) with mass accuracy of 0.8 ppm.
β-oxidation of SVA
Figure S-8. Mass spectrum of β-oxidation of SVA (M9) obtained in orbitrap mass spectrometer.
21

## Slide 22
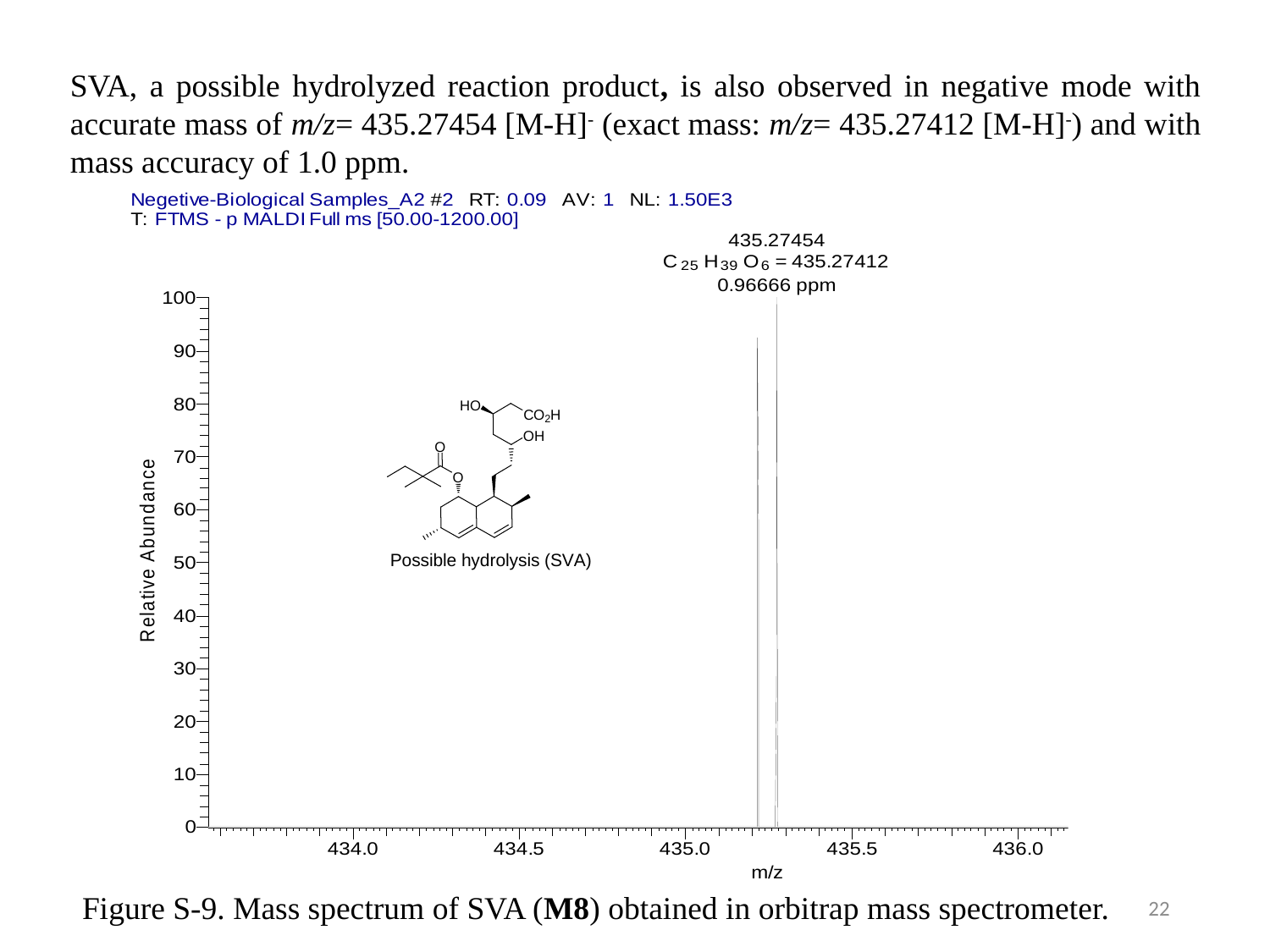

SVA, a possible hydrolyzed reaction product, is also observed in negative mode with accurate mass of m/z= 435.27454 [M-H]- (exact mass: m/z= 435.27412 [M-H]-) and with mass accuracy of 1.0 ppm.
Figure S-9. Mass spectrum of SVA (M8) obtained in orbitrap mass spectrometer.
22

## Slide 23
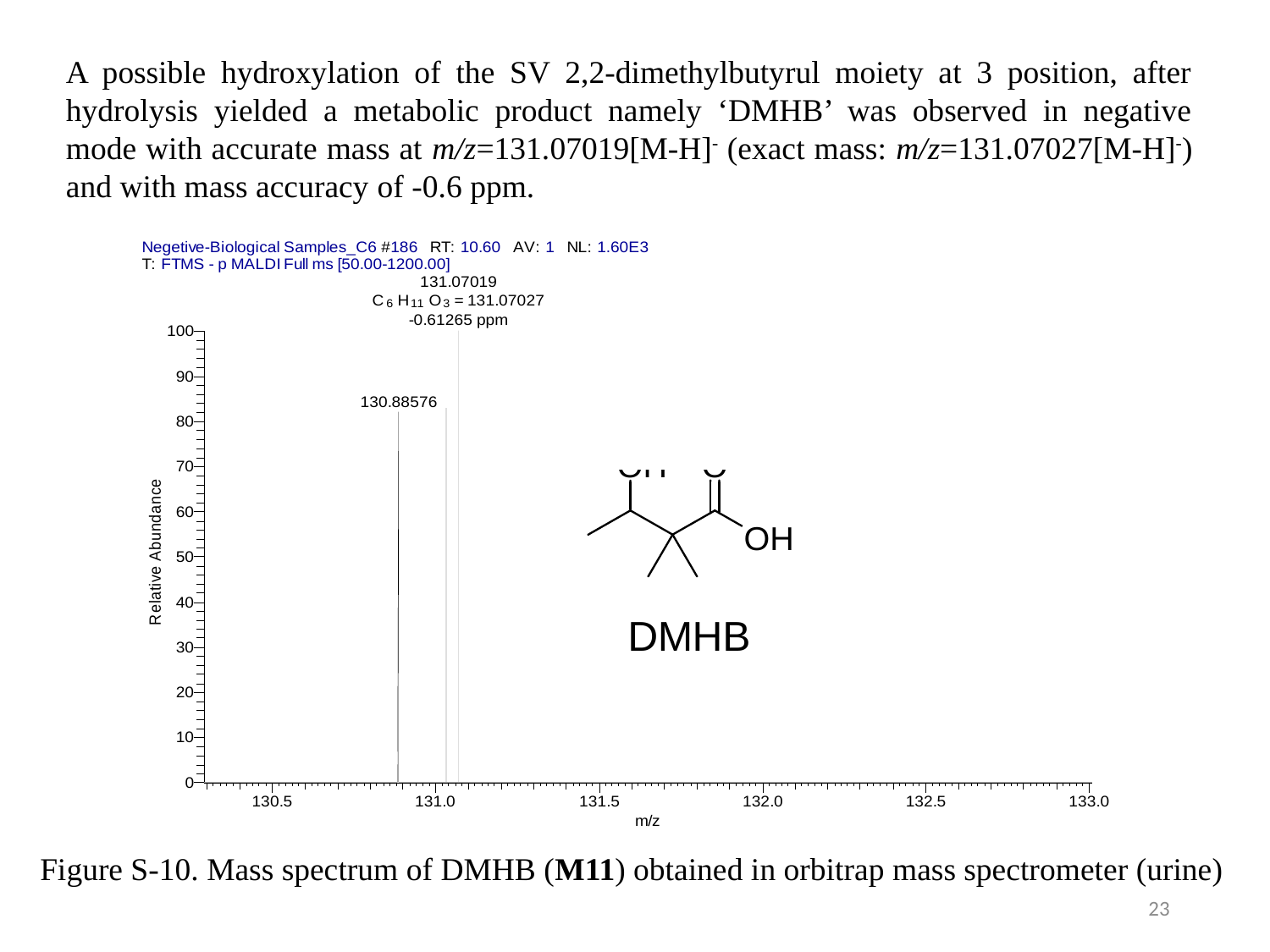

A possible hydroxylation of the SV 2,2-dimethylbutyrul moiety at 3 position, after hydrolysis yielded a metabolic product namely ‘DMHB’ was observed in negative mode with accurate mass at m/z=131.07019[M-H]- (exact mass: m/z=131.07027[M-H]-) and with mass accuracy of -0.6 ppm.
Figure S-10. Mass spectrum of DMHB (M11) obtained in orbitrap mass spectrometer (urine)
23

## Slide 24
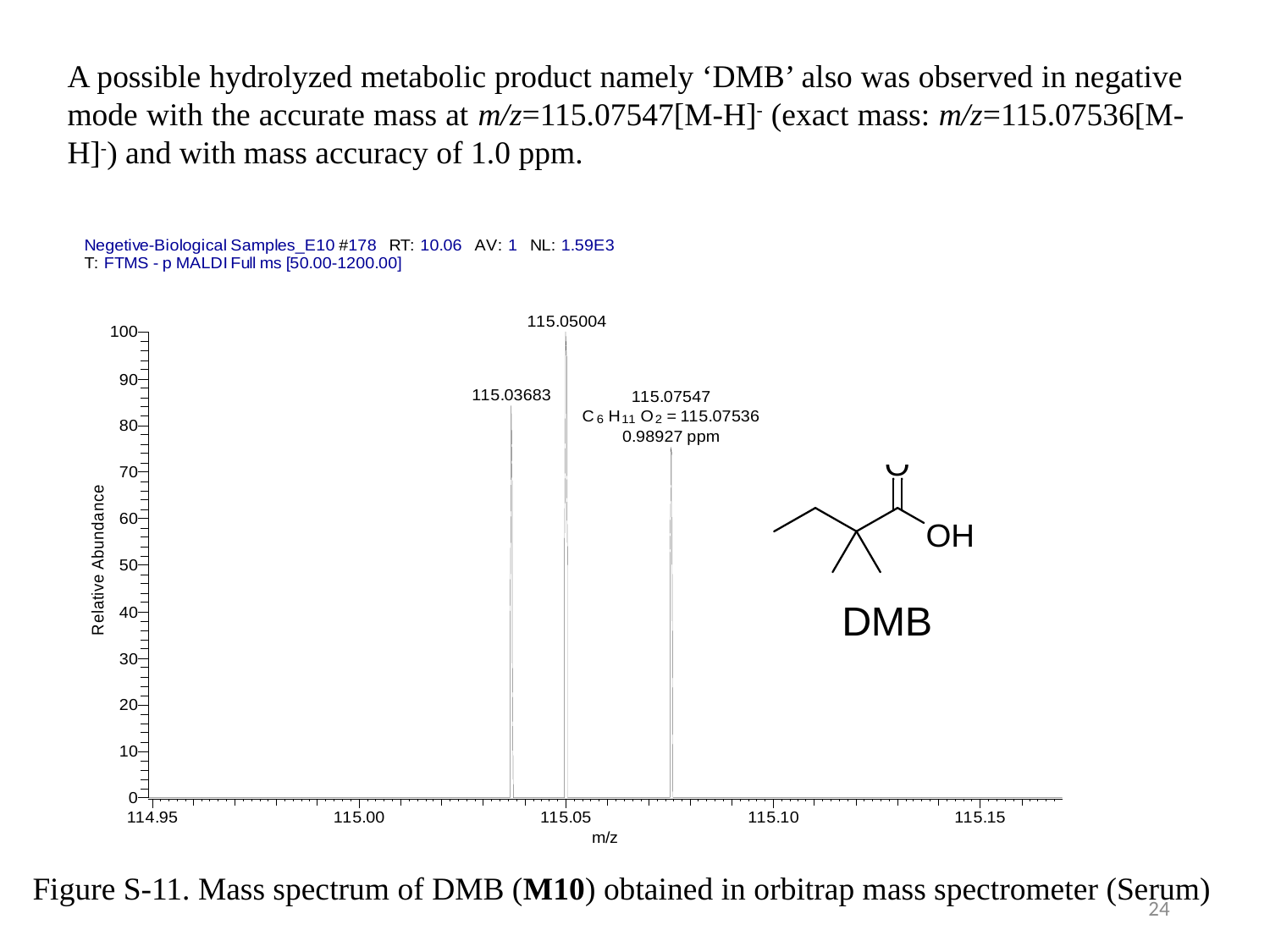

A possible hydrolyzed metabolic product namely ‘DMB’ also was observed in negative mode with the accurate mass at m/z=115.07547[M-H]- (exact mass: m/z=115.07536[M-H]-) and with mass accuracy of 1.0 ppm.
Figure S-11. Mass spectrum of DMB (M10) obtained in orbitrap mass spectrometer (Serum)
24

## Slide 25
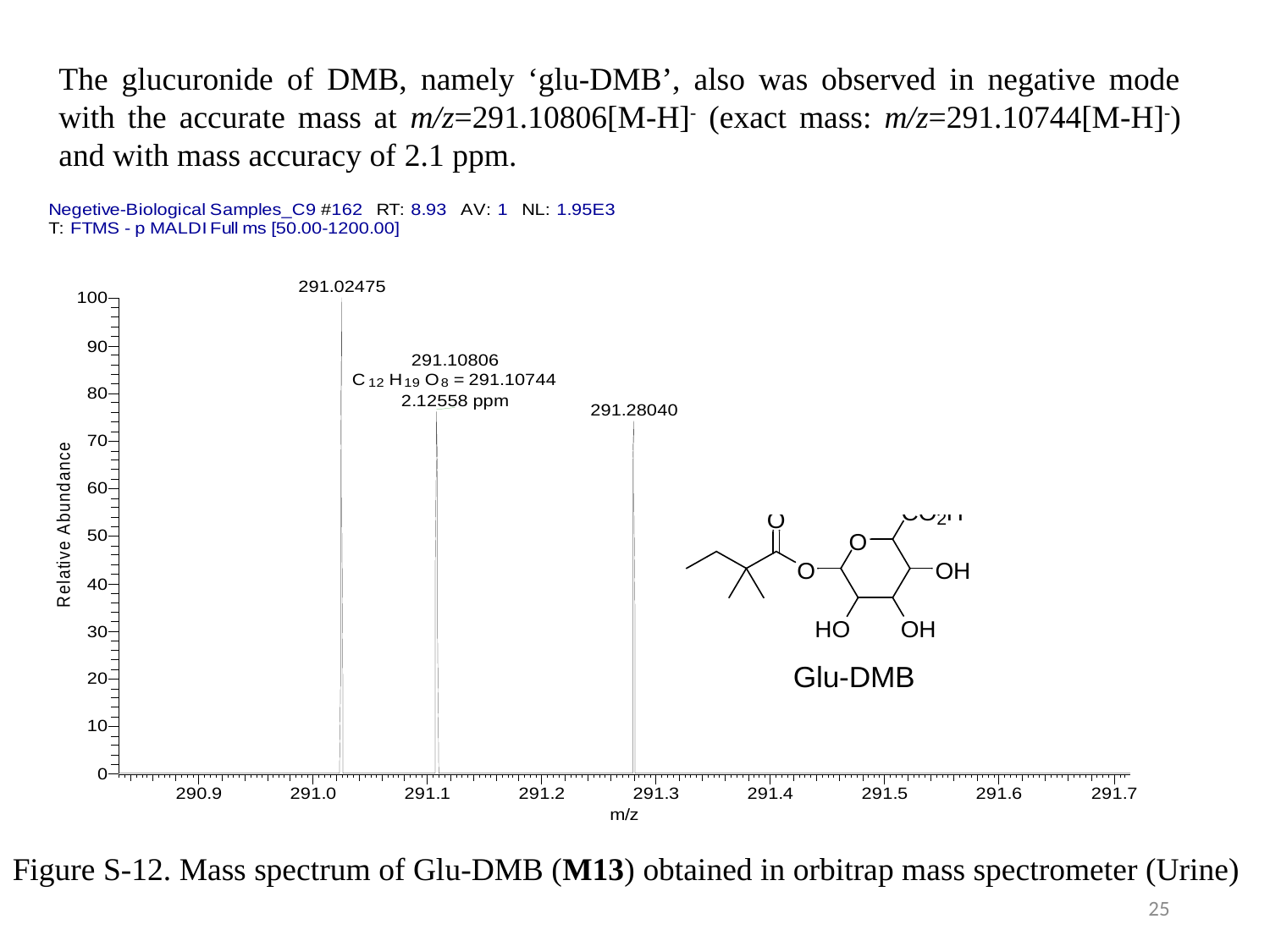

The glucuronide of DMB, namely ‘glu-DMB’, also was observed in negative mode with the accurate mass at m/z=291.10806[M-H]- (exact mass: m/z=291.10744[M-H]-) and with mass accuracy of 2.1 ppm.
Figure S-12. Mass spectrum of Glu-DMB (M13) obtained in orbitrap mass spectrometer (Urine)
25

## Slide 26
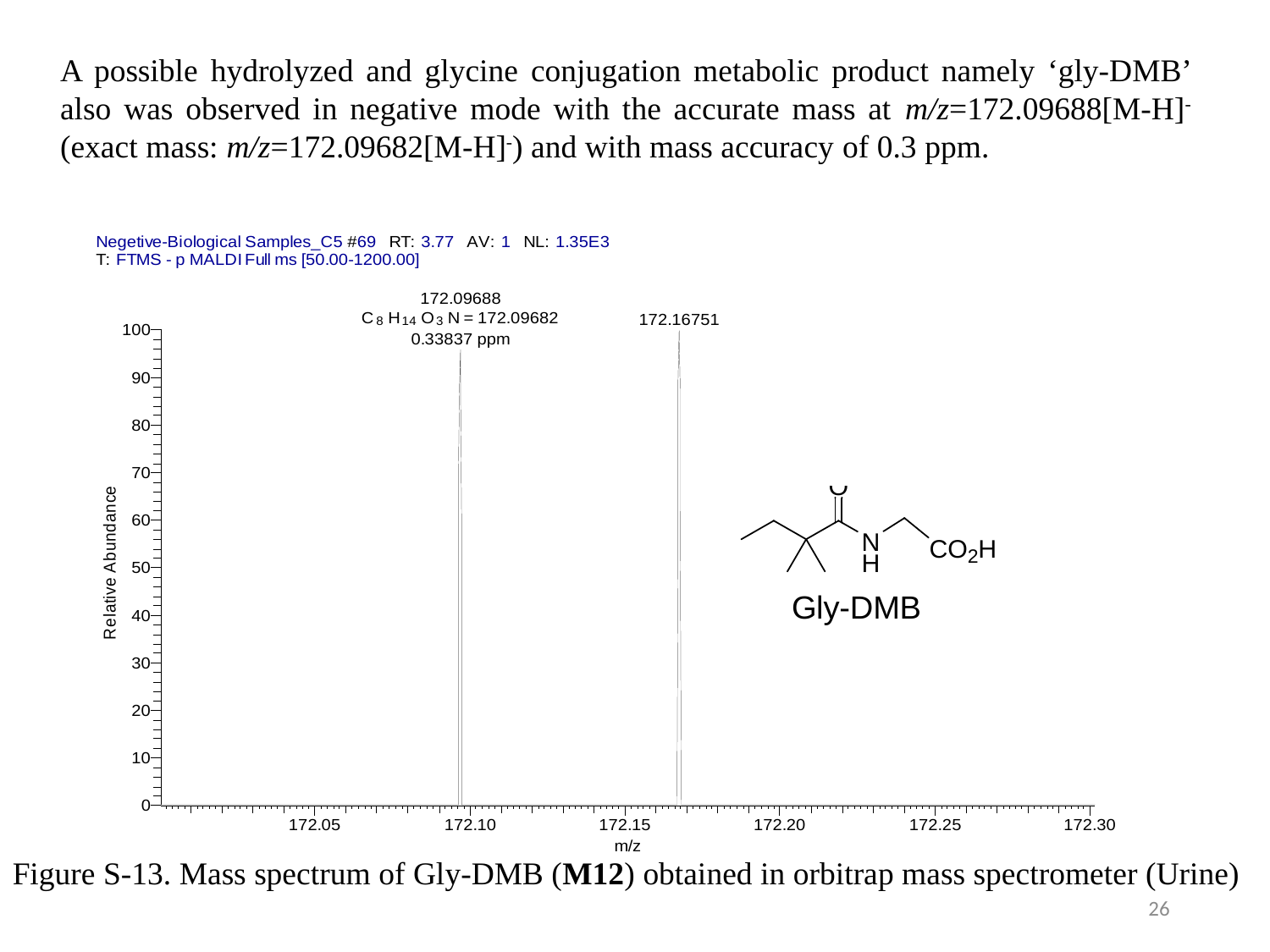

A possible hydrolyzed and glycine conjugation metabolic product namely ‘gly-DMB’ also was observed in negative mode with the accurate mass at m/z=172.09688[M-H]- (exact mass: m/z=172.09682[M-H]-) and with mass accuracy of 0.3 ppm.
Figure S-13. Mass spectrum of Gly-DMB (M12) obtained in orbitrap mass spectrometer (Urine)
26

## Slide 27
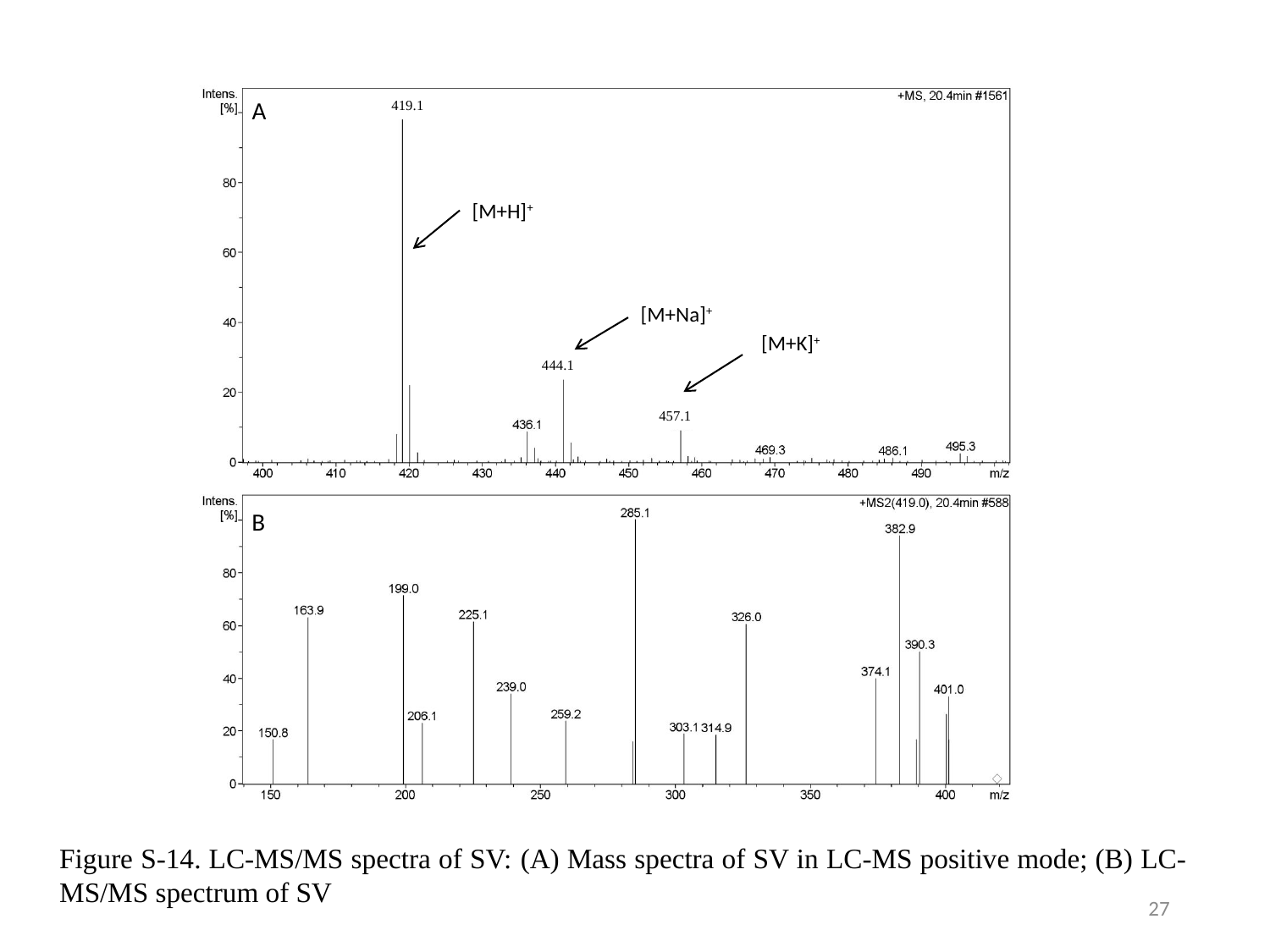

A
419.1
[M+H]+
[M+Na]+
[M+K]+
444.1
457.1
B
Figure S-14. LC-MS/MS spectra of SV: (A) Mass spectra of SV in LC-MS positive mode; (B) LC-MS/MS spectrum of SV
27

## Slide 28
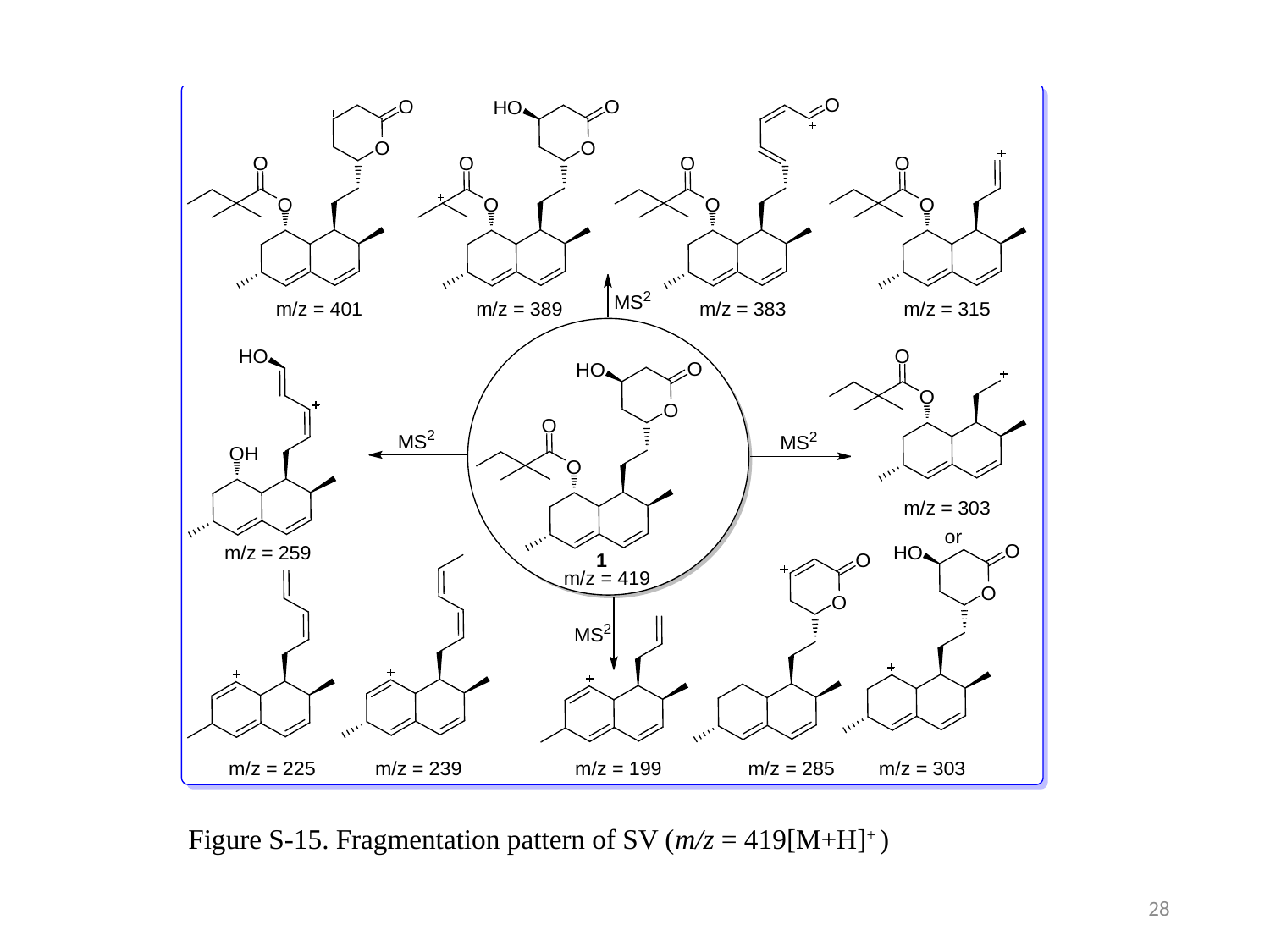

Figure S-15. Fragmentation pattern of SV (m/z = 419[M+H]+ )
28

## Slide 29
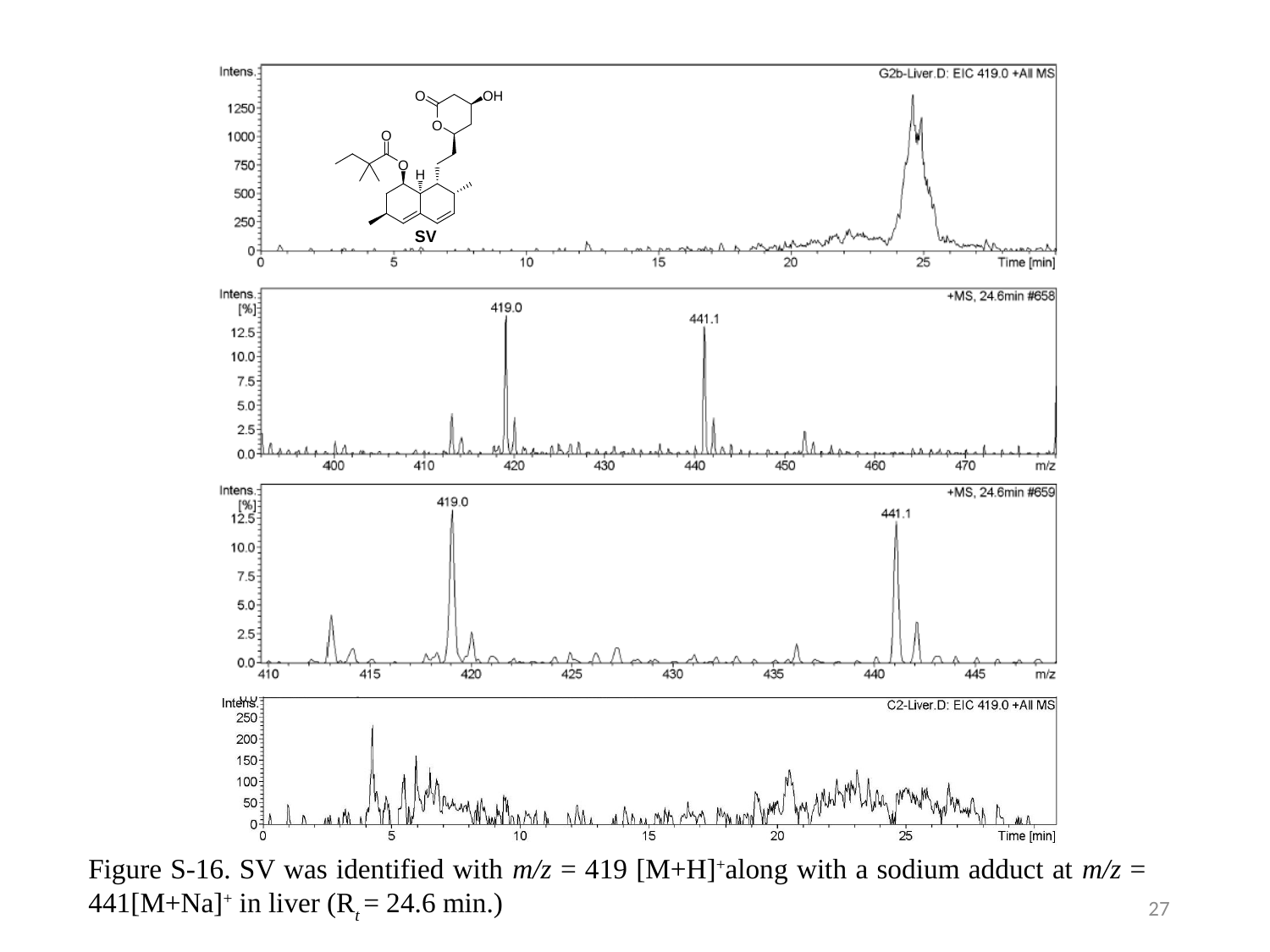

Figure S-16. SV was identified with m/z = 419 [M+H]+along with a sodium adduct at m/z = 441[M+Na]+ in liver (Rt = 24.6 min.)
27

## Slide 30
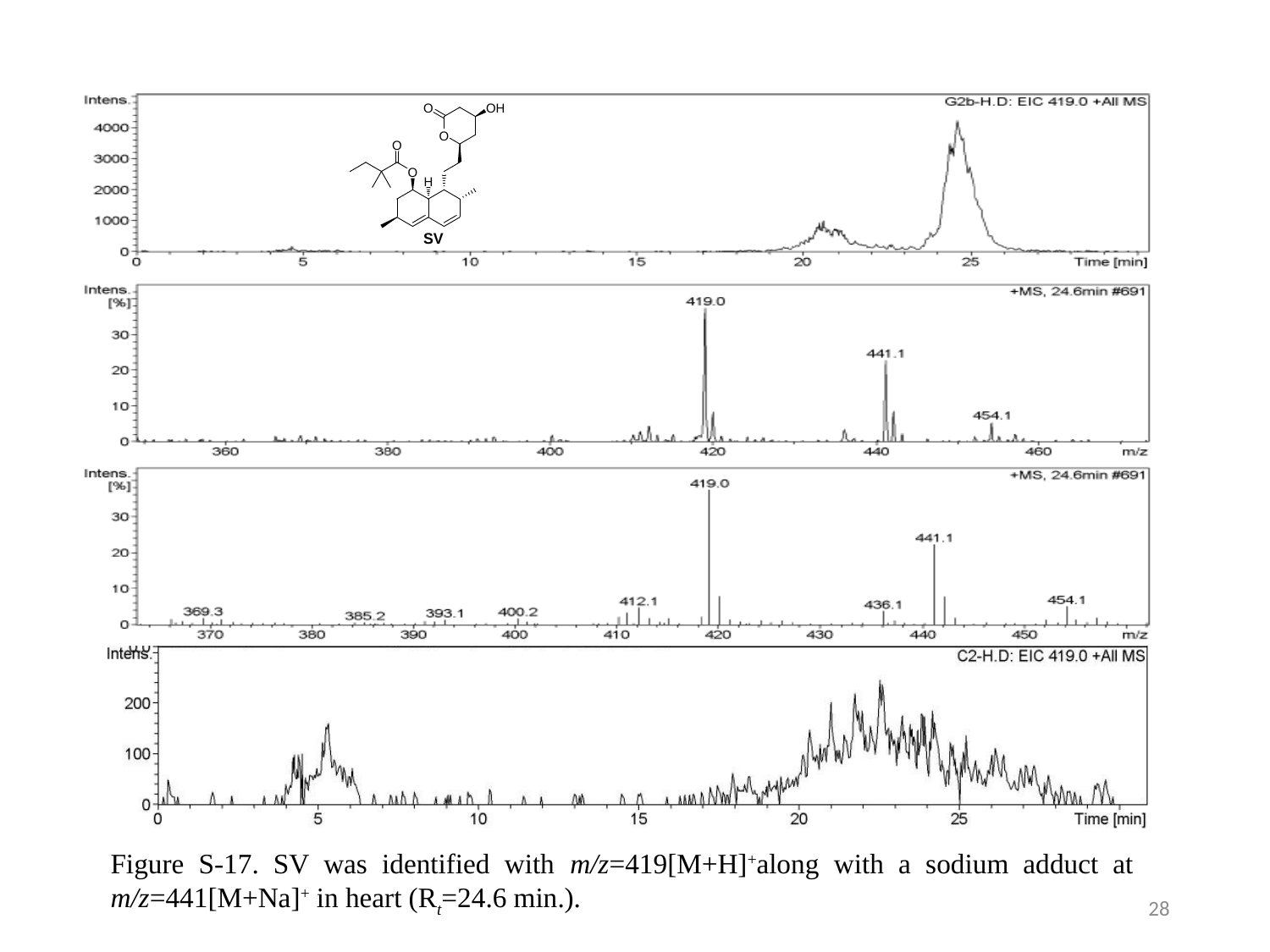

Figure S-17. SV was identified with m/z=419[M+H]+along with a sodium adduct at m/z=441[M+Na]+ in heart (Rt=24.6 min.).
28

## Slide 31
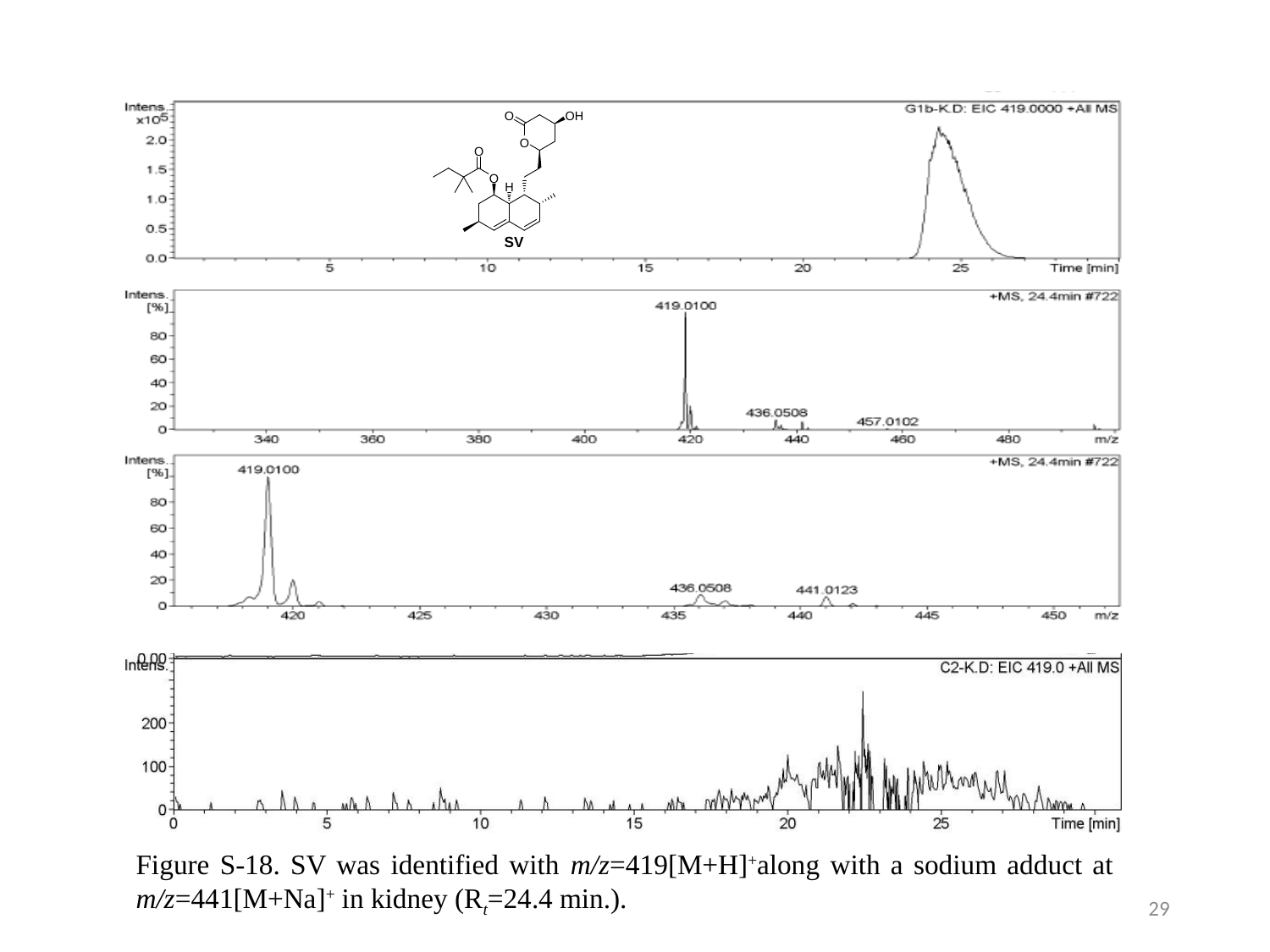

Figure S-18. SV was identified with m/z=419[M+H]+along with a sodium adduct at m/z=441[M+Na]+ in kidney (Rt=24.4 min.).
29

## Slide 32
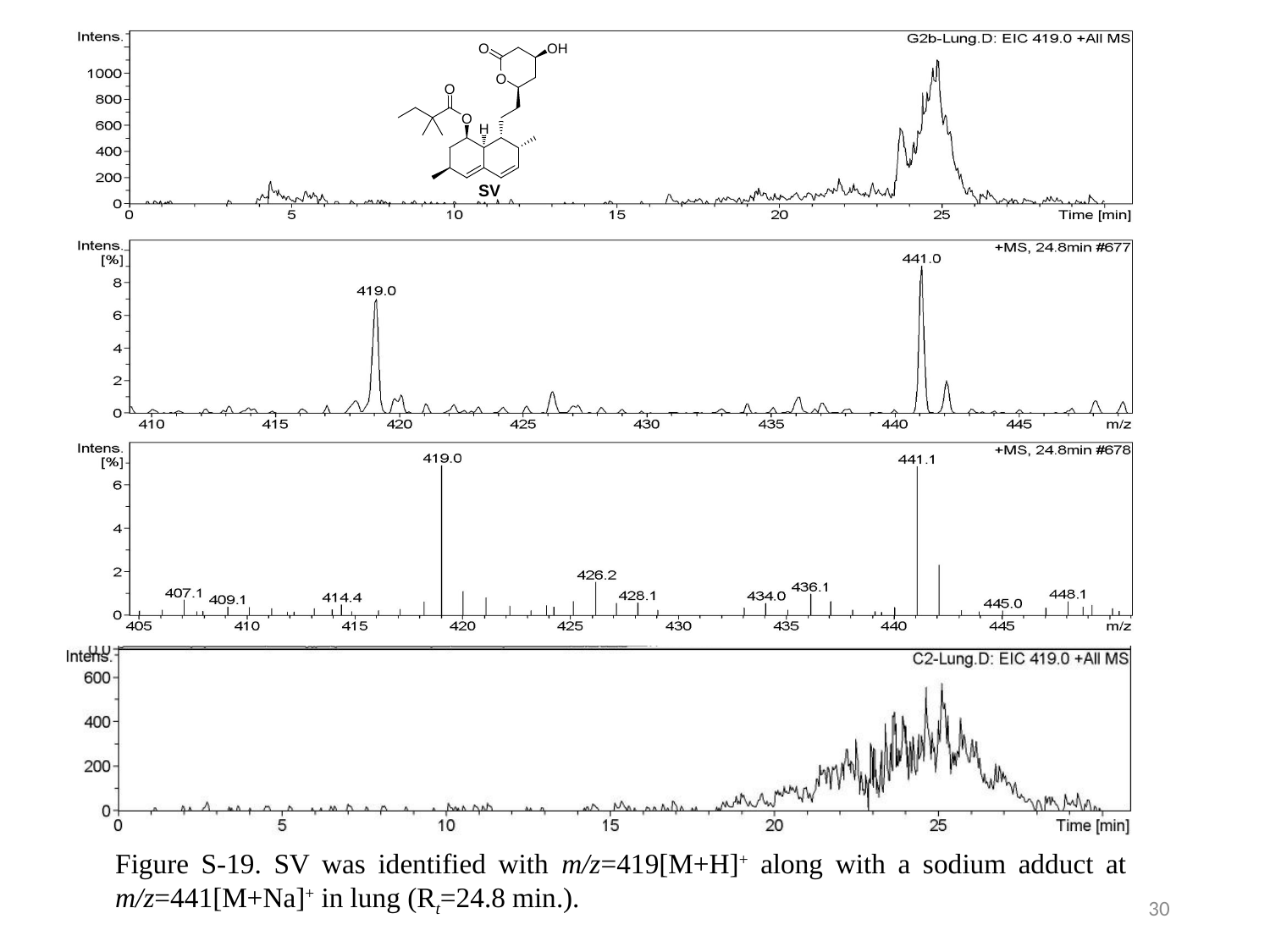

Figure S-19. SV was identified with m/z=419[M+H]+ along with a sodium adduct at m/z=441[M+Na]+ in lung (Rt=24.8 min.).
30

## Slide 33
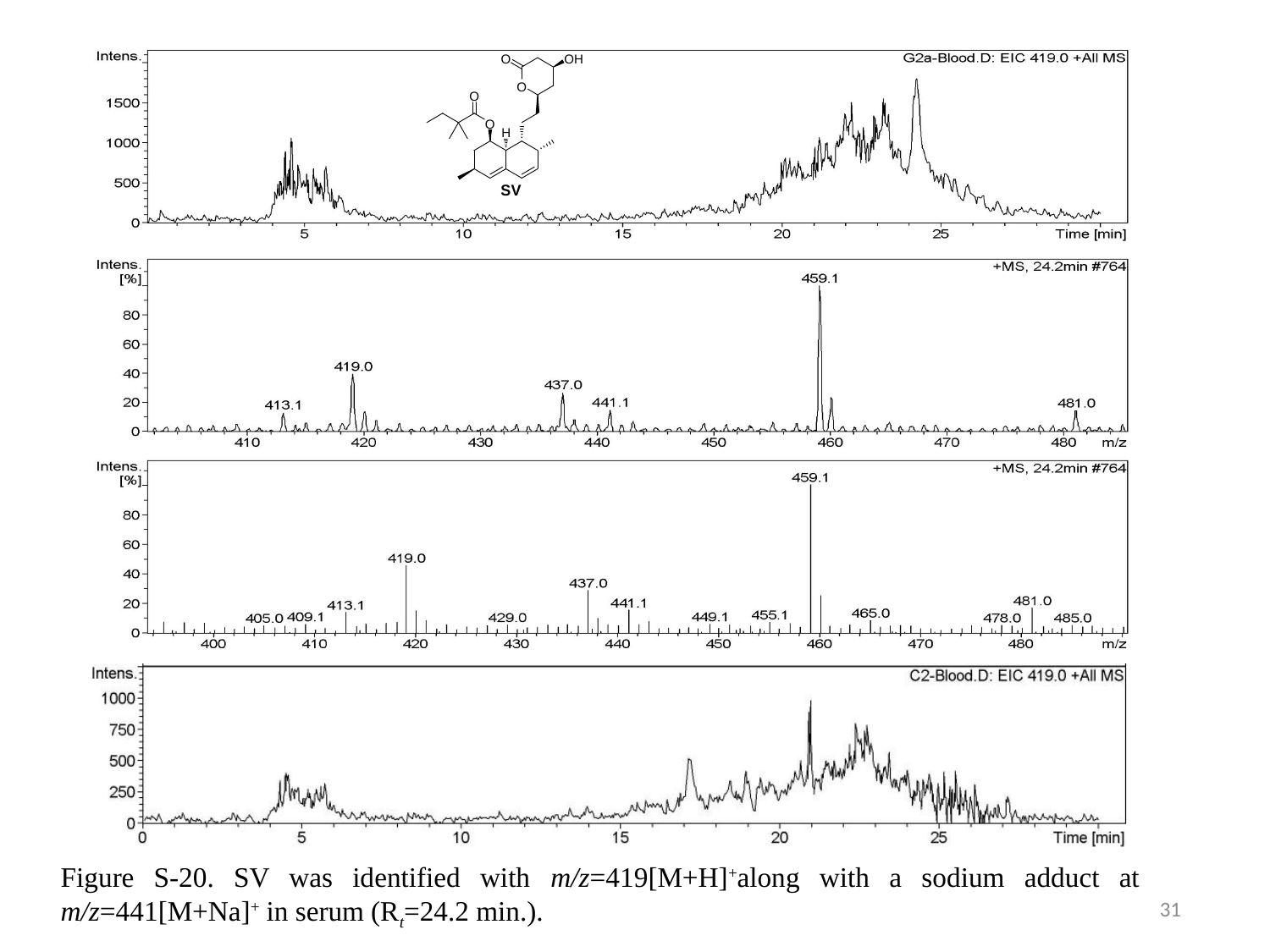

Figure S-20. SV was identified with m/z=419[M+H]+along with a sodium adduct at m/z=441[M+Na]+ in serum (Rt=24.2 min.).
31

## Slide 34
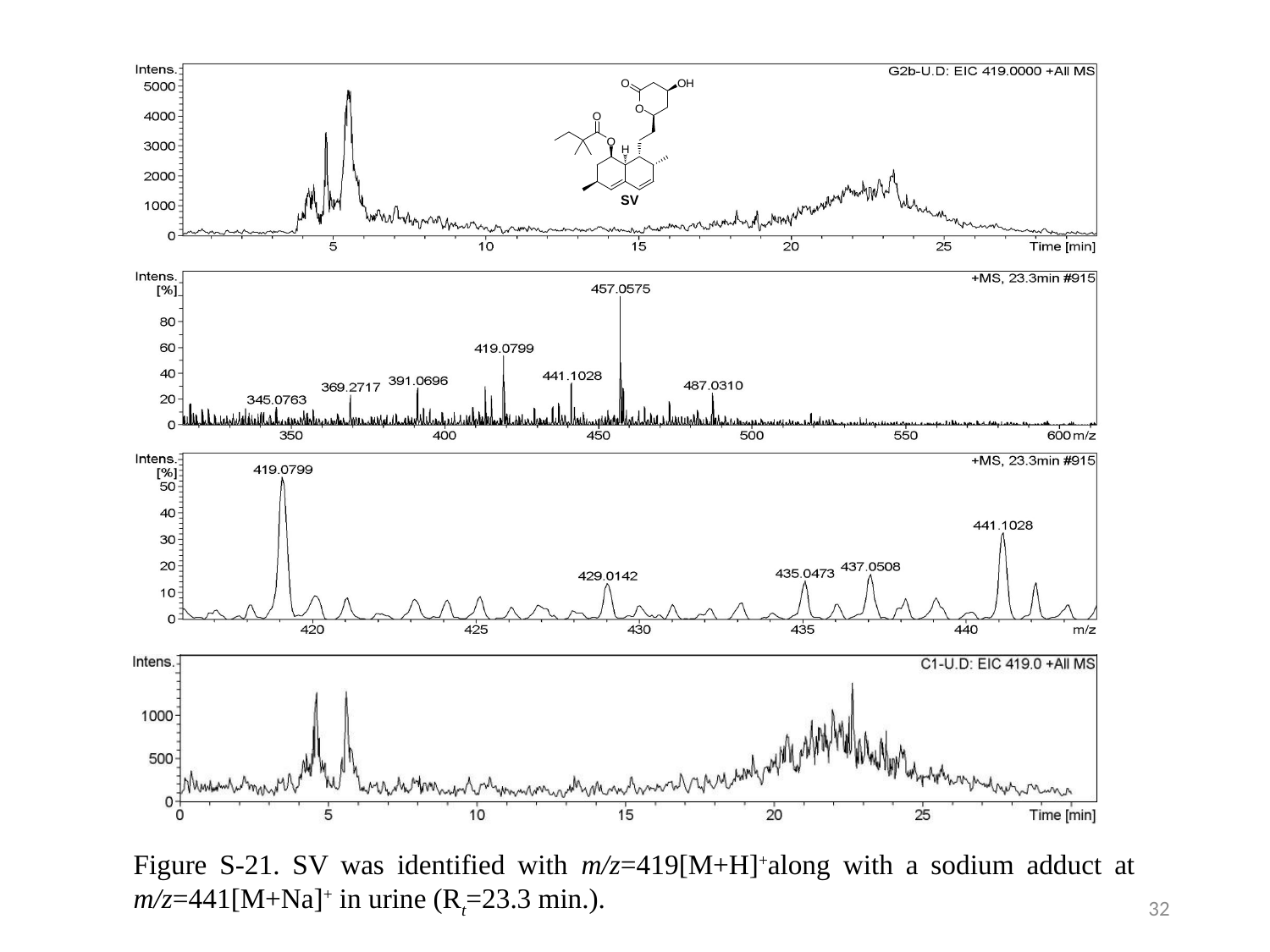

Figure S-21. SV was identified with m/z=419[M+H]+along with a sodium adduct at m/z=441[M+Na]+ in urine (Rt=23.3 min.).
32

## Slide 35
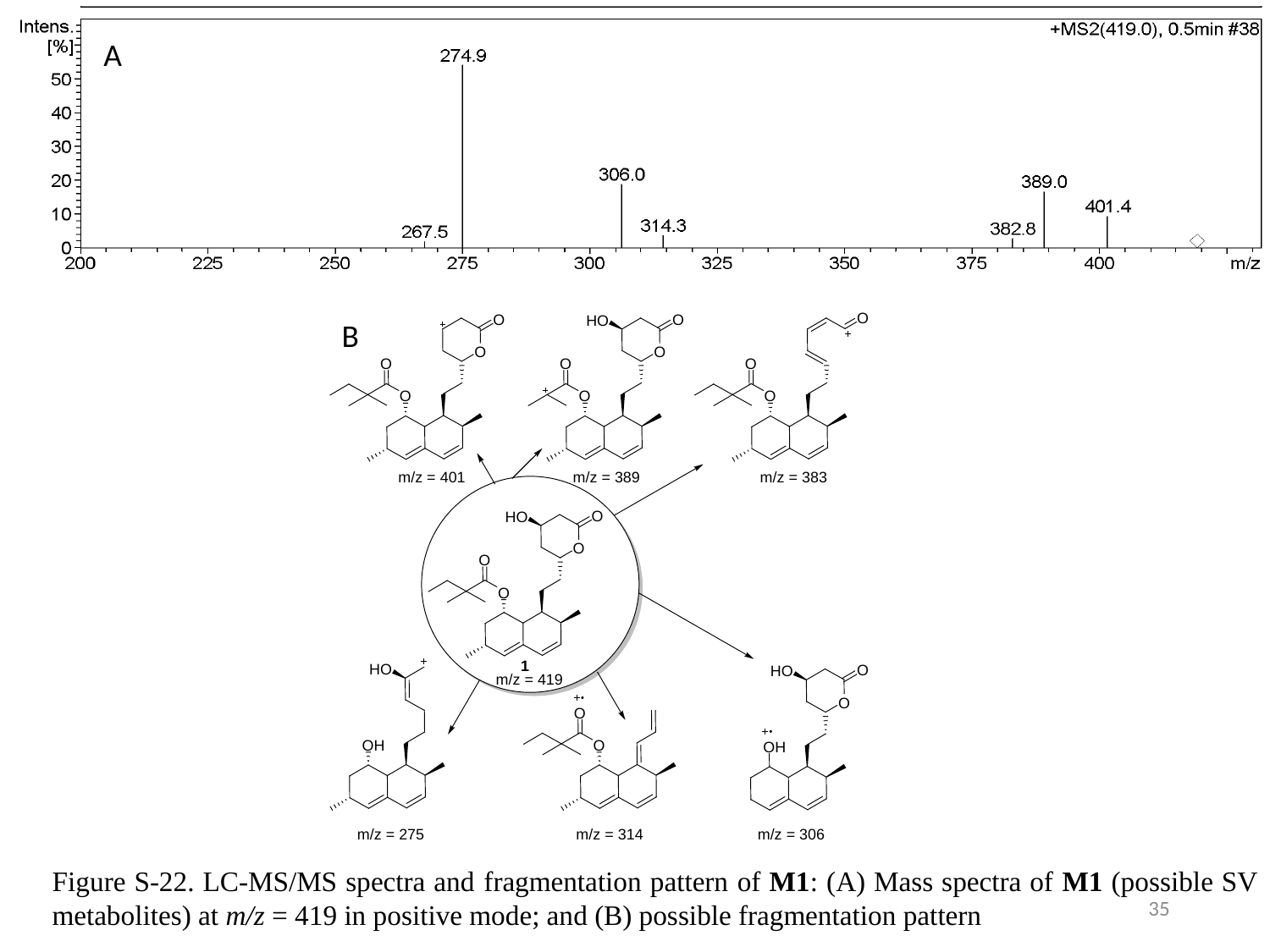

A
B
Figure S-22. LC-MS/MS spectra and fragmentation pattern of M1: (A) Mass spectra of M1 (possible SV metabolites) at m/z = 419 in positive mode; and (B) possible fragmentation pattern
35

## Slide 36
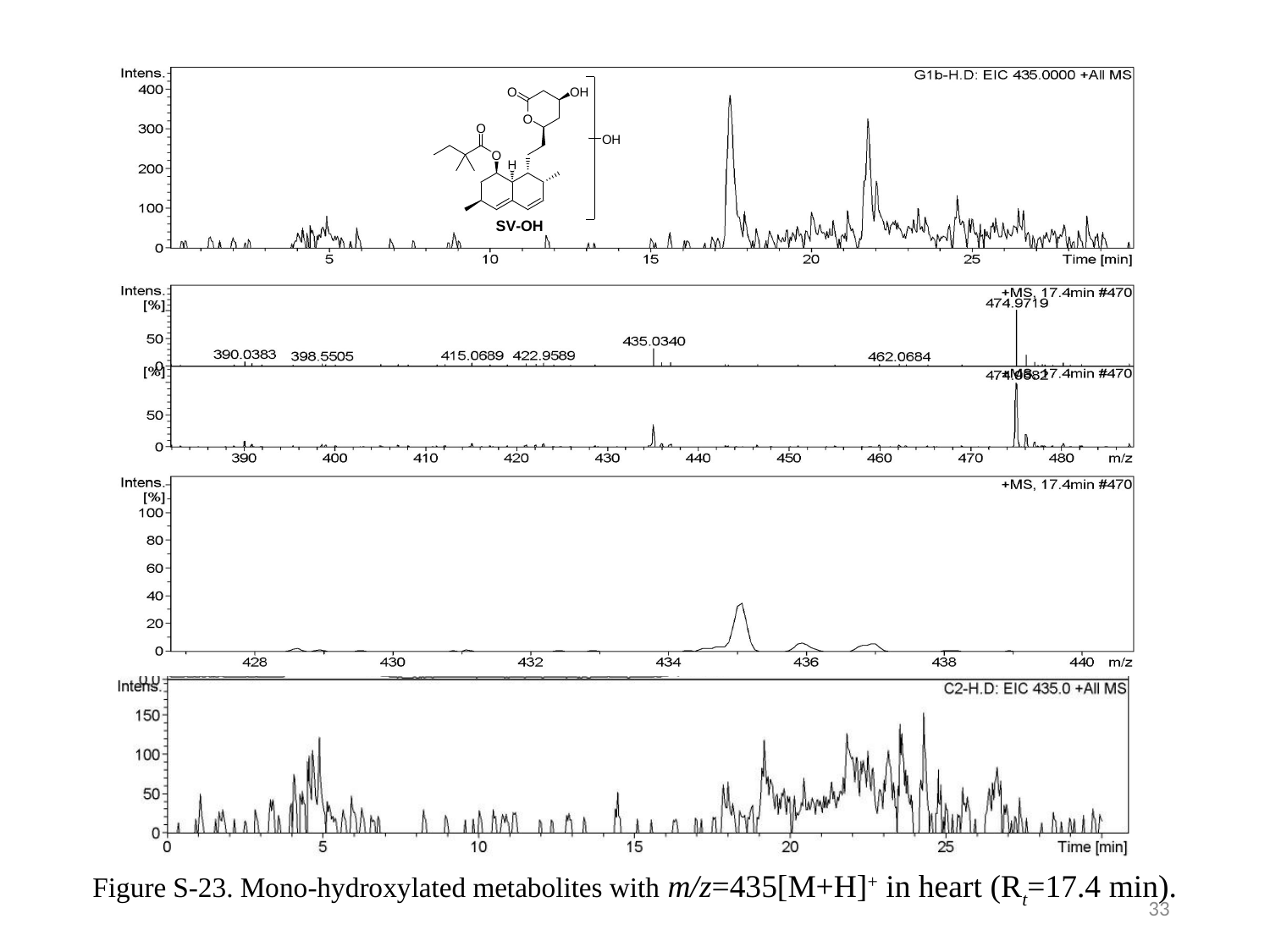

Figure S-23. Mono-hydroxylated metabolites with m/z=435[M+H]+ in heart (Rt=17.4 min).
33

## Slide 37
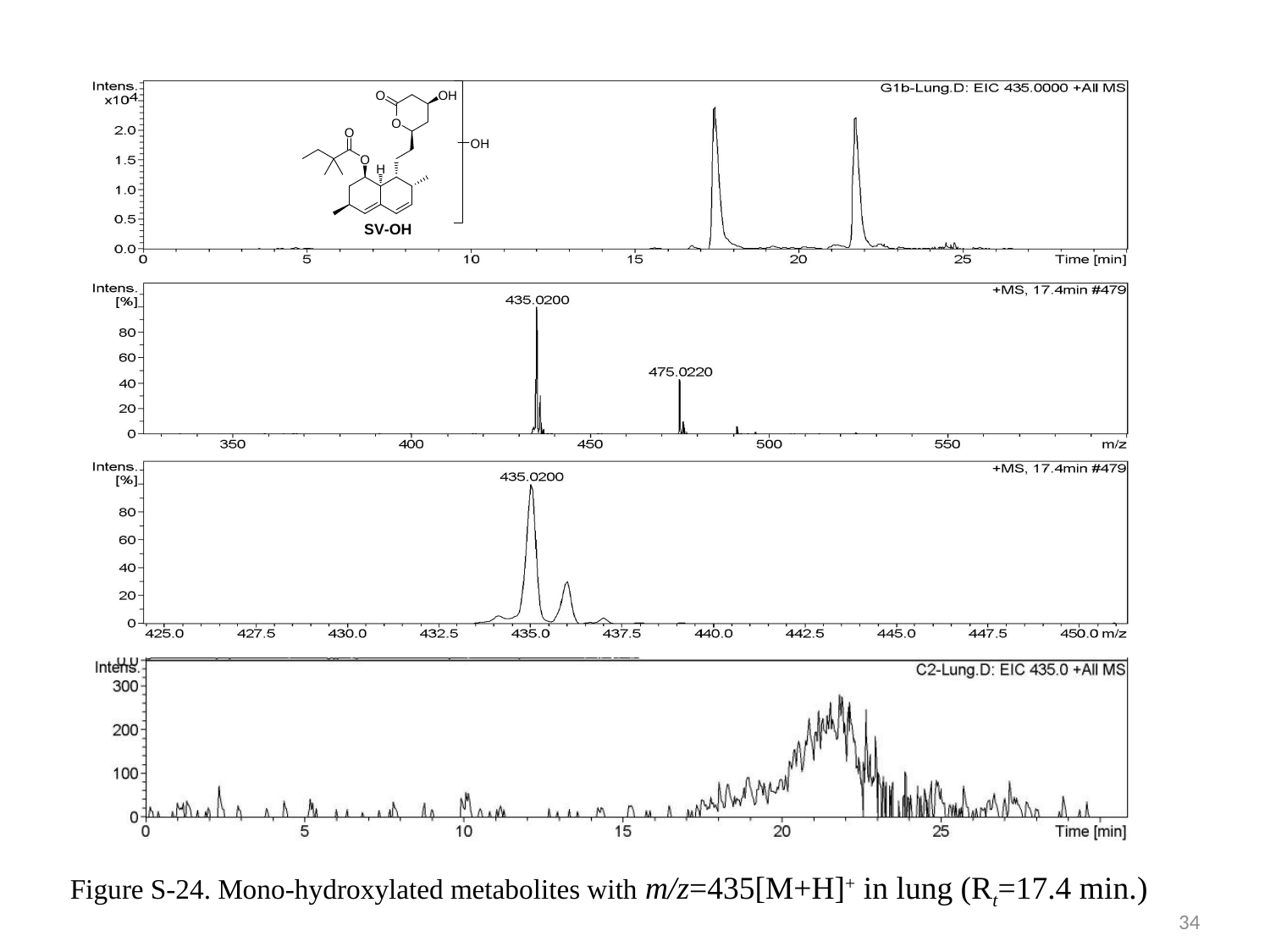

Figure S-24. Mono-hydroxylated metabolites with m/z=435[M+H]+ in lung (Rt=17.4 min.)
34

## Slide 38
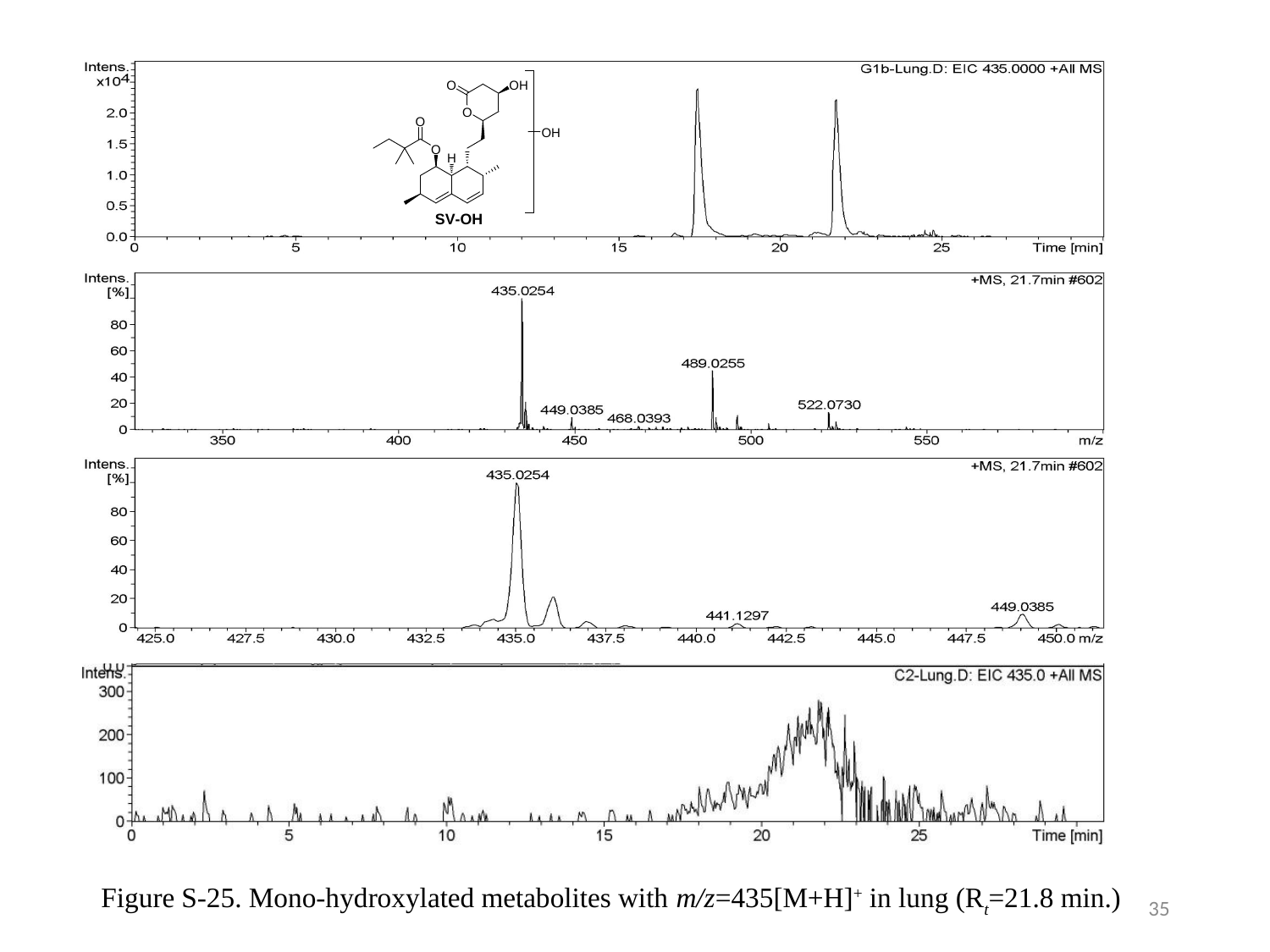

Figure S-25. Mono-hydroxylated metabolites with m/z=435[M+H]+ in lung (Rt=21.8 min.)
35

## Slide 39
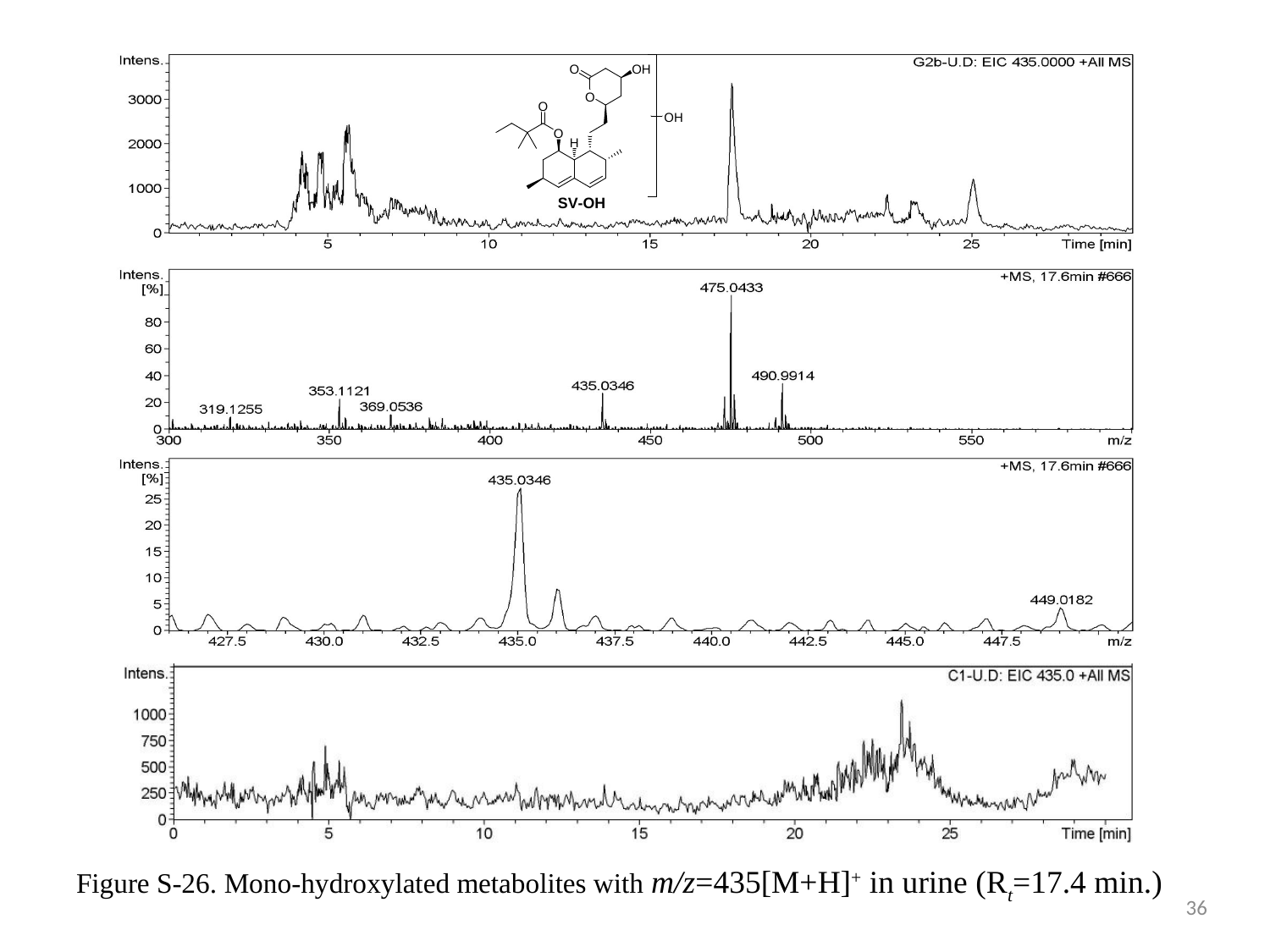

Figure S-26. Mono-hydroxylated metabolites with m/z=435[M+H]+ in urine (Rt=17.4 min.)
36

## Slide 40
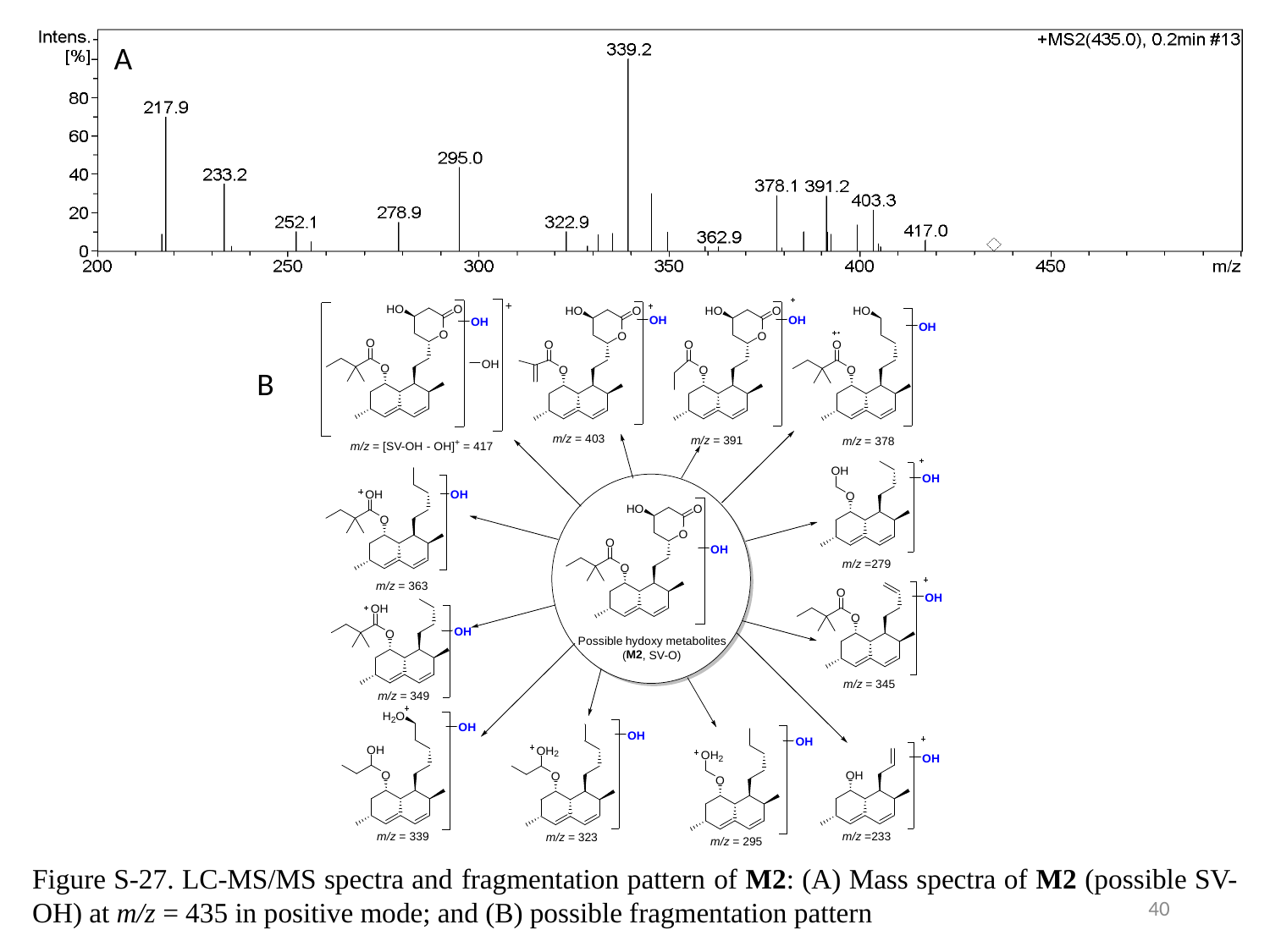

A
B
Figure S-27. LC-MS/MS spectra and fragmentation pattern of M2: (A) Mass spectra of M2 (possible SV-OH) at m/z = 435 in positive mode; and (B) possible fragmentation pattern
40

## Slide 41
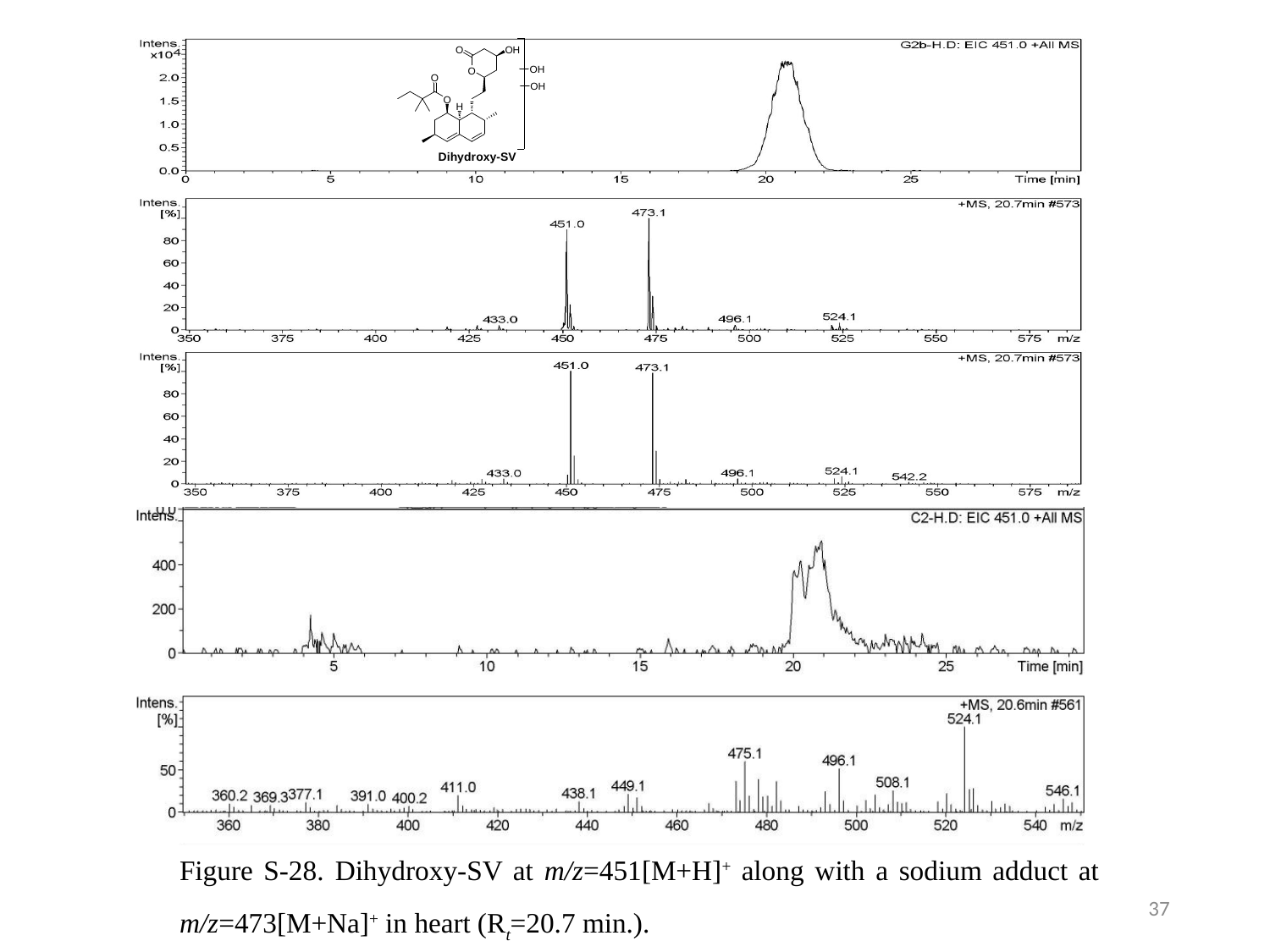

Figure S-28. Dihydroxy-SV at m/z=451[M+H]+ along with a sodium adduct at m/z=473[M+Na]+ in heart (Rt=20.7 min.).
37

## Slide 42
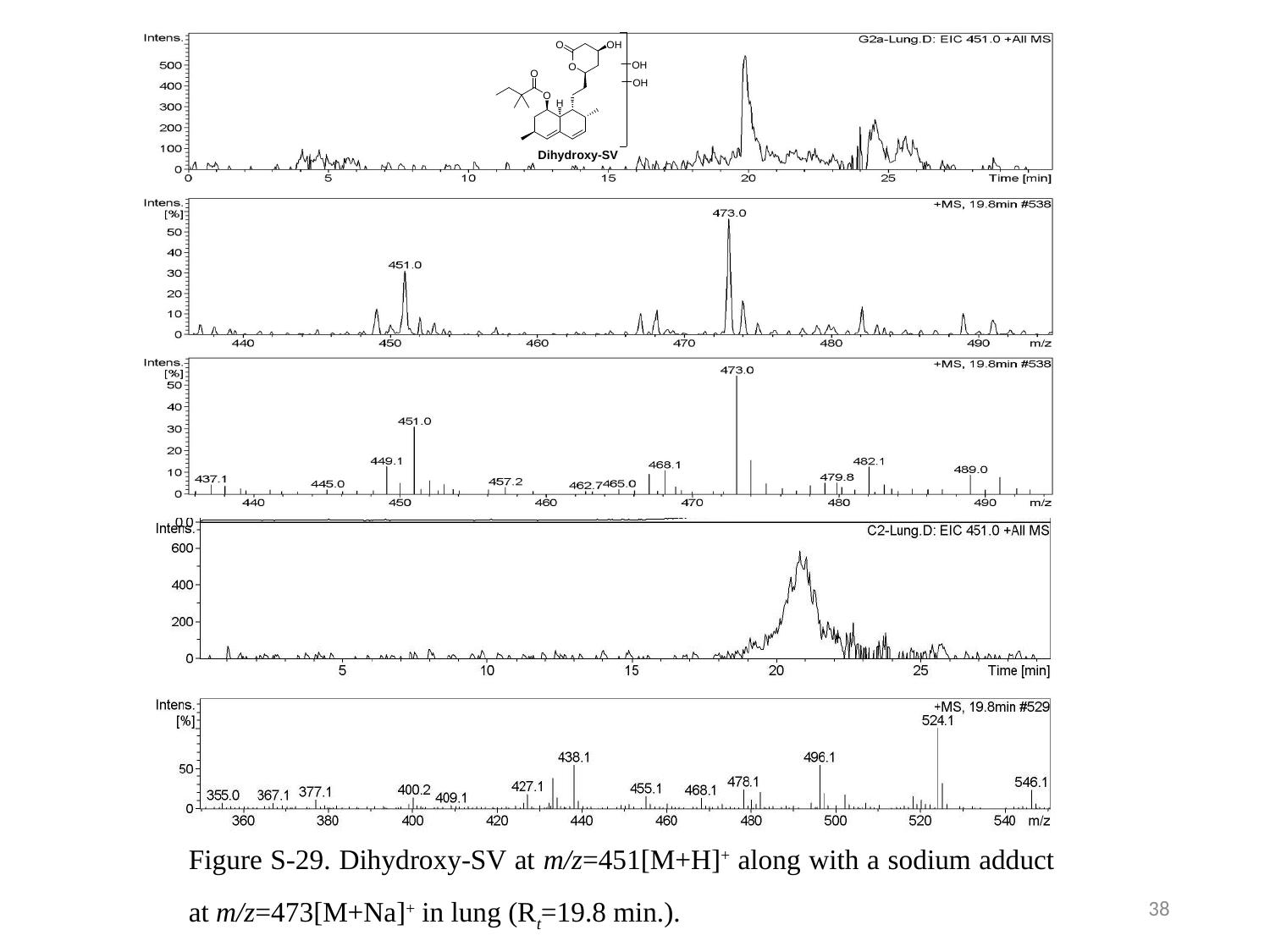

Figure S-29. Dihydroxy-SV at m/z=451[M+H]+ along with a sodium adduct at m/z=473[M+Na]+ in lung (Rt=19.8 min.).
38

## Slide 43
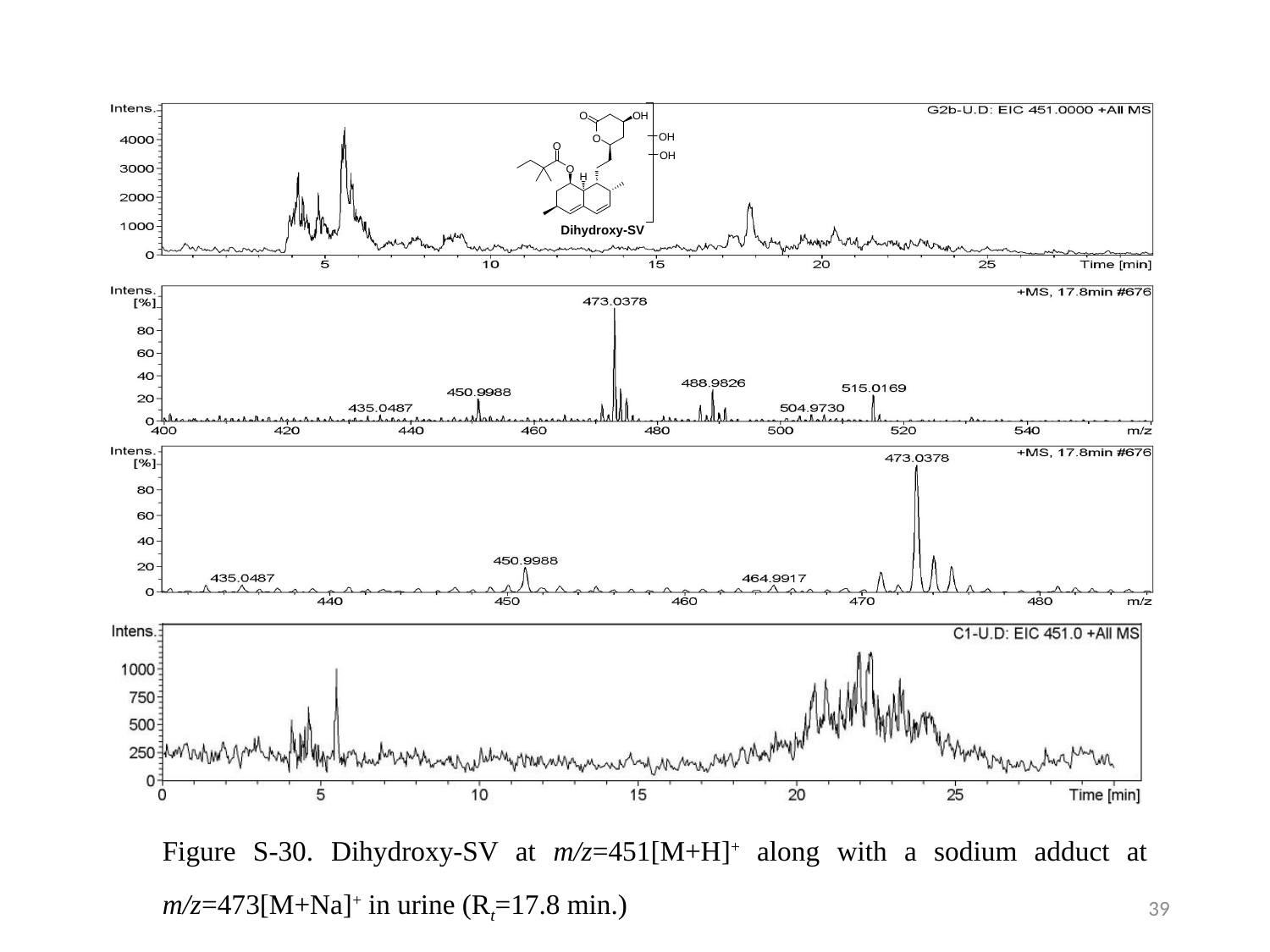

Figure S-30. Dihydroxy-SV at m/z=451[M+H]+ along with a sodium adduct at m/z=473[M+Na]+ in urine (Rt=17.8 min.)
39

## Slide 44
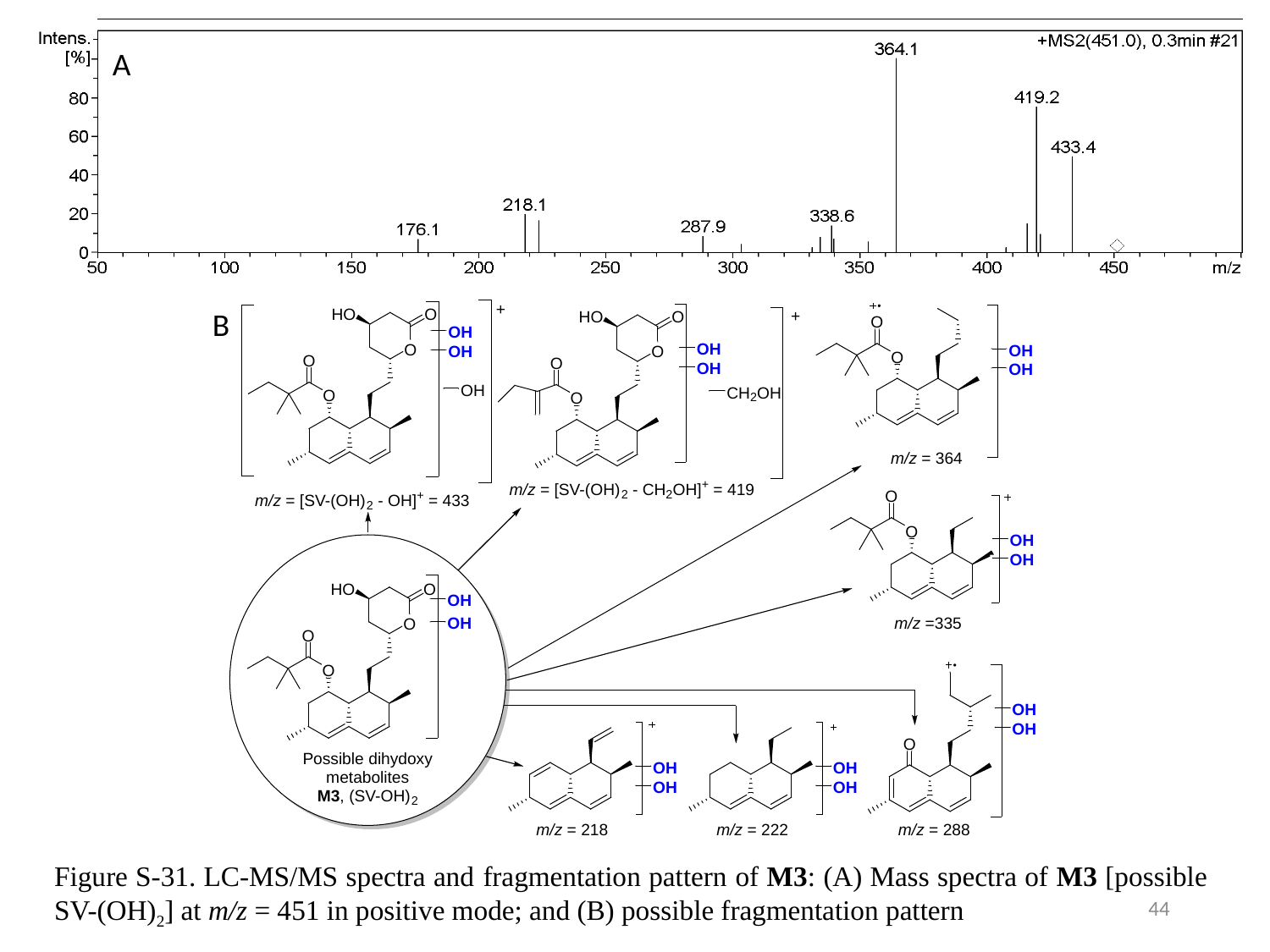

A
B
Figure S-31. LC-MS/MS spectra and fragmentation pattern of M3: (A) Mass spectra of M3 [possible SV-(OH)2] at m/z = 451 in positive mode; and (B) possible fragmentation pattern
44

## Slide 45
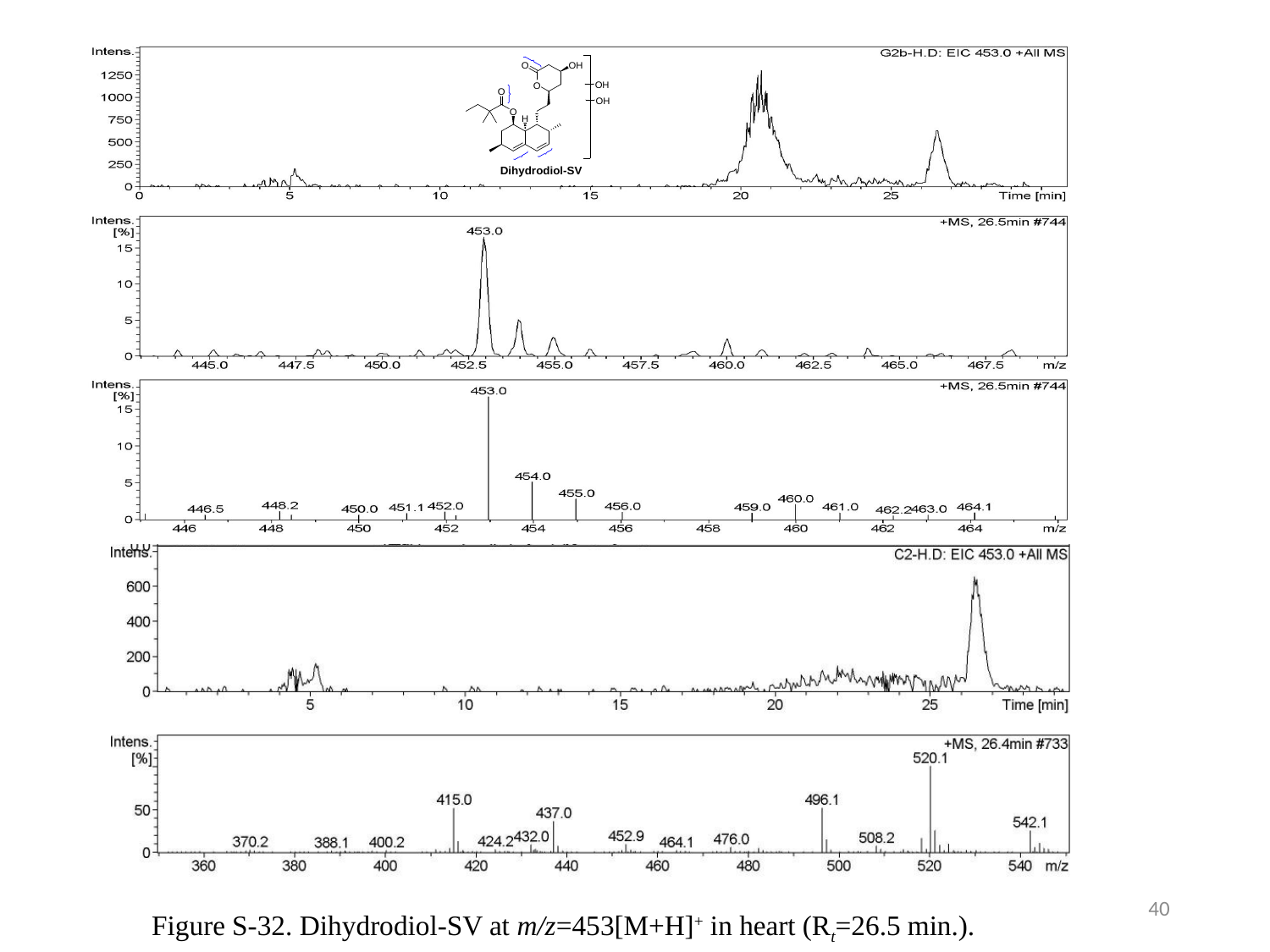

Figure S-32. Dihydrodiol-SV at m/z=453[M+H]+ in heart (Rt=26.5 min.).
40

## Slide 46
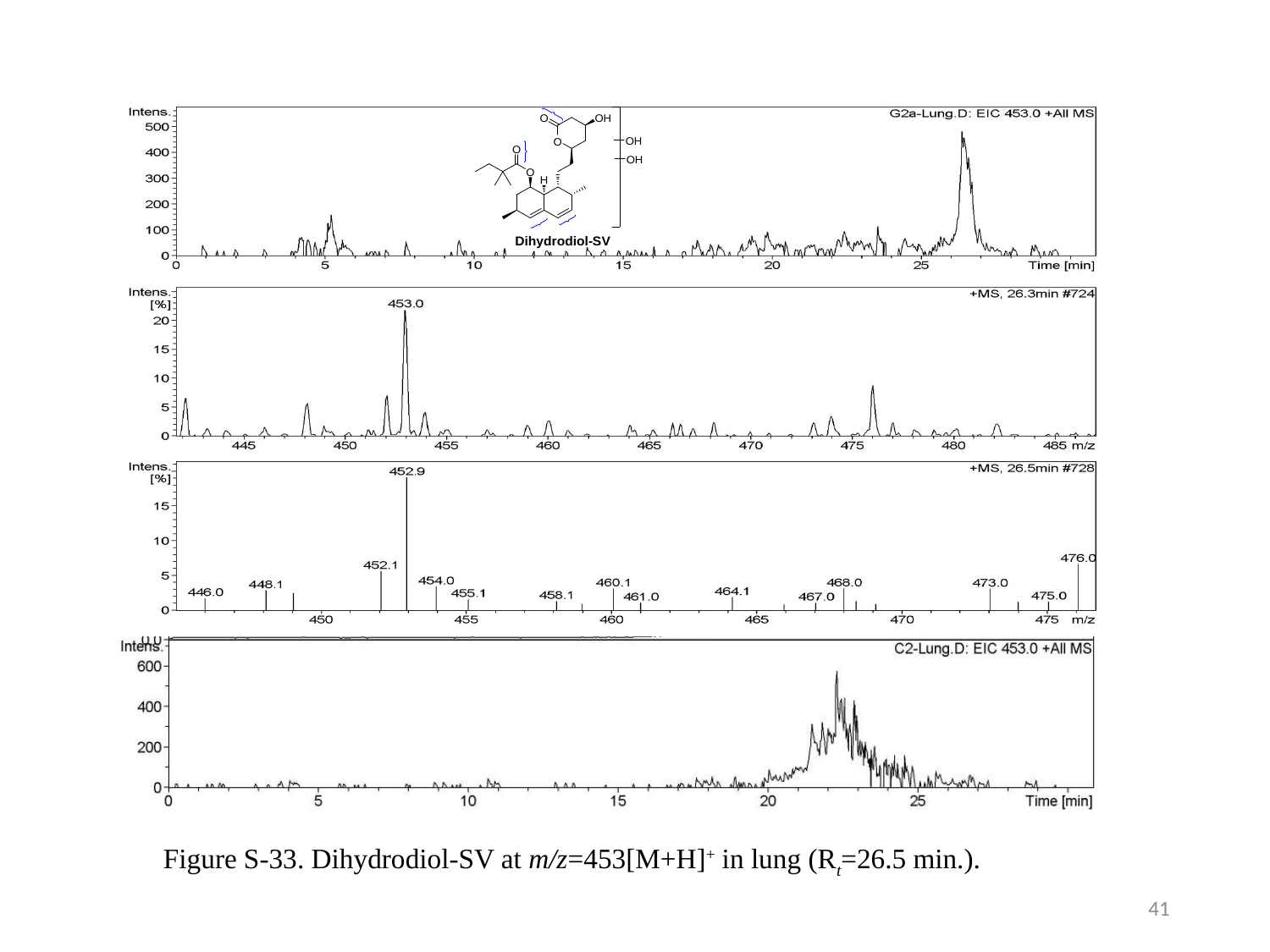

Figure S-33. Dihydrodiol-SV at m/z=453[M+H]+ in lung (Rt=26.5 min.).
41

## Slide 47
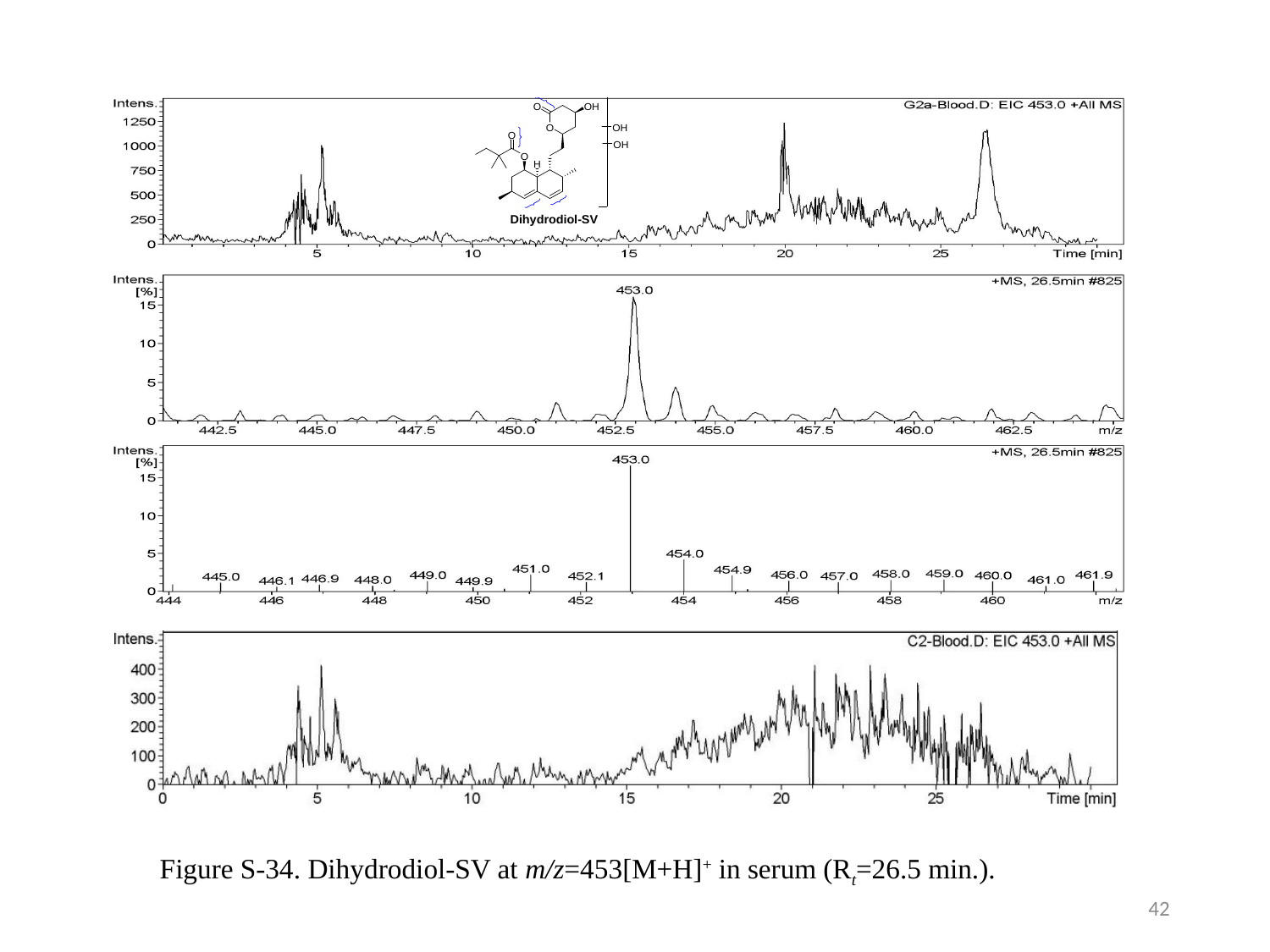

Figure S-34. Dihydrodiol-SV at m/z=453[M+H]+ in serum (Rt=26.5 min.).
42

## Slide 48
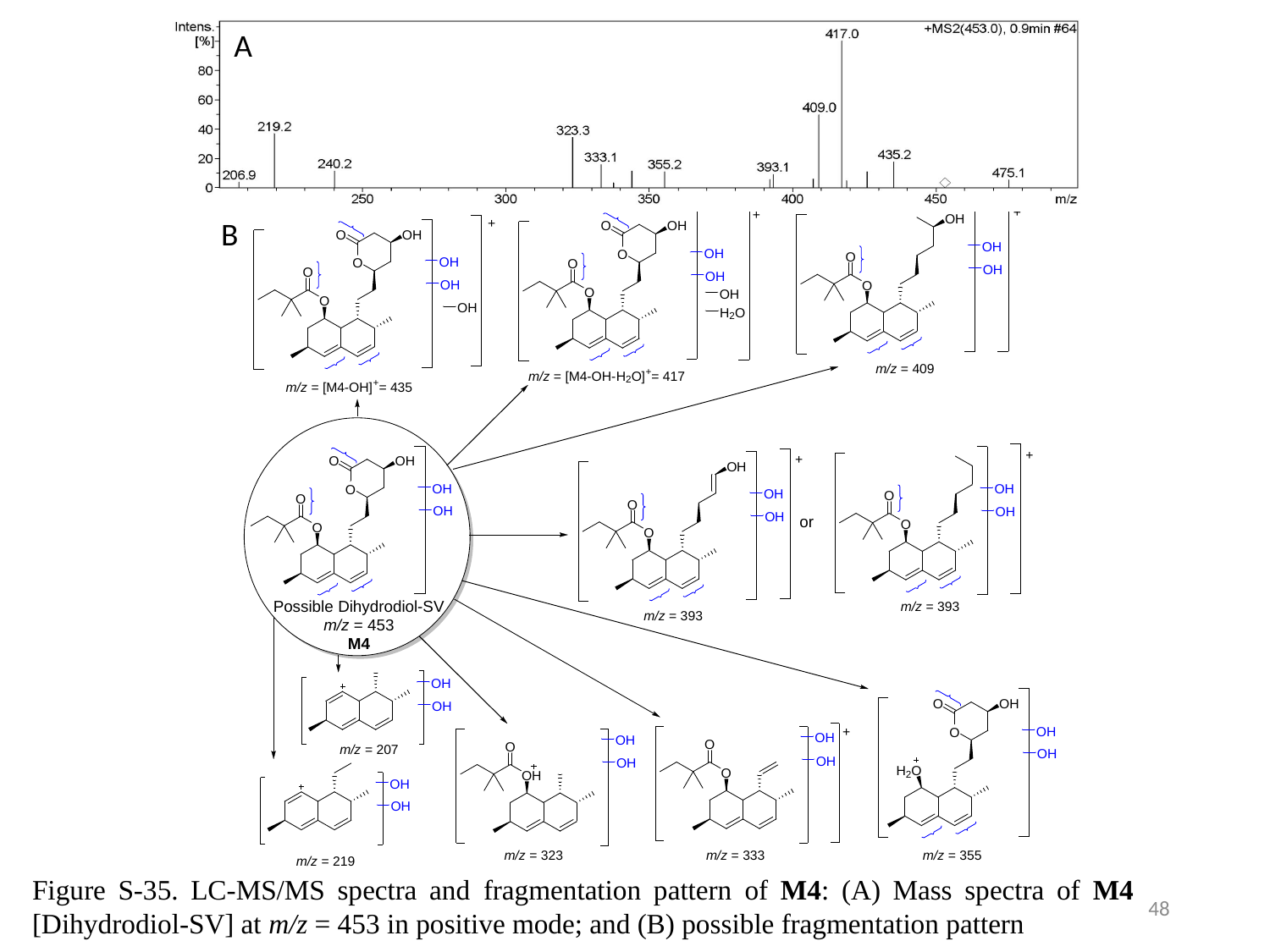

A
B
Figure S-35. LC-MS/MS spectra and fragmentation pattern of M4: (A) Mass spectra of M4 [Dihydrodiol-SV] at m/z = 453 in positive mode; and (B) possible fragmentation pattern
48

## Slide 49
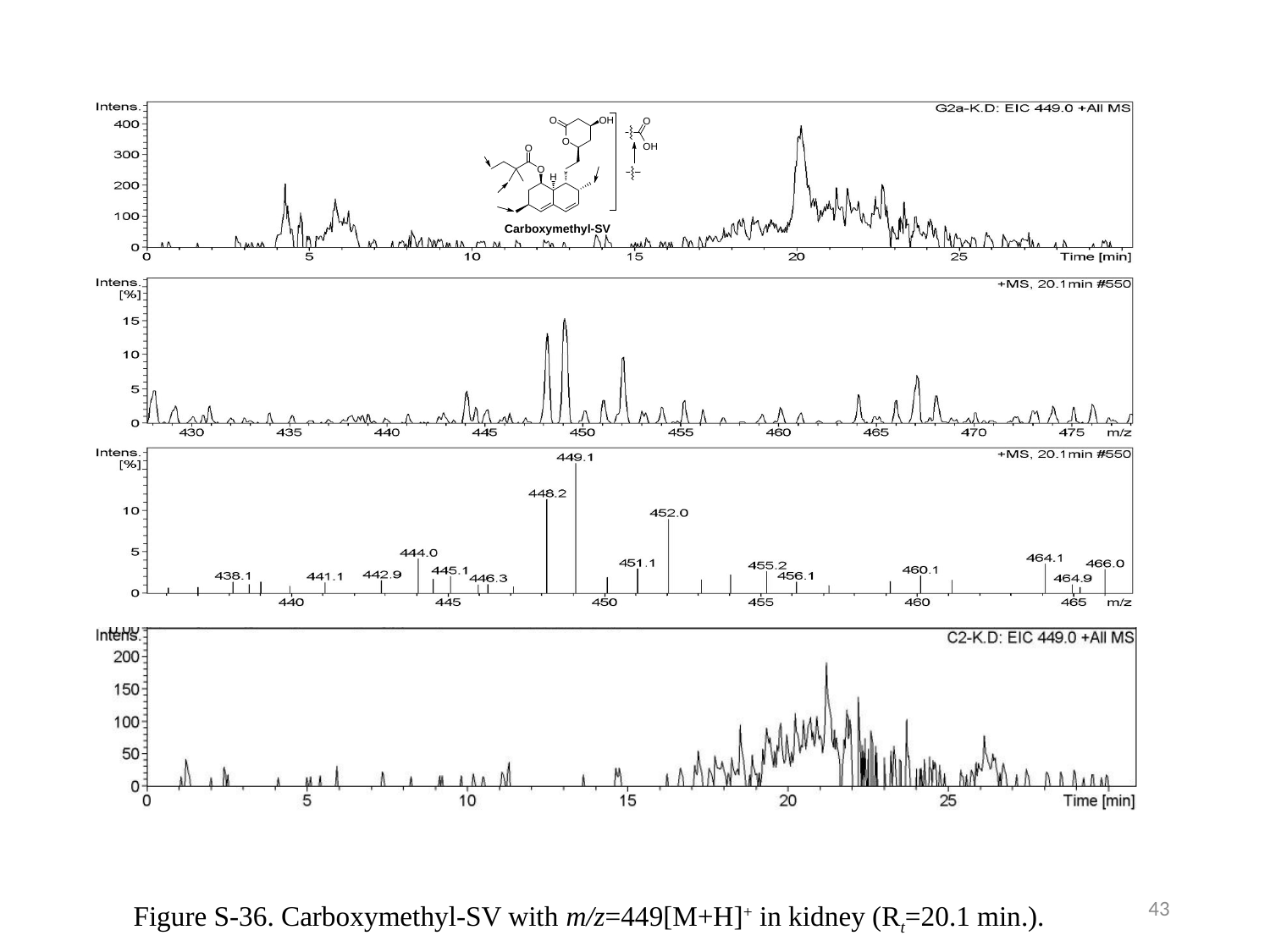

Figure S-36. Carboxymethyl-SV with m/z=449[M+H]+ in kidney (Rt=20.1 min.).
43

## Slide 50
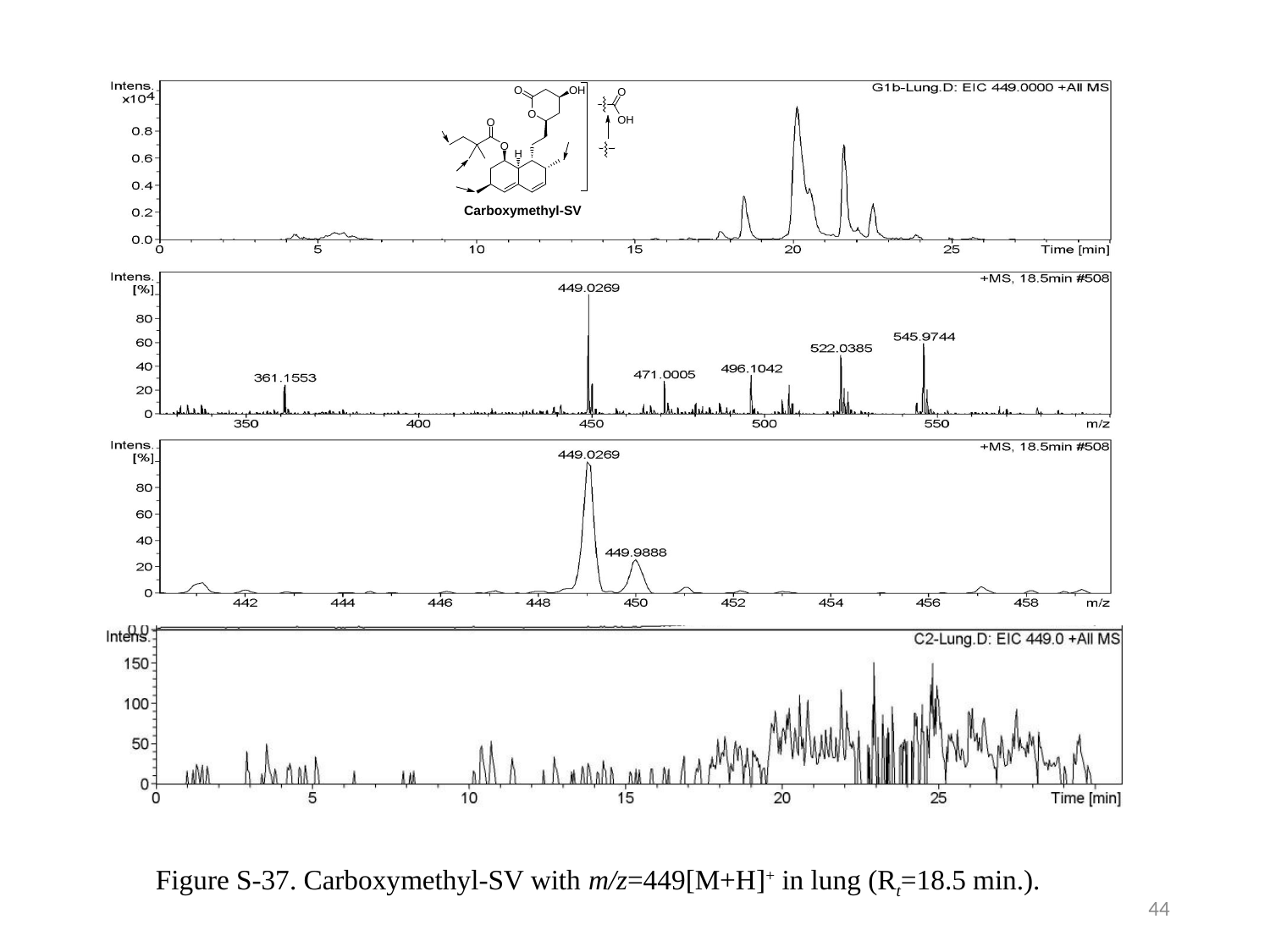

Figure S-37. Carboxymethyl-SV with m/z=449[M+H]+ in lung (Rt=18.5 min.).
44

## Slide 51
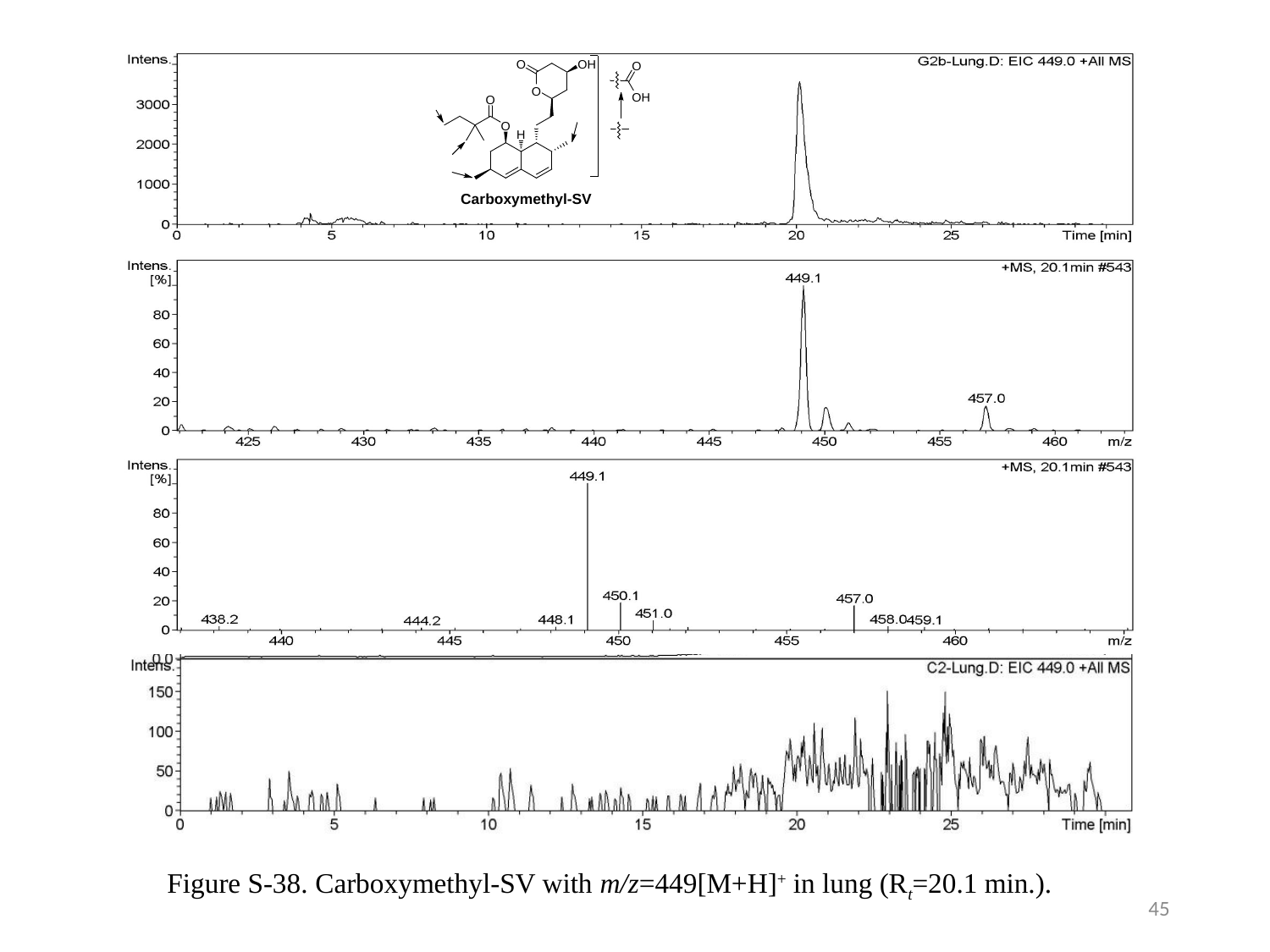

Figure S-38. Carboxymethyl-SV with m/z=449[M+H]+ in lung (Rt=20.1 min.).
45

## Slide 52
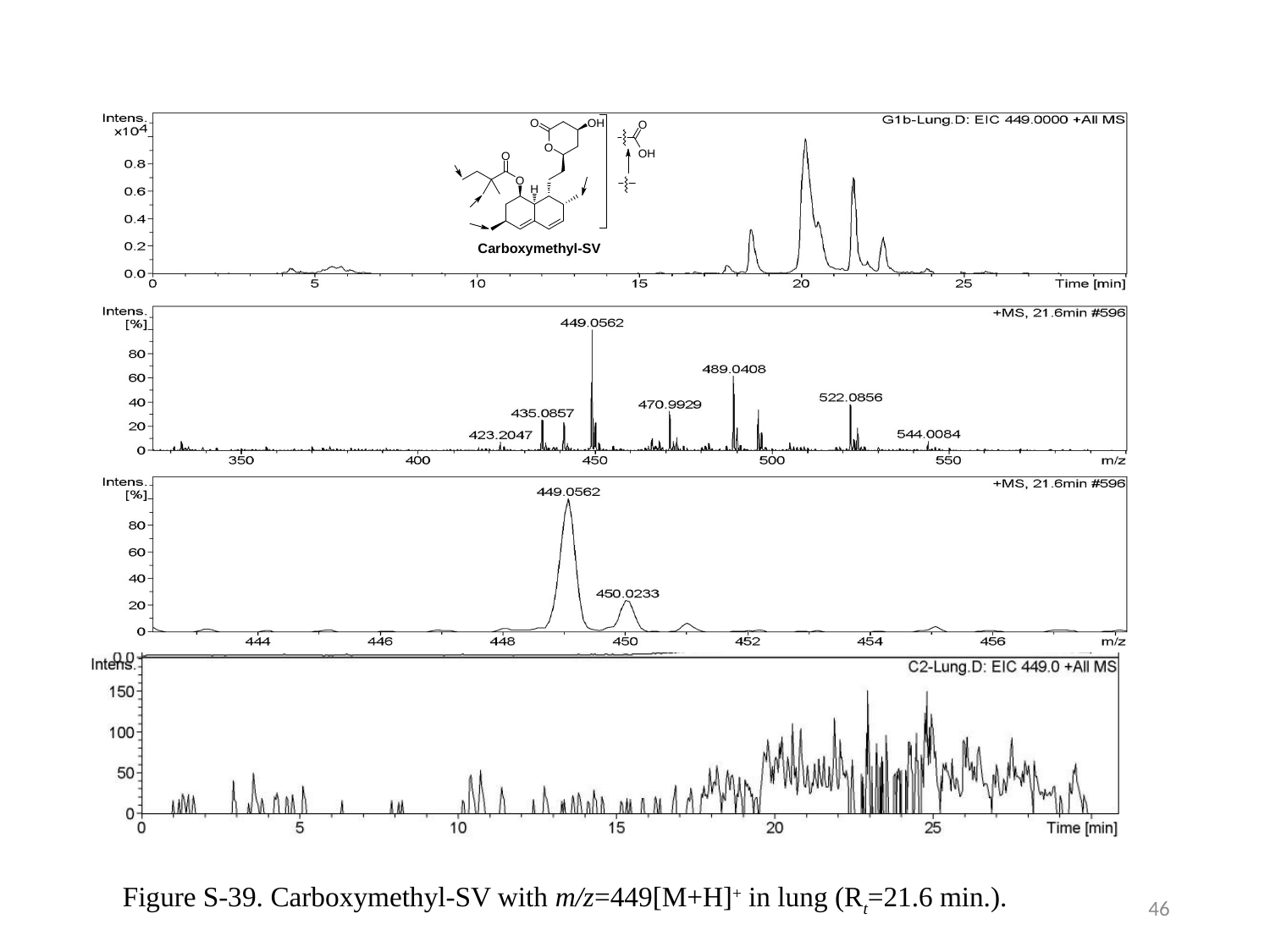

Figure S-39. Carboxymethyl-SV with m/z=449[M+H]+ in lung (Rt=21.6 min.).
46

## Slide 53
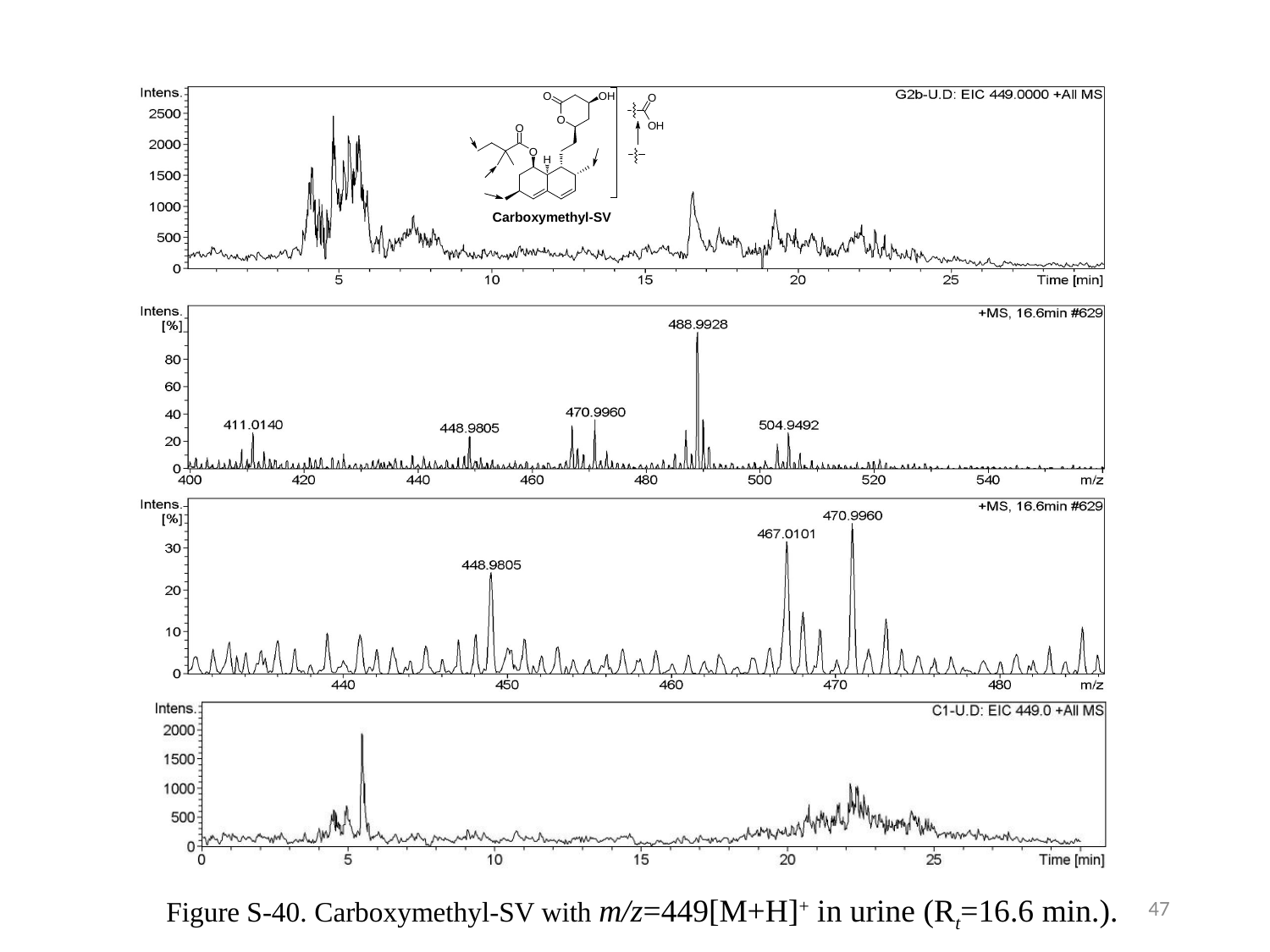

Figure S-40. Carboxymethyl-SV with m/z=449[M+H]+ in urine (Rt=16.6 min.).
47

## Slide 54
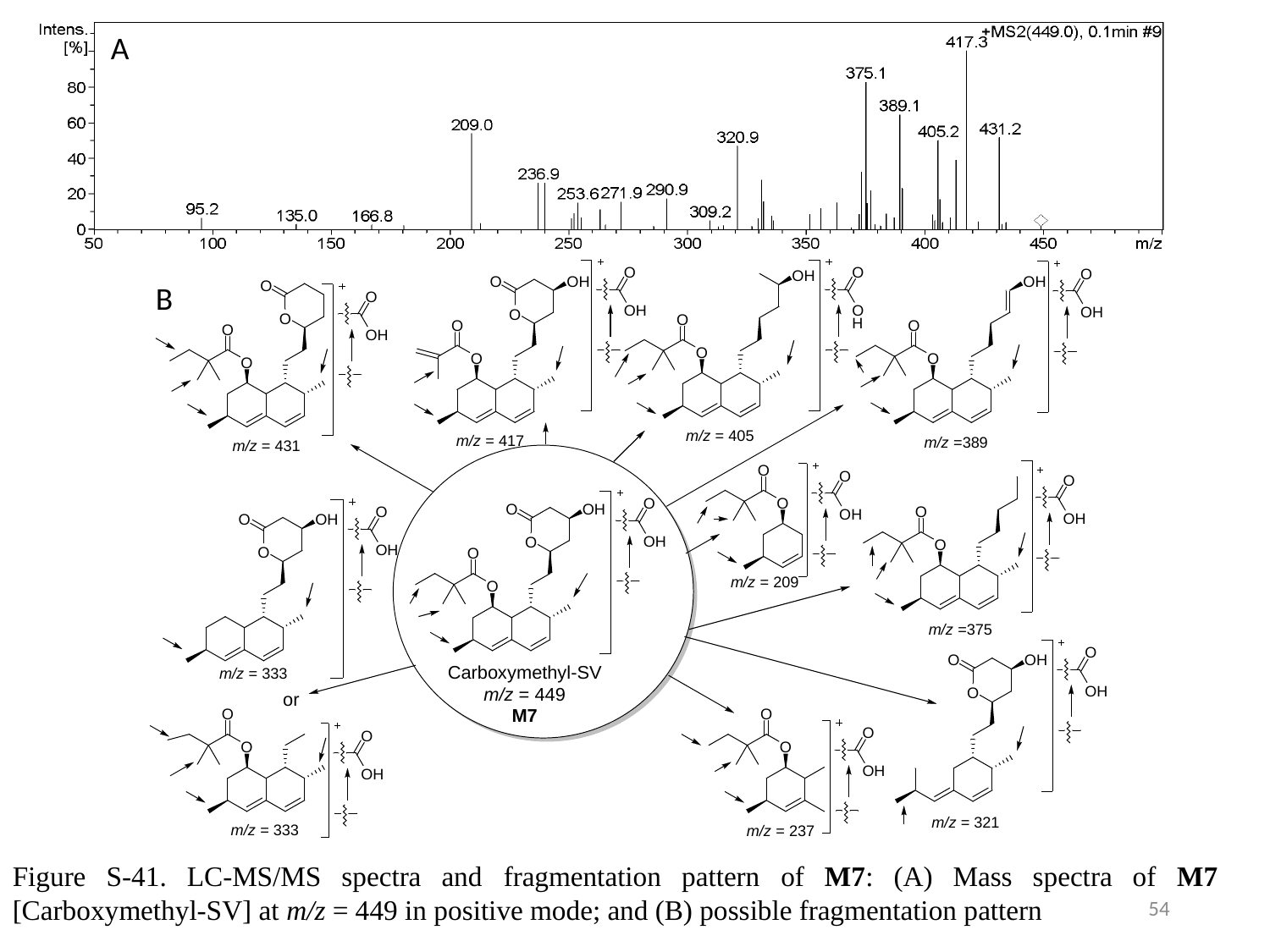

A
A
B
Figure S-41. LC-MS/MS spectra and fragmentation pattern of M7: (A) Mass spectra of M7 [Carboxymethyl-SV] at m/z = 449 in positive mode; and (B) possible fragmentation pattern
54

## Slide 55
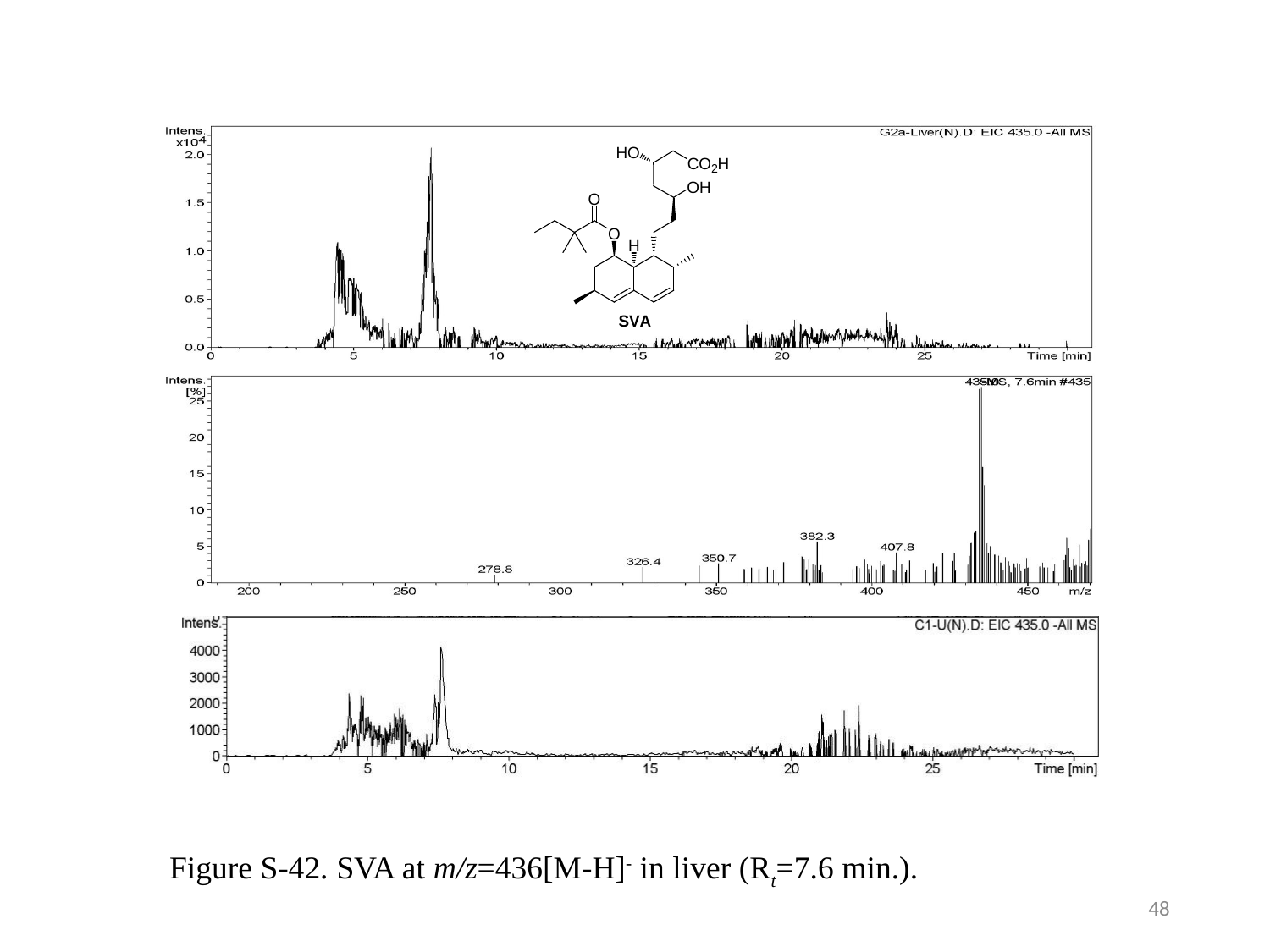

Figure S-42. SVA at m/z=436[M-H]- in liver (Rt=7.6 min.).
48

## Slide 56
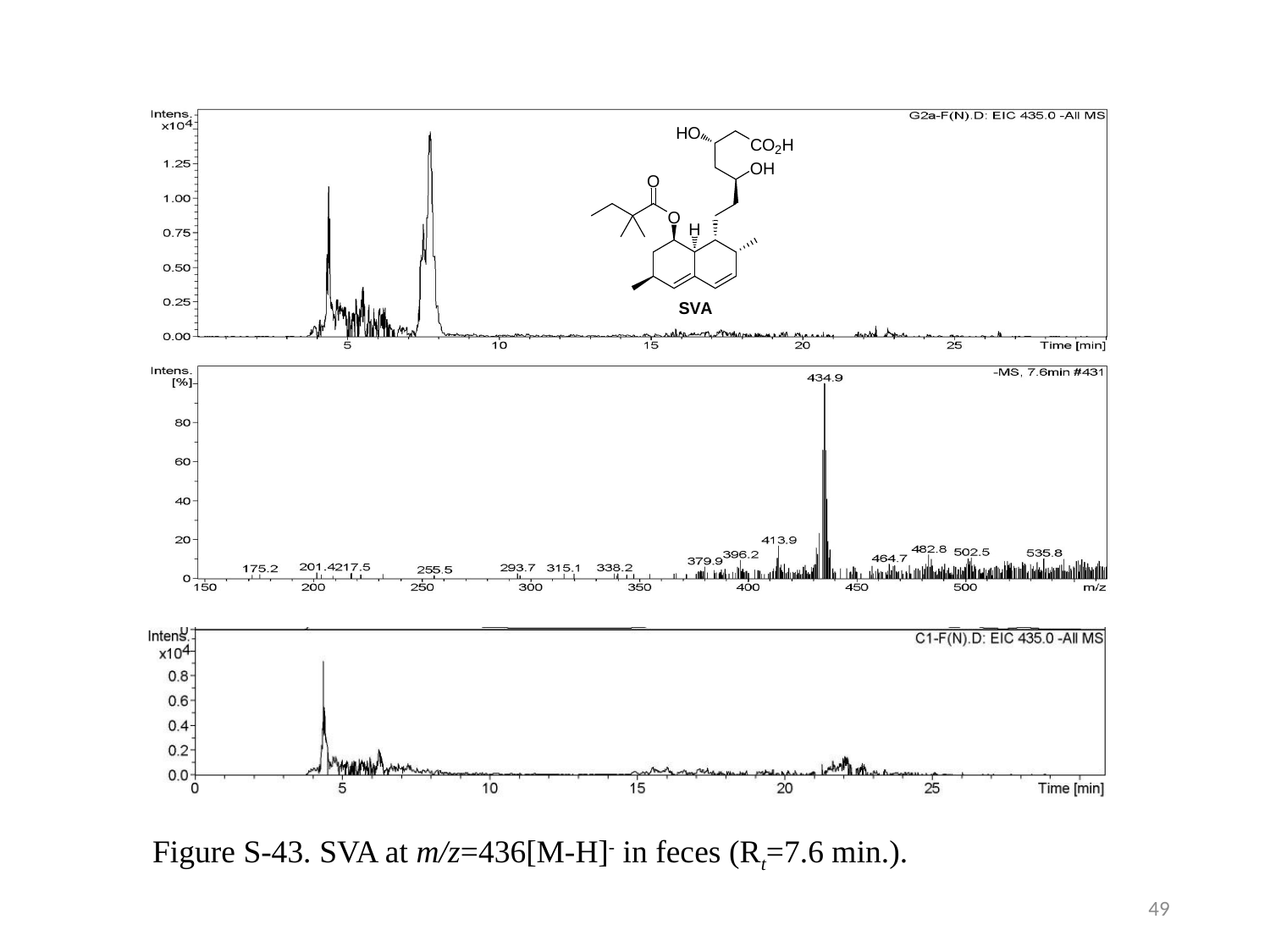

Figure S-43. SVA at m/z=436[M-H]- in feces (Rt=7.6 min.).
49

## Slide 57
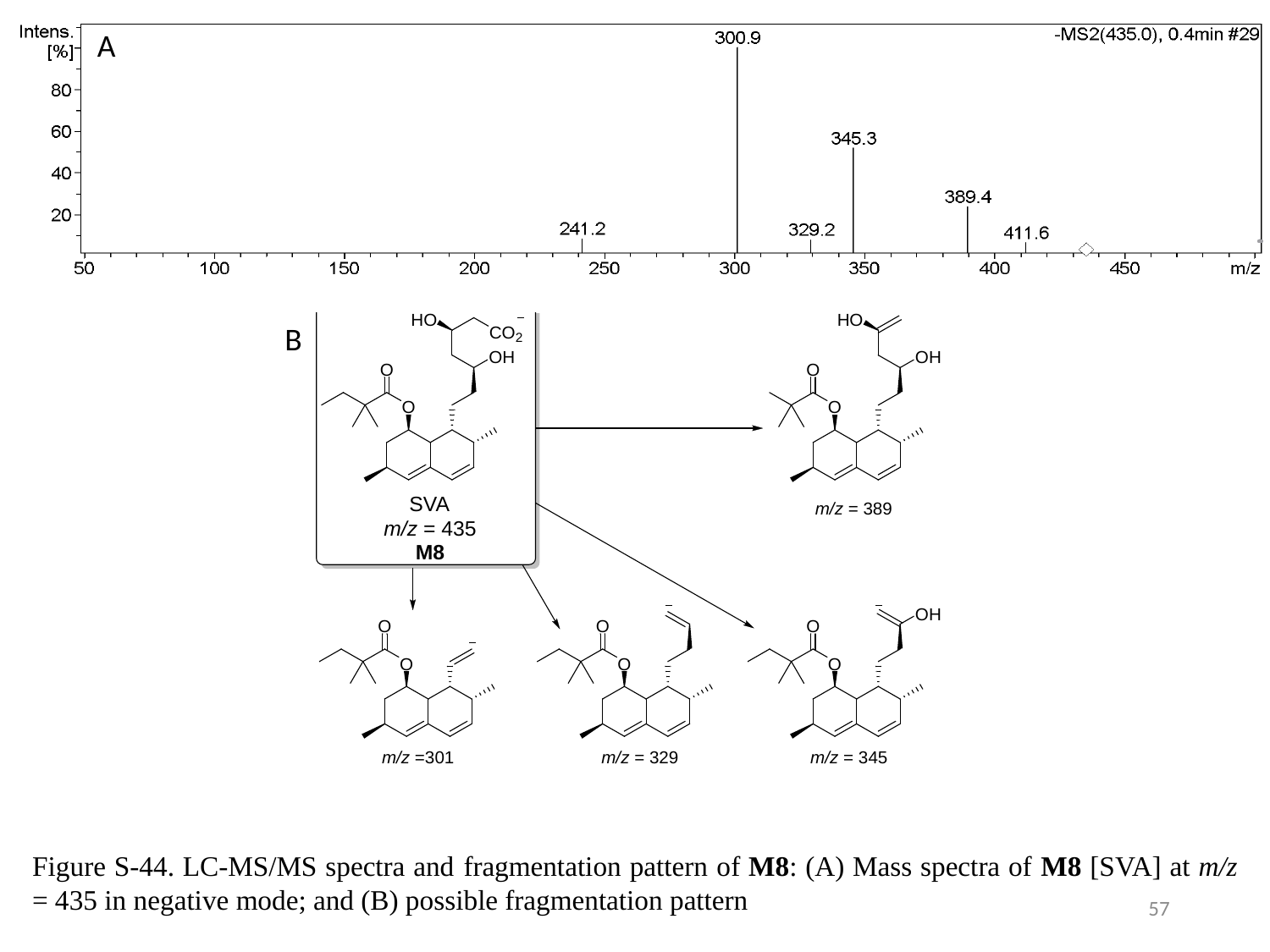

A
B
Figure S-44. LC-MS/MS spectra and fragmentation pattern of M8: (A) Mass spectra of M8 [SVA] at m/z = 435 in negative mode; and (B) possible fragmentation pattern
57

## Slide 58
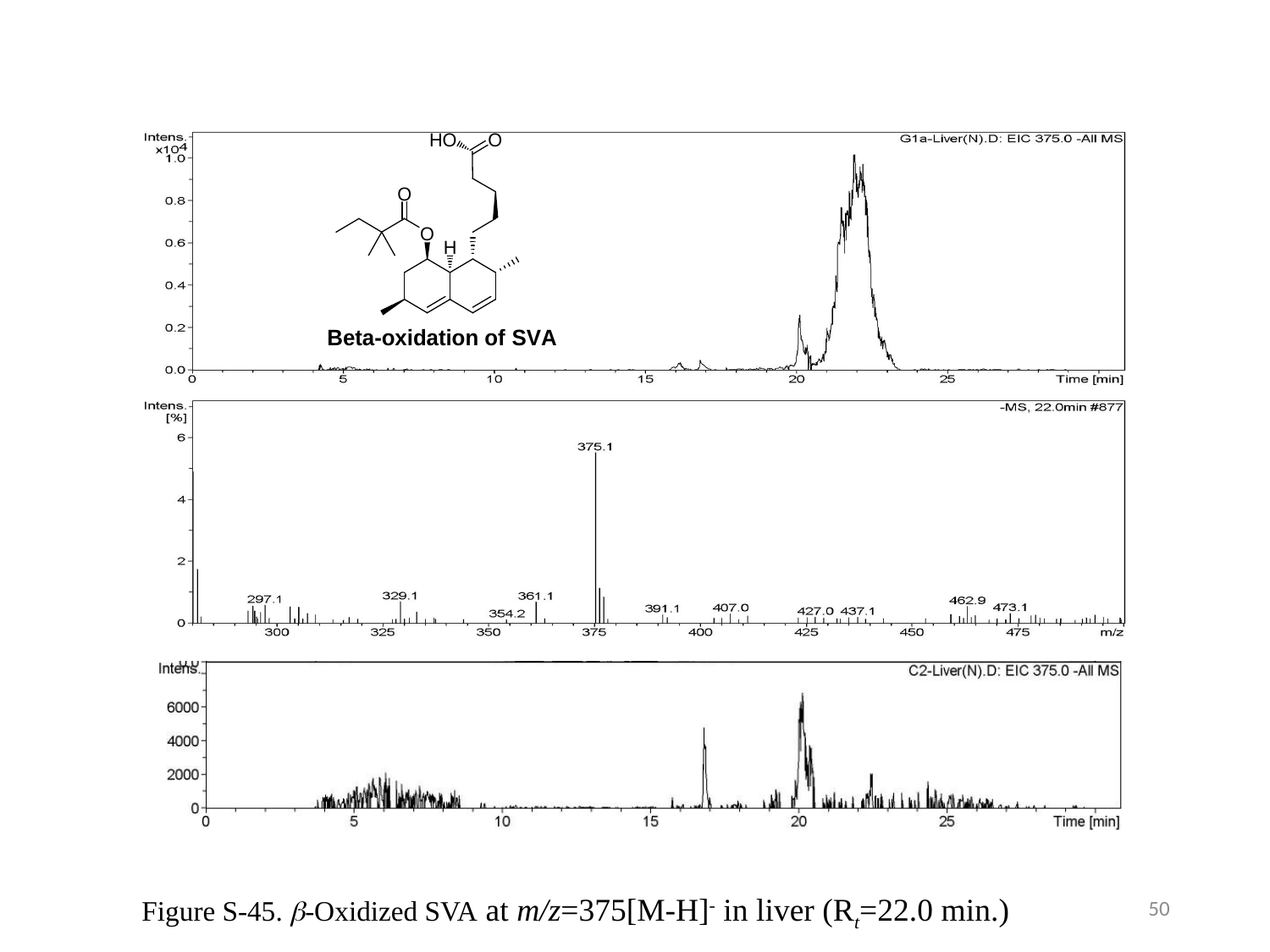

Figure S-45. β-Oxidized SVA at m/z=375[M-H]- in liver (Rt=22.0 min.)
50

## Slide 59
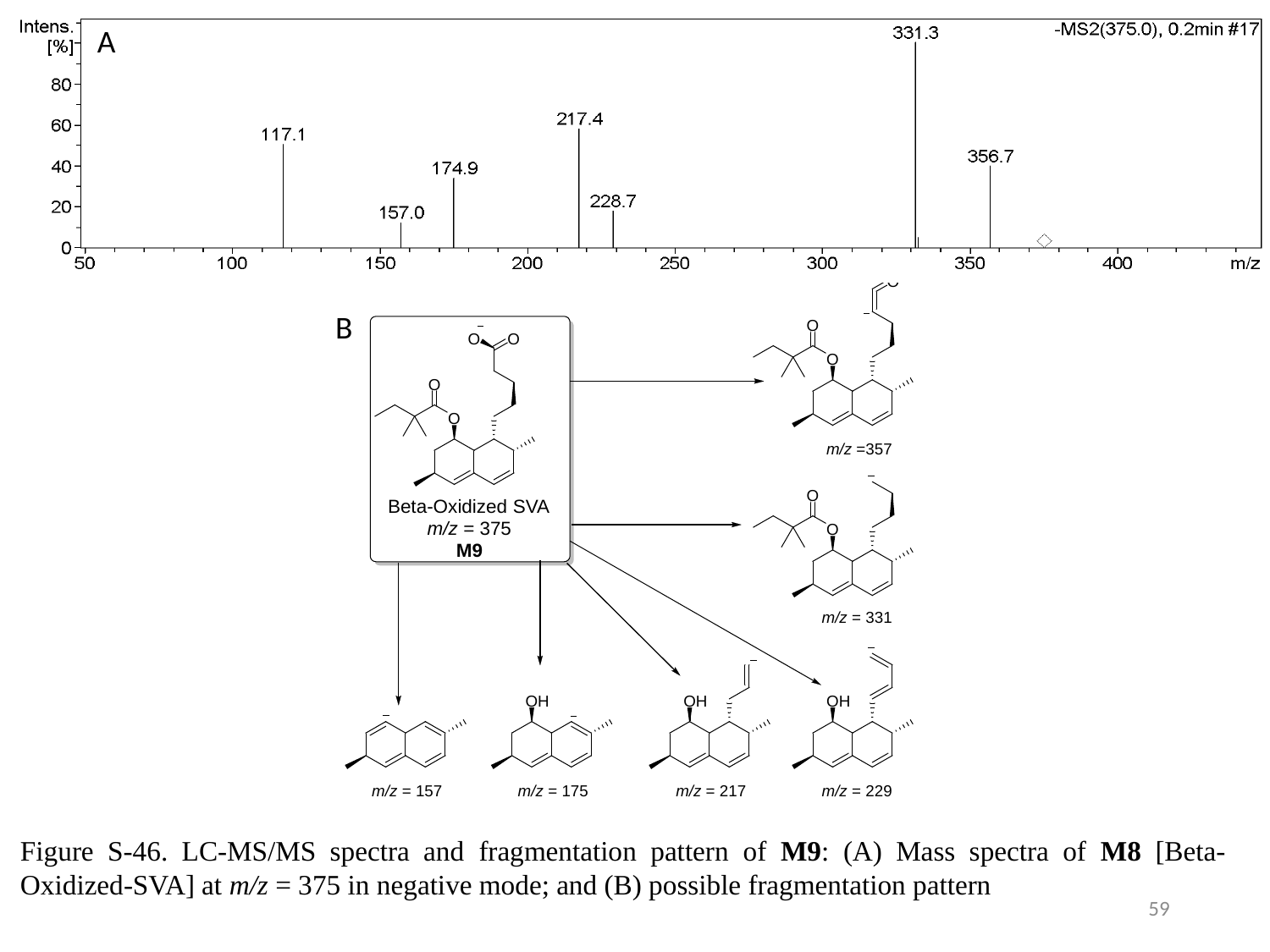

A
B
Figure S-46. LC-MS/MS spectra and fragmentation pattern of M9: (A) Mass spectra of M8 [Beta-Oxidized-SVA] at m/z = 375 in negative mode; and (B) possible fragmentation pattern
59
